# Prostaglandin E2-mediated aging microenvironment fuels pathogenic fibrosis in cesarean section scar defect

**DOI:** 10.1038/s44321-026-00521-6

**Published:** 2026-10-02

**Authors:** Yujia Yin, Huihui Chen, Weiwei Xie, Yizhi Wang, Yujing Qian, Rong Sheng, Danning Li, Jiarui Li, Xingchen Zhou, Zhiyuan Dai, Zhiyong Mao, Ziyi Li, Xipeng Wang

**Affiliations:** 1 Department of Obstetrics and Gynecology, Xinhua Hospital Affiliated to Shanghai Jiao Tong University School of Medicine, Shanghai, China; 2 Shanghai Key Laboratory of Maternal Fetal Medicine, Clinical and Translational Research Center of Shanghai First Maternity and Infant Hospital, Frontier Science Center for Stem Cell Research, School of Life Sciences and Technology, Tongji University, Shanghai, China; 3 National Key Laboratory of Immunity and Inflammation, Suzhou Institute of Systems Medicine, Chinese Academy of Medical Sciences & Peking Union Medical College, Suzhou, China

**Keywords:** Stem Cells & Regenerative Medicine

## Abstract

With the increasing rates of cesarean section (CS), cesarean section scar defect (CSD) has become an escalating clinical concern due to uterine dysfunction and reduced fertility. However, the biological basis of CSD remains poorly understood, particularly the mechanisms of impaired uterine repair after CS and the causes of failure in conventional repair surgeries. This study integrates metabolomics, transcriptomics, a newly developed mouse model, and functional assays to elucidate the complex interplay between senescence and fibrosis in CSD. Our findings identify upregulation of the PGES/prostaglandin E2 (PGE2) axis as a key upstream event that disrupts uterine healing. Elevated PGE2 preferentially induces early senescence of macrophages, which transmit pro-senescent signals to uterine fibroblasts through IL-1β/IL-1R signaling. This sequential senescence cascade drives excessive fibroblast activation and progressive fibrosis, ultimately impairing uterine structure and function. In mouse CS models, inhibition of the PGES/PGE2 pathway and anti-aging strategies significantly attenuate fibrosis and restore coordinated myometrial and endometrial repair. Our findings uncover a macrophage–fibroblast senescence relay to fibrotic scar formation in CSD and provide a translational rationale for targeting PGE2 signaling and senescence to improve uterine repair after CS.

## Synopsis

Cesarean section scar defect (CSD) is a clinically common disorder of uterine wound healing after cesarean section, yet its biological basis has been overlooked. This study shows that targeting PGE2-driven aging microenvironment attenuates fibrosis and improves uterine repair in preclinical models.

- The PGES/PGE2 axis was identified as an upstream driver of poor uterine wound healing in CSD.
- Early senescence was preferentially induced in IL1B^+^ macrophages by elevated PGE2, establishing a cellular source of sustained pro-senescent and pro-fibrotic signaling.
- Fibroblast senescence and excessive activation were propagated through macrophage-derived IL-1β/IL-1R signaling.
- Targeting PGE2 signaling or aging could reduce uterine fibrosis and improve coordinated myometrial and endometrial repair in mouse models.

## Abstract (web summary)

Cesarean section scar defect (CSD) is a clinically common disorder of uterine wound healing after cesarean section, yet its biological basis has been overlooked. This study shows that targeting PGE2-driven aging microenvironment attenuates fibrosis and improves uterine repair in preclinical models.

## Graphical abstract

**Figure:**
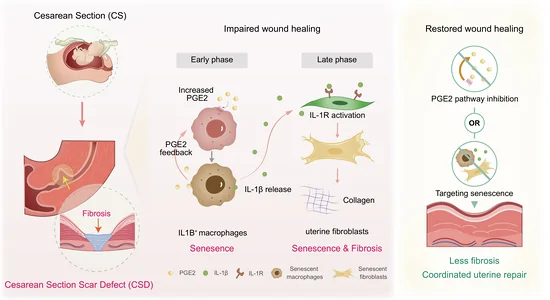

The paper explained **Problem**

Cesarean section scar defect (CSD) is an increasingly common complication of cesarean delivery that can cause abnormal uterine bleeding, pelvic symptoms, and impaired fertility. Surgical repair can restore the anatomy of the uterine scar, but healing remains incomplete in some patients, suggesting that correcting the structural defect alone may not address the underlying biological abnormalities. The mechanisms that disrupt normal uterine wound healing after cesarean section and promote persistent scar fibrosis remain poorly understood, limiting the development of mechanism-based treatments for CSD.

**Results**

We identified increased prostaglandin E synthase (PGES)/prostaglandin E2 (PGE2) signaling as an important upstream event associated with defective uterine repair in CSD. Using human samples, cellular experiments, and mouse models, we found that elevated PGE2 preferentially promotes senescence of macrophages. These macrophages subsequently communicate with uterine fibroblasts through IL-1β/IL-1R signaling, promoting fibroblast senescence and excessive activation. This macrophage–fibroblast senescence relay enhances extracellular matrix deposition and progressive fibrosis at the uterine scar. In mouse models, targeting the PGES/PGE2 pathway or cellular senescence reduced fibrosis and improved coordinated repair of the myometrium and endometrium.

**Impact**

Our study provides a mechanistic explanation for how persistent inflammatory and senescence-associated signaling can convert physiological uterine wound healing into pathological fibrosis after cesarean section. The identification of a PGE2-driven macrophage–fibroblast senescence relay suggests that CSD is not solely an anatomical scar defect but also a disorder of the uterine microenvironment. These findings provide a rationale for future evaluation of therapies targeting PGE2 signaling, senescence, or associated inflammatory communication as adjunct approaches to improve uterine repair after cesarean section.

## Introduction

Cesarean section scar defect (CSD) is a long-term complication of cesarean section (CS), characterized by the unhealing of the myometrium and the formation of a symptomatic uterine niche at the site of CS (Klein Meuleman et al, 2023). CSD affects 50% to 84% of women with a history of CS (Zhou et al, 2024). This condition leads to various clinical symptoms, including prolonged menstrual bleeding, dysmenorrhea, secondary infertility, scar pregnancy, and psychological disorders (Stegwee et al, 2020). Conventional approaches encompass a spectrum of medical interventions to alleviate abnormal uterine bleeding and spotting, including the use of oral contraceptives and the application of a levonorgestrel intrauterine system (LUS). In addition, surgical techniques such as laparoscopic, hysteroscopic, and transvaginal procedures are utilized for repair. These reconstructive surgeries for uterine myometrium effectively remove niches in CSD patients, leading to improved gynecological and obstetric outcomes (Tsuji et al, 2023). However, some patients experience persistent symptoms despite multiple operations, suggesting the involvement of idiopathic factors in CSD development and symptomatology.

Early studies on the etiology identified significant associations between CSD and physical factors such as uterine structure, suturing methods, timing of delivery, and neonatal weight (Antila-Långsjö et al, 2018; Zhou et al, 2024). Histopathological studies of CSD tissues further revealed characteristic abnormalities, like absence or thinning of the myometrium and excessive deposition of fibrotic tissue within stromal congestion, endometrial fragmentation, and the presence of adenomyosis (Chen et al, 2022; Donnez, 2020). Importantly, the size of the niche formed by this aberrant fibrotic tissue is strongly and positively correlated with clinical symptoms such as abnormal uterine bleeding and infertility (Donnez, 2020; Zhou et al, 2017). Collectively, these features suggest that the extent of fibrosis is closely linked to structurally disrupted endometrial and myometrial integrity, a pathological condition thought to underlie the uterine symptomatic dysfunction commonly observed in CSD patients (Higuchi et al, 2022). Thus, elucidating the mechanisms that drive uterine incision fibrosis is essential for developing targeted strategies to alleviate clinical symptoms.

Despite its clinical relevance, the biological processes governing excessive fibrosis at the uterine incision site in CSD remain largely undefined. To address this gap, we performed quasi-targeted metabolomics on CSD tissues to identify potential pathogenic signaling pathways within the uterus (Tokarz et al, 2020). This analysis revealed the upregulation of the PGES/PGE2 axis in CSD tissues. Prostaglandin E2 (PGE2), a major product of arachidonic acid metabolism, is widely recognized as a regulator of female reproductive physiology and modulates diverse cellular functions, including cellular senescence and differentiation (Ricciotti and FitzGerald, 2011; Cheng et al, 2021). Building on these findings, we investigated the mechanistic role of PGE2 in driving cellular senescence and fibrotic remodeling, focusing on its crosstalk with macrophages and uterine fibroblasts during uterine injury repair to elucidate the pathogenesis of CSD following CS. By defining this pathogenic circuit, we sought to uncover a biologically tractable mechanism underlying CSD and to establish a rationale for targeted therapeutic intervention to improve uterine repair and reproductive outcomes after cesarean section.

## Results

### PGES/PGE2 axis rises in CSD patients

**Figure EV1:**
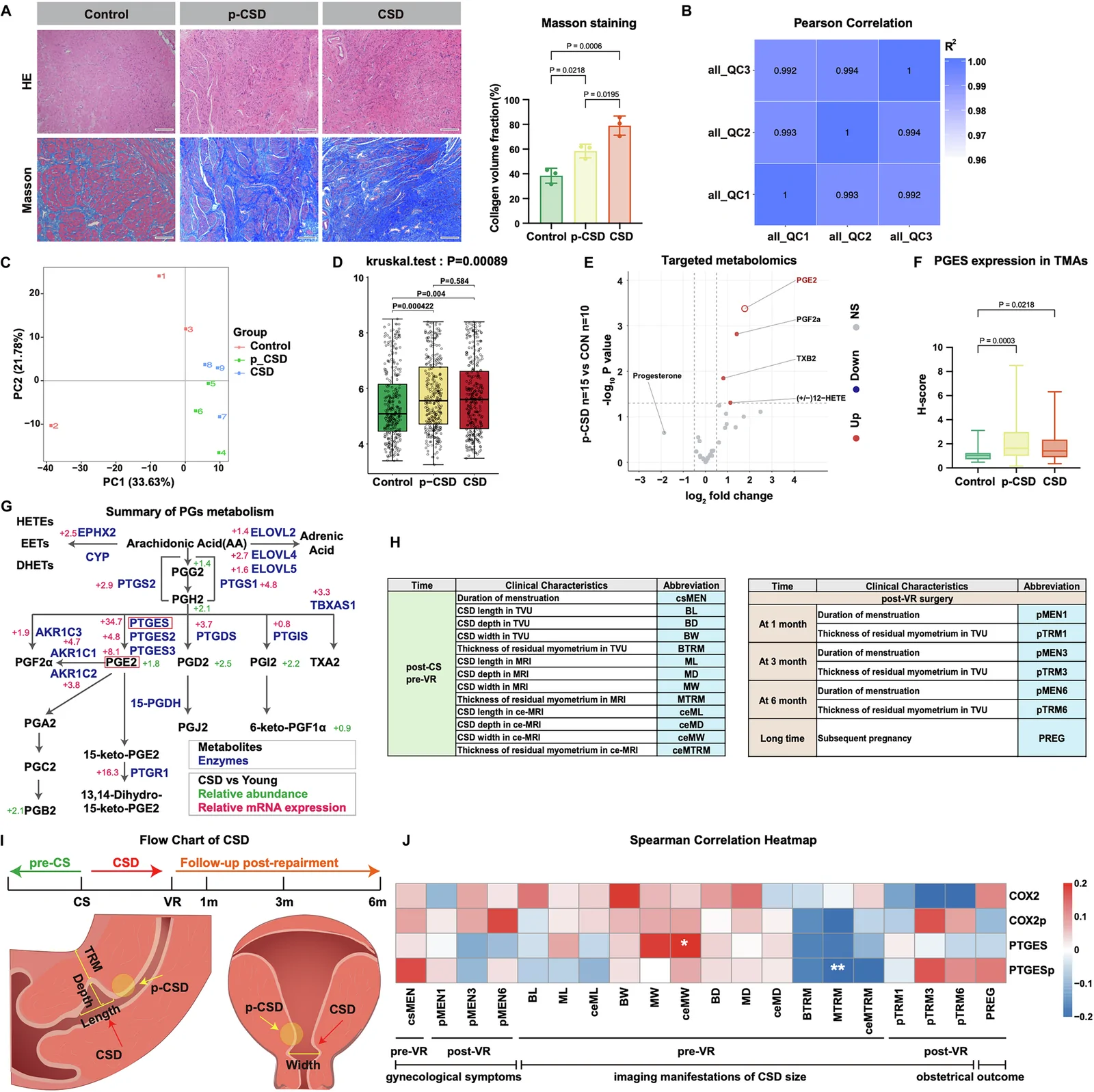
Correlation of PGES/PGE2 expression with adverse clinical features in CSD patients. (**A**) Representative images of HE and Masson staining (left) and comparison of collagen volume fraction by Masson staining (right) among paired CSD and para-CSD tissues, healthy controls. Scale bars, 200 μm. (*n* = 3 per group). (**B**, **C**) Quality control and principal component analysis (PCA) of quasi-targeted metabolomics in human uterine tissues. (**D**) Statistical comparison of metabolite profiles among CSD, para-CSD tissues, and healthy controls (*n* = 3). (**E**) Volcano plot showing differential metabolites detected by targeted arachidonic acid metabolomics in para-CSD tissues (*n* = 15) and healthy control uterine tissues (*n* = 10). (**F**) H-score quantification of PGES protein expression in human myometrium TMAs (*n* = 118 for CSD and 37 for CON). (**G**) Prostaglandin metabolic pathway diagram highlighting key metabolites (black), enzymes (blue), relative abundance (green), and mRNA expression (pink) in CSD versus healthy controls. (**H**) Tables of clinical characteristics during the diagnosis and treatment of CSD. Left panel, baseline values before VR surgery; right panel, follow-up values after VR surgery. (**I**) Flowchart of clinical management from cesarean section to post-vaginal repair follow-up in CSD patients. (**J**) Heatmap of Spearman correlations between COX2/PGES protein expression in paired CSD and para-CSD TMAs and clinical features, with color intensity denoting correlation coefficients. *P < 0.05, **P < 0.01. Data are presented as box plots or mean ± s.d., showing the median, interquartile range, and whiskers representing the minimum to maximum values. One-way ANOVA with Tukey’s multiple comparisons test (**A**, **F**); Welch’s two-sample *t* test (**E**); Kruskal–Wallis test (**D**); Spearman's correlations analysis (**J**). Biological replicates represent individual patients, with exact numbers listed in each panel. Source data are available online for this figure.

**Figure 1:**
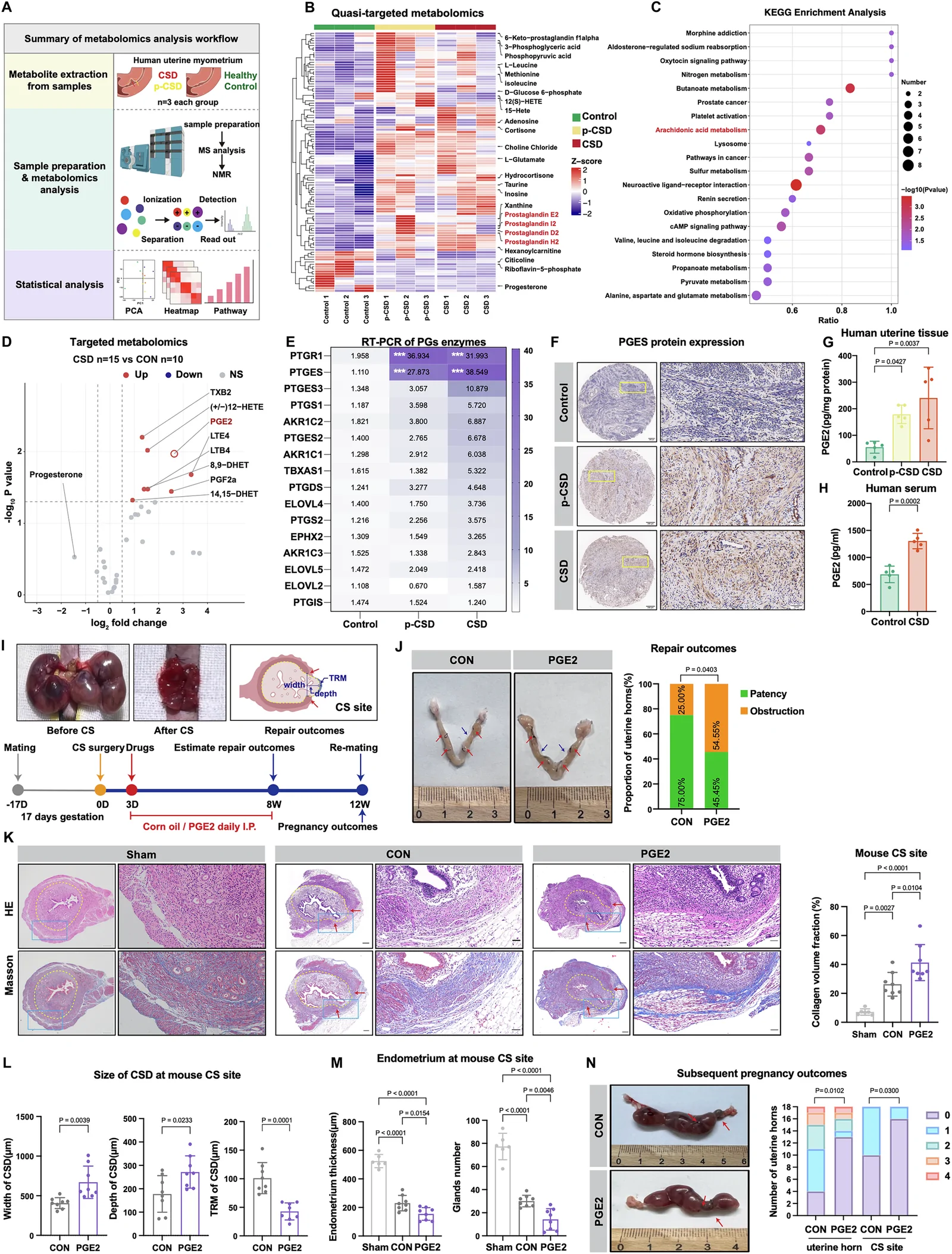
Upregulation of the PGES/PGE2 axis accelerates uterine incision fibrosis post-cesarean section. (**A**) Flowchart of sample collection and quasi-targeted metabolomics analysis. (**B**) Heatmap of differential metabolite profiles between paired CSD, para-CSD, and healthy controls via Quasi-targeted metabolomics (*n* = 3 per group). (**C**) KEGG pathway enrichment analysis of differentially expressed metabolites identified by metabolomics. (**D**) Volcano plot showing differential metabolites detected by targeted arachidonic acid metabolomics in CSD tissues (*n* = 15) and healthy control uterine tissues (*n* = 10). (**E**) Heatmap showing relative mRNA expression of prostaglandin-associated enzymes in uterine tissues (*n* = 3 per group). The numbered color scale represents relative mRNA expression. ***P < 0.001. (**F**) Representative IHC images of PGES protein expression in human myometrium TMAs. Scale bars, 200 μm and 50 μm. (**G**, **H**) Quantification of PGE2 in human uterine tissues and serum (*n* = 5 per group) via ELISA. (**I**) Timeline detailing the establishment and recovery analysis of mouse CS models under PGE2 treatment. (**J**) Gross morphological images of uteri and patency analysis in mouse CS models at 8 weeks post-CS (*n* = 11–12 per group). Red arrows indicate the ends of the CS sutures; blue arrows highlight obstructions. (**K**, **L**) Representative images of HE and Masson staining (**K**, left) with quantification of collagen volume fraction (**K**, right) and sizes of injury sites (**L**) at the CS sites at 8 weeks post-CS (*n* = 6–8 per group). Scale bars, 200 and 50 μm. Yellow circles demarcate the boundary between the endometrium and myometrium, highlighting the extent of endometrial invasion at the injury site. Red arrows indicate the location of the uterine incision. Sham vs PGE2, *P* = 3.81 × 10⁻⁶. (**M**) Quantification of endometrium thickness and gland number at the CS sites at 8 weeks post-CS (*n* = 6–8 per group). For endometrial thickness, *P* = 2.30 × 10⁻⁹ and 4.74 × 10⁻¹¹ for Sham vs CON and Sham vs PGE2, respectively; for gland number, *P* = 1.42 × 10⁻⁸ and 1.11 × 10⁻¹⁰. (**N**) Representative images of pregnant uteri (left) and quantification of fetuses in uterine horns and CS sites (right) (*n* = 9 per group). Red arrows indicate the location of the uterine incision. Data are mean ± s.d. One-way ANOVA with Tukey’s multiple comparisons test (**B**, **G**, **K**, **M**); KEGG pathway enrichment was assessed using a hypergeometric test, with *P* values adjusted for multiple comparisons using the Benjamini–Hochberg method (**C**); Welch’s two-sample *t* test (**D**); two-way ANOVA with Dunnett’s post hoc test for multiple comparisons (**E**); Chi-squared test (**J**); two-Sided unpaired *t* test (**H**, **L**); Mann–Whitney test with one-tailed *P* value (**N**). Biological replicates represent individual patients, with exact numbers listed in each panel. Source data are available online for this figure.

CSD represents a symptomatic outcome of impaired uterine incision healing (Tower and Frishman, 2013), characterized by excessive fibrotic deposition at the CS site (Fig. EV1A). To investigate the mechanisms driving fibrosis development in CSD, we performed Quasi-targeted metabolomics on paired CSD and para-CSD tissues from patients, as well as healthy controls (Figs. 1A and EV1B; Dataset EV1). Metabolic profiling revealed that both CSD and para-CSD tissues were markedly distinct from healthy controls, while showing no significant differences between each other (Fig. EV1C,D). Notably, prostaglandin family metabolites, as main members of the arachidonic acid metabolism pathway (Li et al, 2021), were significantly upregulated, with overall pathway enrichment confirmed (Fig. 1B,C). Prostaglandins generated by cyclooxygenase-1/2 (COX-1/2) are well-established regulators of female reproductive endocrine function (Wang et al, 2025). To further validate the dysregulation of arachidonic acid metabolism in CSD tissues, we performed targeted metabolomic analysis of arachidonic acid pathway metabolites in an expanded cohort. Based on the quasi-targeted metabolomic results, progesterone and taurohyodeoxycholic acid, which were enriched in healthy control tissues, were additionally included in the targeted validation panel. Targeted metabolomic analysis demonstrated that PGE2, PGF2α, TXB2, and 12-HETE levels were significantly elevated in both CSD and para-CSD tissues compared with healthy controls, whereas progesterone levels showed a consistent downward trend in CSD-associated tissues (Figs. 1D and EV1E; Dataset EV1). Among them, PGE2 has been shown to be elevated in chronic non-healing wounds and fibrotic diseases, including liver and lung fibrosis (Dagouassat et al, 2013; Davis et al, 2020; Nishizawa et al, 2018). Consistent with these observations, our RT–PCR analysis demonstrated a significant upregulation of *PTGES* (encoding PGES, the rate-limiting synthase for PGE2 (McCann et al, 2011)) and *PTGR1* (encoding prostaglandin reductase 1, a key enzyme involved in downstream PGE2 catabolism (Wang et al, 2021)) in both CSD and para-CSD tissues, indicating that the PGES-driven PGE2 axis is markedly elevated in CSD (Fig. 1E). To validate these findings at the protein level, we performed PGES immunohistochemistry (IHC) on tissue microarrays (TMAs) of CSD, para-CSD, and control tissues, and observed robust PGES upregulation in both CSD and para-CSD regions (Figs. 1F and EV1F). ELISA further confirmed significantly increased PGE2 levels in CSD tissues and in the serum of CSD patients (Fig. 1G,H). Together, these results suggest that upregulation of the PGES/PGE2 axis is strongly associated with disease pathogenesis in CSD (Fig. EV1G).

To ascertain the relationship between the PGES/PGE2 axis and disease severity and prognosis in CSD patients, we analyzed clinical data from TMAs, encompassing menstrual patterns, niche-related imaging parameters (length, width, depth, and thickness of residual myometrial (TRM)) before and after repair, as well as long-term subsequent pregnancy outcomes after repair surgery (Fig. EV1H,I). Correlational analyses of TMAs IHC outcomes with these clinical data from CSD patients revealed a significant positive correlation between PGES upregulation and preoperative niche width, and a significant negative correlation with TRM (Fig. EV1J). These findings highlight the crucial role of the PGES/PGE2 axis in the formation of pathological fibrosis at uterine incision sites post-CS.

### Exogenous PGE2 exposure exacerbates fibrosis and uterine dysfunction post-CS

**Figure EV2:**
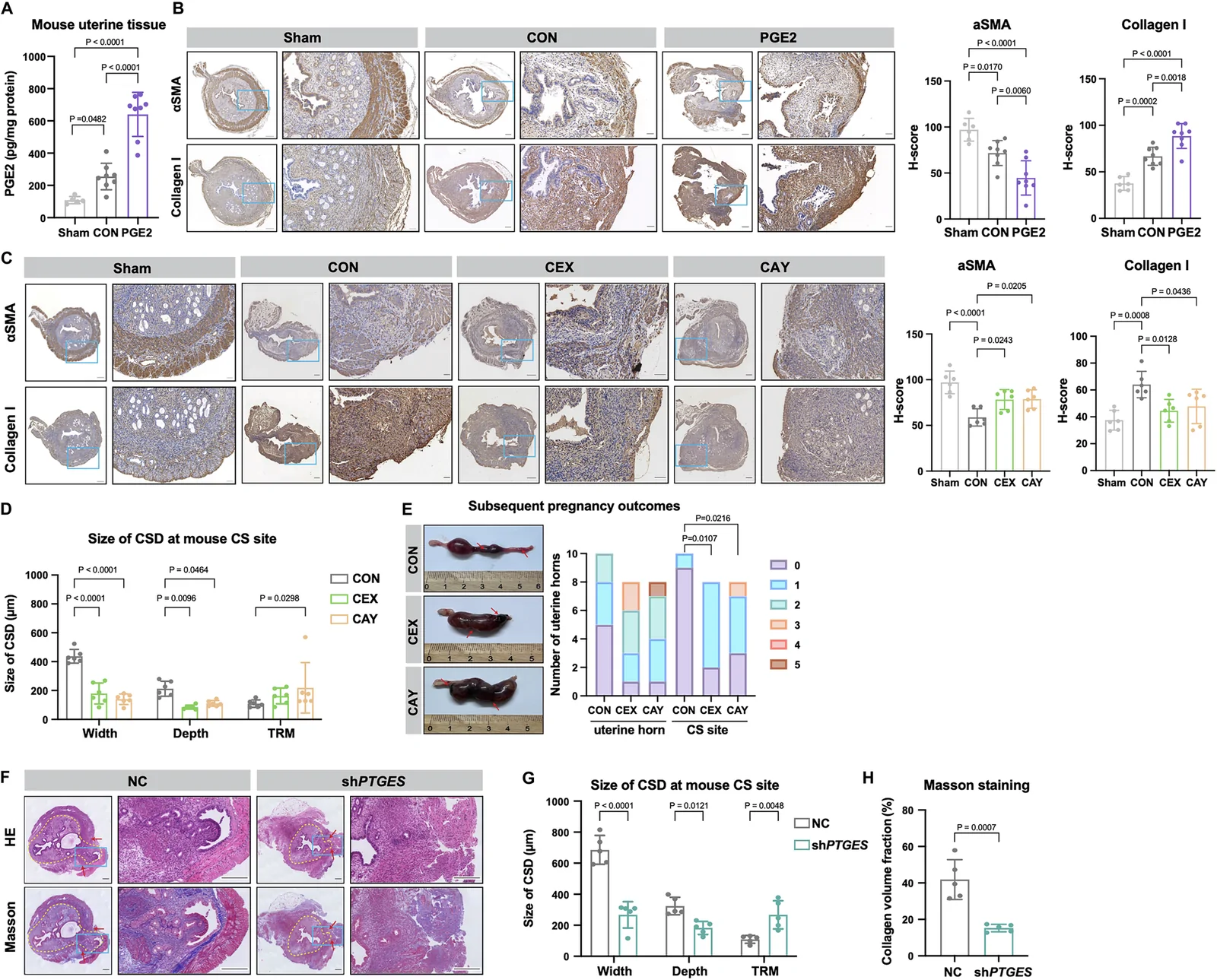
Impacts of the COX2/PGES/PGE2 axis on uterine recovery post-CS. (**A**) Quantification of PGE2 levels in uterine tissues from mouse CS models using ELISA at 8 weeks post-CS (*n* = 5–8 per group). *P* = 1.21 ×  10⁻⁶ for CON versus PGE2 and *P* = 7.50 × 10⁻⁸ for Sham versus PGE2. (**B**) Representative IHC images of αSMA and collagen I at CS sites in PGE2-treated mice 8 weeks post-CS (left) with quantification shown via H-scores (right) (*n* = 6–8 per group). Scale bars, 200 and 50 μm. For Sham versus PGE2, *P* = 1.29 × 10⁻⁵ for αSMA and *P* = 1.13 × 10⁻⁷ for collagen I. (**C**) Representative IHC images (left) and H-score of αSMA and Collagen I expression (right) in COX2/PGES inhibitors-treated mouse CS uterine tissues at 8 weeks post-CS (*n* = 6 per group). Scale bars, 200 and 50 μm. (**D**) Quantification of sizes of CSD at CS sites 8 weeks post-CS (*n* = 6 per group). For CSD width, *P* = 4.03 × 10⁻⁷ and 1.57 × 10⁻⁸ for CON versus CEX and CON versus CAY, respectively. (**E**) Representative images of pregnant uteri (left) and quantification of fetuses in uterine horns and CS sites (right) (*n* = 4–5 per group). Red arrows indicate the location of the uterine incision. (**F**) Representative images of HE and Masson staining of *PTGES* knockdown in vivo at mouse CS sites at 8 weeks post-CS. Scale bars, 200 and 50 μm. Yellow circles demarcate the boundary between the endometrium and myometrium. Red arrows indicate the location of the uterine incision. (**G**) Quantification of sizes of CSD at CS sites 8 weeks post-CS (*n* = 5 per group). For CSD width, *P* = 4.63 × 10⁻⁹ for NC versus sh*PTGES*. (**H**) Quantification of collagen volume fraction at CS sites at 8 weeks post-CS (*n* = 5 per group). Data are mean ± s.d. One-way ANOVA with Tukey’s multiple comparisons test (**A**–**C**); two-way ANOVA with Tukey’s or Šídák’s multiple comparisons test (**D**, **G**); unpaired *t* test (**H**), Kruskal–Wallis test (**E**). Independent biological samples were used; exact numbers of biological replicates are listed in each panel. Source data are available online for this figure.

To elucidate the mechanisms underlying fibrotic repair in CSD, we first innovatively established a murine CS model. Pregnant mice at embryonic day 17 (E17) underwent bilateral longitudinal uterine incisions, after which uterine healing was evaluated at 8 weeks (Fig. 1I). Compared with sham-operated controls, CS-operated mice exhibited significantly increased fibrosis at the incision site, accompanied by elevated PGE2 levels, indicating that this murine CS model effectively recapitulates features of uterine scar healing in CSD (Figs. 1J,K and EV2A).

To further investigate the role of elevated PGE2 in amplifying uterine incision fibrosis, CS mice were treated with daily intraperitoneal PGE2 administration (Fig. 1I). ELISA confirmed a significant increase in local PGE2 levels in PGE2-treated mice (Fig. EV2A). Patency assays further revealed increased rates of uterine obstruction in PGE2-treated mice (Fig. 1J). Histological analyses (H&E and Masson staining) demonstrated significantly enhanced fibrosis, deeper and wider defects, and reduced TRM at the incision sites of PGE2-treated mice (Fig. 1K,L). The IHC for α-smooth muscle actin (αSMA) and collagen I corroborated these findings, showing reduced myometrial tissue and increased fibrotic deposition at 8 weeks post-CS in the PGE2-treated group (Fig. EV2B). Analyses of subsequent pregnancy outcomes revealed fewer pregnancies in both uterine horns and at CS sites in PGE2-treated mice, potentially resulting from reduced endometrial thickness and the number of glands observed in H&E staining (Fig. 1M,N). Together, these results indicate that upregulation of PGE2 after CS induces excessive fibrosis, disrupts normal uterine structure, and impairs uterine reproductive function.

### Inhibition of PGE2 promotes recovery of uterine structure and function after CS

**Figure 2:**
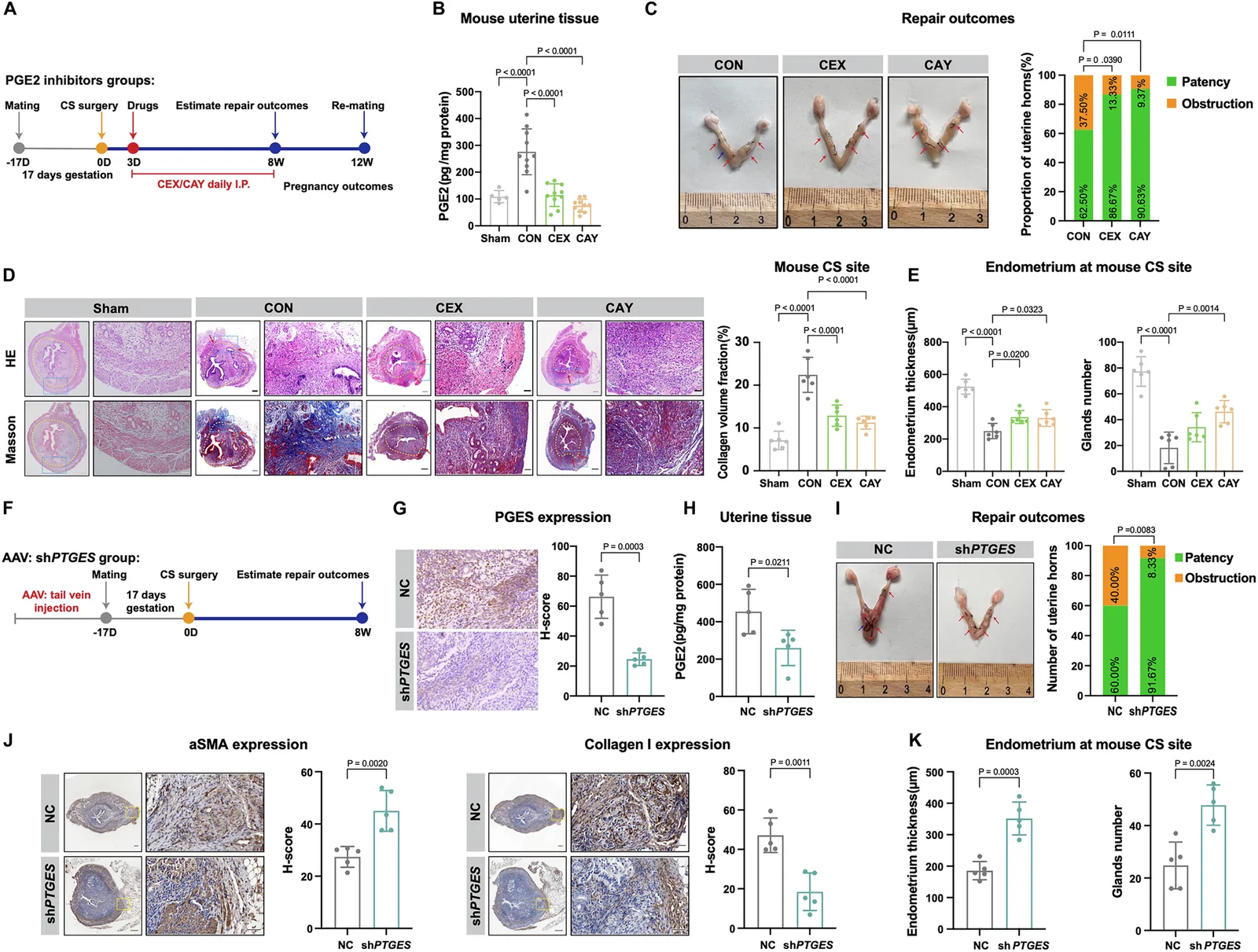
Targeting the COX2/PGES axis enhances uterine recovery post-CS in mouse models. (**A**) Timeline depicting the establishment and recovery analysis of mouse CS models treated with COX2/PGES inhibitors. (**B**) ELISA quantification of PGE2 levels in uterine tissues from CS models at 8 weeks post-CS (*n* = 5–10 per group). *P* = 1.66 × 10⁻⁵, 8.89 × 10⁻⁷, and 1.09 × 10⁻⁸ for Sham vs CON, CON vs CEX, and CON vs CAY, respectively. (**C**) Gross morphological images of uteri and patency analysis at 8 weeks post-CS (*n* = 12–16 per group), with red arrows indicating CS suture endpoints and blue arrows marking obstructions. (**D**) Representative histological images of HE and Masson staining and quantitative analyses of collagen volume fraction (*n* = 6 per group). Yellow circles demarcate the boundary between the endometrium and myometrium, highlighting the extent of endometrial invasion at the injury site. Red arrows indicate the location of the uterine incision. Scale bars, 200 and 50 μm. *P* = 3.09 × 10⁻⁸, 3.74 × 10⁻⁵, and 4.27  × 10⁻⁶ for Sham vs CON, CON vs CEX, and CON vs CAY, respectively. (**E**) Quantitative analyses of endometrium thickness and gland number at the CS sites at 8 weeks post-CS (*n* = 6 per group). For endometrial thickness and gland number, *P* = 1.23 × 10⁻⁸ and 5.67 × 10⁻⁸, respectively, for Sham vs CON. (**F**) Timeline depicting the establishment and recovery analysis of mouse CS models treated with AAV-sh*PTGES*. (**G**) Representative IHC images (left) and H-score analysis (right) of PGES expression in uterine tissues at 8 weeks post-CS (*n* = 5 per group). Scale bars, 50 μm. (**H**) ELISA quantification of PGE2 levels in uterine tissues from CS models at 8 weeks post-CS (*n* = 5 per group). (**I**) Gross morphological images of uteri and patency analysis at 8 weeks post-CS (*n* = 12–15 per group), with red arrows indicating CS suture endpoints and blue arrows marking obstructions. (**J**) IHC images and H-score analysis of αSMA (left) and collagen I (right) expressions in uterine tissues at 8 weeks post-CS (*n* = 5 per group). Scale bars, 200 and 50 μm. (**K**) Quantification of endometrium thickness and gland number in the mouse endometrium at CS sites 8 weeks post-CS (*n* = 5 per group). Data are mean ± s.d. One-way ANOVA with Tukey’s multiple comparisons test (**B**, **D**, **E**); unpaired *t* test (**G**, **H**, **J**, **K**); Chi-squared test (**C**, **I**). Independent biological samples were used; exact numbers of biological replicates are listed in each panel. Source data are available online for this figure.

To determine whether reducing PGE2 abundance could enhance myometrial healing and restore endometrial function post-CS, we intraperitoneally administered the COX-2 inhibitor celecoxib (CEX) and the PGES inhibitor CAY10526 (CAY) to CS mice (Fig. 2A). After 8 weeks of treatment, ELISA demonstrated significant reductions in PGE2 levels at the CS site in both CEX- and CAY-treated groups, accompanied by improved uterine patency rates (Fig. 2B,C). H&E and Masson staining further confirmed reduced fibrosis at the incision site, with corresponding decreases in residual incision width and depth. The IHC for αSMA and collagen I supported these findings, revealing enhanced myometrial repair and reduced fibrotic deposition following CEX and CAY treatment (Figs. 2D and EV2C,D). Notably, the CAY-treated group exhibited a significant increase in TRM (Fig. EV2D). Evaluations of endometrial thickness and gland number also indicated improved endometrial recovery in both PGE2 inhibitor treatment groups (Fig. 2E). Consistent with these structural improvements, the number of pregnancies occurring at the CS site increased significantly in CEX- and CAY-treated mice at 12 weeks post-CS, whereas pregnancies in the uterine horn showed only an upward trend (Fig. EV2E).

Given the superior efficacy of PGES inhibition in restoring uterine function and the specificity of targeting PGE2 synthesis, we further examined whether silencing *PTGES* using adeno-associated virus delivery of sh*PTGES* could achieve comparable benefits (Fig. 2F). ELISA and IHC analyses showed reduced PGE2 concentrations and decreased PGES expression at the incision site in sh*PTGES*-treated mice (Fig. 2G,H). Mirroring the effects of pharmacological PGES inhibition, sh*PTGES* mice exhibited improved uterine patency, enhanced myometrial healing, reduced fibrosis, and increased TRM thickness (Figs. 2I,J and EV2F–H). Endometrial thickness and gland number were also significantly elevated following PGES silencing, indicating restoration of endometrial structure and function (Fig. 2K). Together, these findings demonstrate that downregulating the COX-2/PGES/PGE2 axis effectively alleviates fibrosis and promotes myometrial and endometrial recovery after CS, thereby re-establishing a uterine microenvironment more conducive to fertility.

### Persistent senescent cells (SnCs) link PGE2 signaling to excessive fibrosis in CSD

A transient accumulation of SnCs is an indispensable component during tissue repair (Henderson et al, 2020). The persistence of SnCs can be detrimental, leading to impaired tissue regeneration and fibrotic disease (Paramos-de-Carvalho et al, 2021). Notably, PGE2 is a well-recognized factor of senescence-associated secretory phenotype (SASP) and has been shown to activate cytokine signaling pathways in both physiological aging and pathological conditions (Srour et al, 2024; Wu and Meydani, 2004). These observations prompted us to hypothesize that persistent SnCs may represent a key mechanism linking PGE2 signaling to fibrotic progression in CSD.

**Figure 3:**
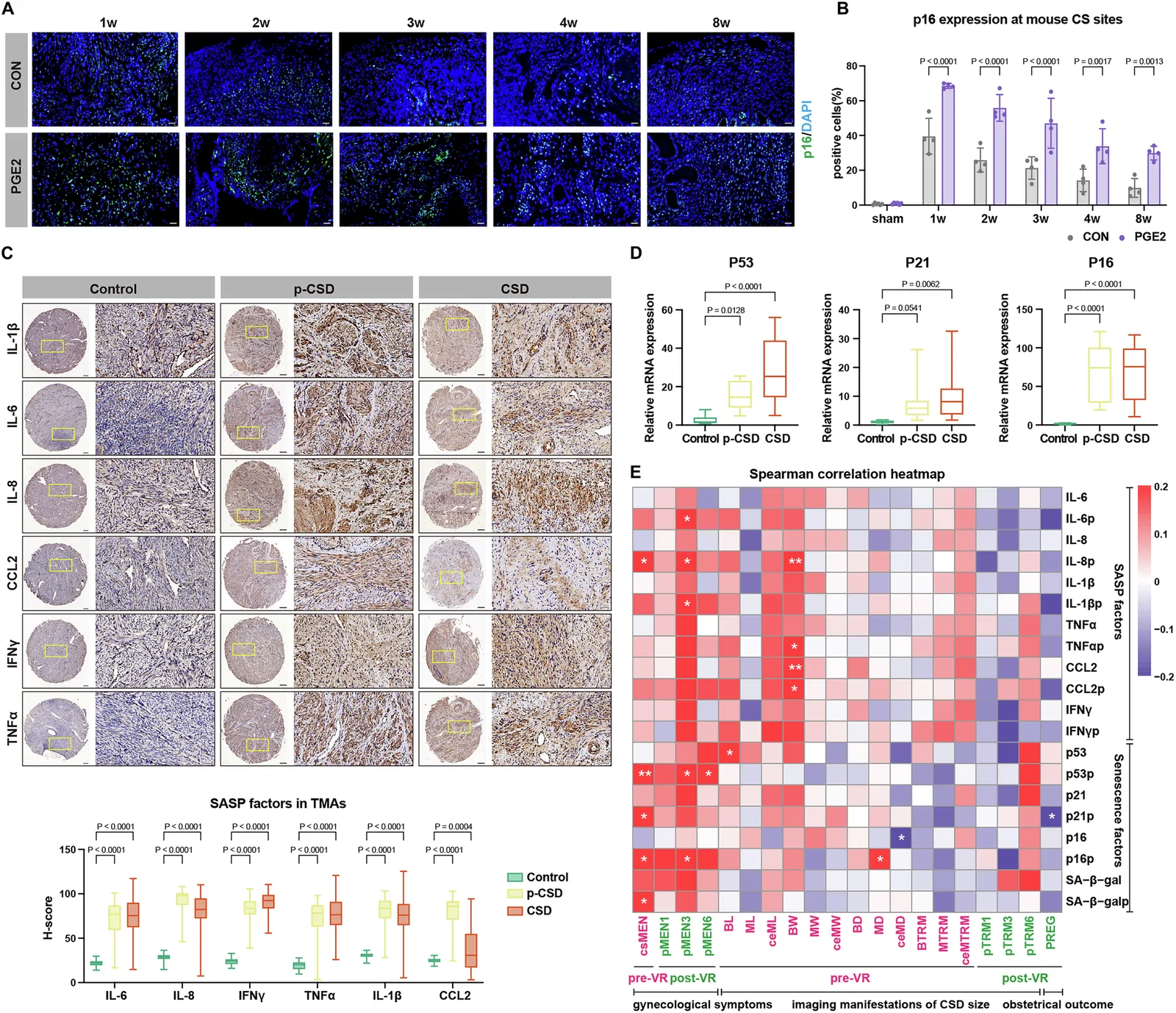
Identification of cellular senescence at uterine incision sites. (**A**, **B**) Representative images of mIF (**A**) and quantification of p16-positive cells as a percentage of total cells (**B**) at mouse CS sites over time in PGE2 treatment. Scale bars, 25 μm (*n* = 4–6 per group). For CON vs PGE2, *P* = 4.30 × 10⁻⁶, 2.24 × 10⁻⁶, and 3.77 × 10⁻⁵ at 1, 2, and 3 weeks, respectively. (**C**) Representative IHC images (upper panel) and H-score quantification (lower panel) of SASP proteins (IL-1β, IL-6, IL-8, CCL2, IFNγ, TNFα) in human myometrium TMAs. The representative images for the different markers were obtained from serial sections of the same tissue sample and were independently stained for the respective markers. Scale bars, 200 and 50 μm. (**D**) Relative mRNA expression levels of cellular senescence markers in human uterine tissues. For P53, *P* = 5.13 × 10⁻⁶ for control vs CSD; for P16, *P* = 4.25 × 10⁻⁵ and 4.68 × 10⁻⁵ for control vs p-CSD and control vs CSD, respectively. (**E**) Heatmap of Spearman correlations between IHC expression of cellular senescence and SASP markers in paired CSD and para-CSD TMAs and clinical characteristics of CSD patients. Clinical characteristics are detailed in Fig. EV1H. Color intensity indicates correlation coefficients. *P < 0.05, **P < 0.01. Exact *P* values are provided in the source data. Data are mean ± s.d or presented as box plots, showing the median, interquartile range, and whiskers representing the minimum to maximum values. One-way ANOVA with Tukey’s multiple comparisons test (**D**); Spearman correlations analysis (**E**); two-way ANOVA with Šídák’s multiple comparisons test (**B**); two-way ANOVA with Dunnett’s post hoc test for multiple comparisons (**C**). Biological replicates represent individual patients, with exact numbers listed in each panel. Source data are available online for this figure.

**Figure EV3:**
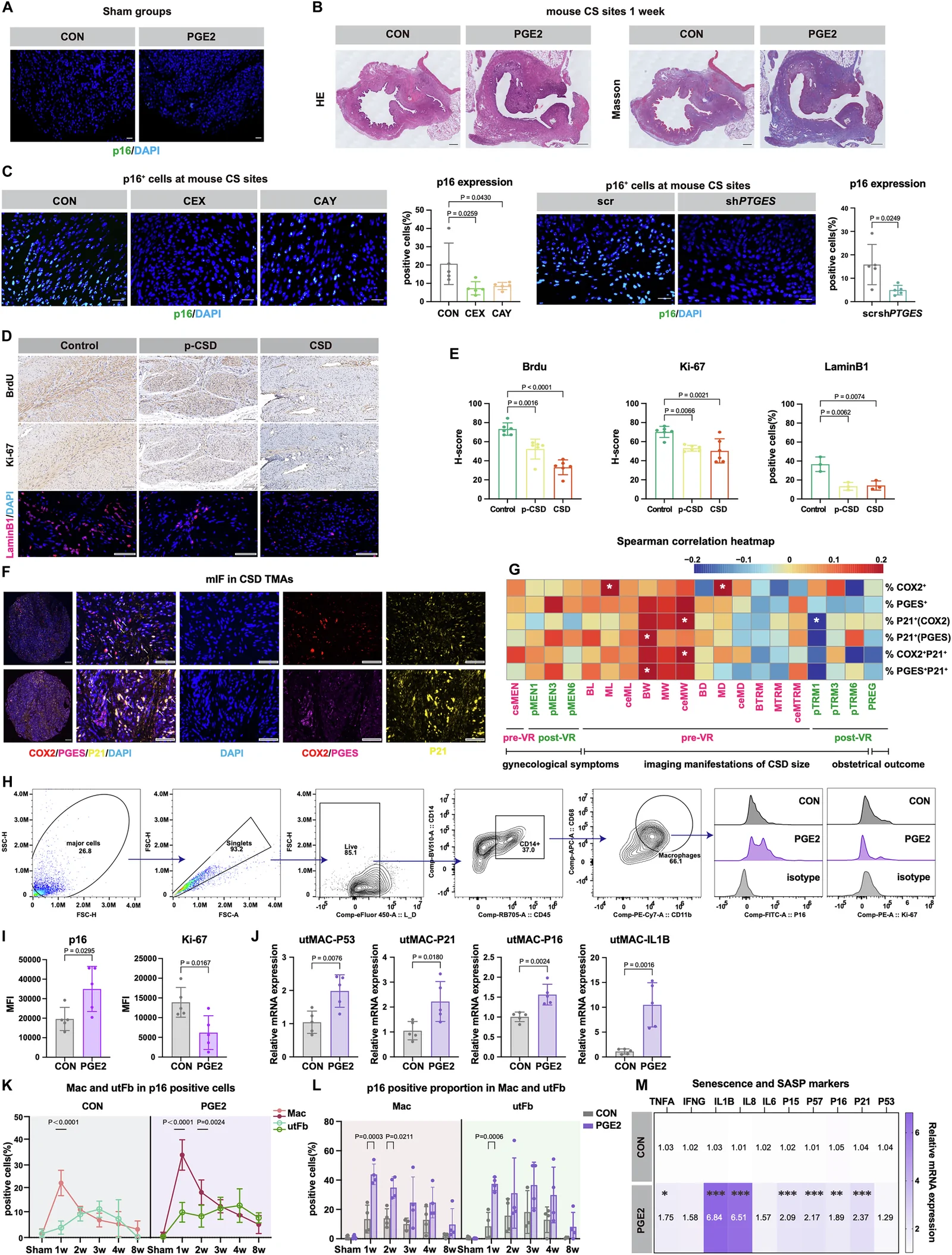
COX2/PGES axis-driven senescence associates with adverse clinical features in CSD patients. (**A**) Representative images of senescent cells in sham groups. (**B**) Representative images of HE and Masson staining of mouse uteri at 1 week after CS. Scale bars, 200 μm. (**C**) Representative images (left) and quantification (right) of p16-positive cells as a proportion of total cells in mouse CS uterine tissues in COX2/PGES inhibitors and AAV-sh*PTGES* treatment (*n* = 5 per group). Scale bars, 25 μm. (**D**) Representative IHC and IF images showing BrdU, Ki-67, and LaminB1 staining in human uterine tissues. Scale bars, 100 and 50 μm. (**E**) Quantification of H-scores for BrdU and Ki-67 proliferation markers and the frequency of LaminB1-positive cells in the total cells (*n* = 3–6 per group). (**F**) Representative mIF images showing colocalization of COX2/PGES and p21 in CSD TMAs. White arrows indicate colocalized cells. Scale bars, 200 and 50 μm. (**G**) Heatmap of Spearman correlations between colocalized expressions of COX2/PGES and p21 proteins by mIF and clinical characteristics of CSD patients. Clinical characteristics are detailed in Fig. EV1H. Color intensity indicates correlation coefficients. *P < 0.05. (**H**, **I**) Representative flow cytometry plots showing the isolation of human uterine macrophages, followed by PGE2 treatment, and quantification of p16 and Ki-67 expression by mean fluorescence intensity (MFI) (*n* = 5). (**J**) RT–qPCR of the relative mRNA expression of cellular senescent and SASP markers in human uterine macrophages following PGE2 treatment (*n* = 5). (**K**, **L**) Quantification of F4/80^+^ macrophages and vimentin^+^ utFBs within total p16-positive cells (**K**), and p16-positive cells within F4/80^+^ macrophages or vimentin^+^ utFBs (**L**) over time post-CS (*n* = 3–6 per group). At 1 week, *P* = 7.23 × 10⁻⁹ for macrophages versus uterine fibroblasts in CON mice and *P* = 1.19 × 10⁻¹² for the corresponding comparison in PGE2-treated mice. (**M**) Heatmap depicting relative mRNA expression levels of cellular senescence and SASP markers in THP1-derived macrophages, with color intensity indicating expression levels. *P < 0.05, **P < 0.01, ***P < 0.001. Data are mean ± s.d. One-way ANOVA with Tukey’s multiple comparisons test (**C**, **E**); Spearman correlations analysis (**G**); two-way ANOVA with Tukey’s multiple comparisons test (**K**, **L**); Unpaired *t* test (**I**, **J**, **M**). Independent biological samples were used; exact numbers of biological replicates are listed in each panel. In vitro cell experiments were conducted with biological triplicates or more. Source data are available online for this figure.

To substantiate the link between PGE2-mediated CSD formation and SnCs, we collected samples from CS mice over time: 1, 2, 3, 4, and 8 weeks post-CS. Immunofluorescence (IF) staining for the senescence marker p16 revealed a peak in SnCs occurrence at the earliest time point of 1 week post-CS. At this stage, excessive fibrosis was not yet evident at the CS sites (Figs. 3A,B and EV3A,B). The findings indicate that cellular senescence emerges as an early event in the process of fibrosis at the CS sites, which may be an initiating driver of fibrosis. In PGE2-treated mice, the number of SnCs remained elevated over time compared to the control group, leading to a significant accumulation of p16^+^ cells (Fig. 3A,B). Administration of COX2/PGES inhibitors or sh*PTGES* treatment significantly diminished the presence of p16^+^ cells at the CS site (Fig. EV3C). This reduction implies elevated PGE2 levels may promote the persistence of SnCs, thereby leading to uterine incision fibrosis and CSD symptoms.

To validate the clinical relevance of persistent SnCs, we examined fibrotic tissues from CSD patients, who exhibited significantly higher PGE2 levels compared with asymptomatic CS controls without scar defects. The IHC and RT–PCR analyses demonstrated significant upregulation of senescence and SASP markers, accompanied by reduced expression of proliferation markers and LaminB1 in CSD tissues (Figs. 3C,D and EV3D,E). These findings confirm the presence of persistent SnCs within fibrotic niches of CSD patients and support a conserved role for cellular senescence in human uterine niches formation.

Further analysis integrating TMAs with clinical data revealed that senescence and SASP markers expression positively correlated with imaging features of uterine niches in CSD patients. Elevated senescence burden was also associated with prolonged menstrual duration following CS and repair surgery and was negatively correlated with subsequent pregnancy outcomes after repair (Fig. 3E). Consistently, previous clinical studies have shown that classical SASP cytokines, including tumor necrosis factor-α (TNFα), interleukin-1β (IL-1β), and interleukin-6 (IL-6), are significantly enriched within uterine niches and are positively associated with poor fertility outcomes after repair surgery (Nobuta et al, 2022). These observations suggest that persistent SnCs and SASP amplification within fibrotic niches contribute to uterine dysfunction and symptom severity in CSD. Importantly, TMA-based analyses further demonstrated that colocalized expression of COX-2/PGES and the senescence marker p21 was positively correlated with uterine niche width and negatively correlated with TRM and post-repair pregnancy outcomes (Fig. EV3F,G). Collectively, these findings establish a strong link between COX-2/PGES/PGE2-mediated senescence persistence and both structural and functional deterioration in CSD, suggesting that targeting PGE2-driven senescence may represent a promising strategy to restore uterine integrity and reproductive function.

### PGE2 consecutively elicits macrophages and fibroblasts senescence

**Figure 4:**
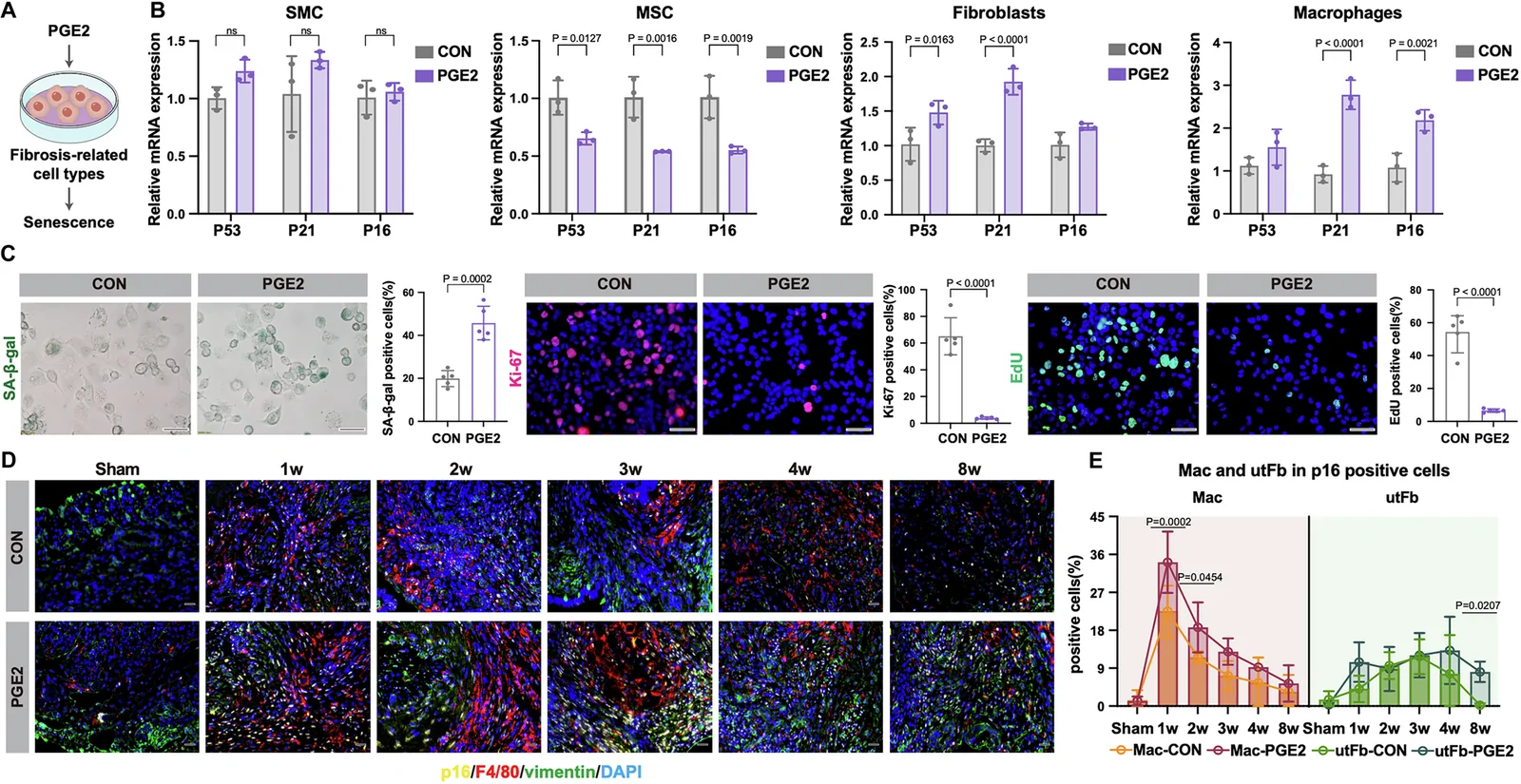
PGE2 sequentially promotes macrophages and fibroblasts senescence at uterine incision sites. (**A**) Scheme of PGE2-induced senescence phenotype in vitro. (**B**) Relative mRNA expression of cellular senescence markers in uterine smooth muscle cells (SMC), uterine fibroblasts, human umbilical cord mesenchymal stem cells (MSC), and THP1-derived macrophages. For P21 expression following PGE2 treatment, *P* = 5.73 × 10⁻⁵ in uterine fibroblasts and *P* = 1.89 × 10⁻⁵ in macrophages. (**C**) Representative images (left) of SA-β-gal, Ki-67, and EdU staining and quantification of staining-positive cells as a percentage of total cells (right) in THP1-derived macrophages. Scale bars, 50 μm. For CON vs PGE2, *P* = 9.61 ×  10⁻⁶ for Ki-67 positivity and *P* = 1.34 × 10⁻⁵ for EdU positivity. (**D**) Representative images of mIF indicating p16-positive cells at mouse CS sites over time post-CS. Scale bars, 20 μm. (**E**) Quantification of F4/80^+^ macrophages and vimentin^+^ utFBs within total p16-positive cells over time post-CS (*n* = 3–6 per group). Data are mean ± s.d. Unpaired *t* test (**C**); two-way ANOVA with Tukey’s or Šídák’s multiple comparisons test (**B**, **E**). Independent biological samples were used; exact numbers of biological replicates are listed in each panel. In vitro cell experiments were conducted with biological triplicates or more. Source data are available online for this figure.

To delineate the principal cell targets of PGE2-mediated senescence within fibrotic CSD tissues, we systematically evaluated the senescence response of multiple cell types known to participate in fibrosis (Qin et al, 2023; Layton et al, 2020; Laser et al, 2010). Human umbilical cord-derived mesenchymal stem cells (hUC-MSCs), uterine smooth muscle cells (utSMCs), uterine macrophages (utMACs), THP1-derived macrophages, and uterine fibroblasts (utFBs) were exposed to PGE2 in vitro (Fig. 4A). This approach enabled identification of the dominant senescent cell populations and provided insight into optimal cellular targets and timing for therapeutic intervention. Among all tested cell types, only macrophages (both utMACs and THP1-derived macrophages) and utFBs exhibited significant senescence-associated molecular features in response to PGE2 exposure (Figs. 4B,C and EV3H–J).

To further resolve the temporal dynamics of senescence in vivo, we performed sequential analyses of p16⁺ cell populations in CS mouse models. Compared with controls, PGE2-treated mice displayed a marked increase in p16⁺ senescent macrophages during the early phase (1–3 weeks) post-CS, whereas accumulation of p16⁺ senescent fibroblasts occurred predominantly during later stages (Figs. 4D and EV3K). These findings indicate that macrophages are among the earliest cell populations to undergo senescence in response to PGE2, while widespread fibroblast senescence likely arises as a secondary event. Notably, at one week post-CS, more than 40% of macrophages and over 30% of utFBs at incision sites in PGE2-treated mice exhibited senescence, as indicated by p16 expression (Fig. EV3L). This was significantly higher compared with the control group, consistent with in vitro results, highlighting the effect of PGE2 on cellular senescence in these cell populations. Together, although PGE2 is capable of inducing senescence in both macrophages and fibroblasts, macrophages constitute the dominant senescent population during the early post-CS phase (1–3 weeks), coinciding with the previously observed peak of senescent burden.

**Figure EV4:**
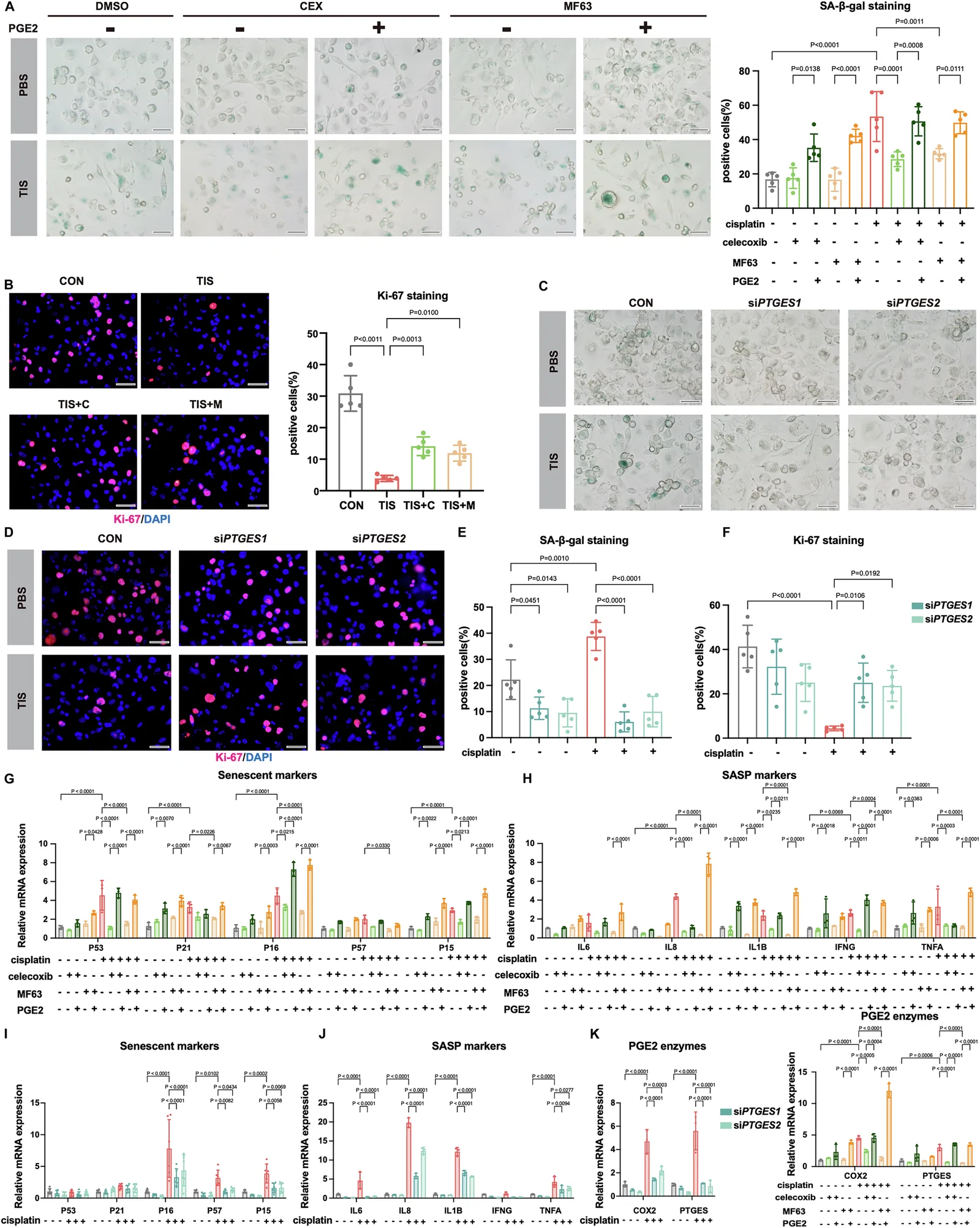
Targeting the COX2/PGES axis alleviates senescence and SASP in macrophages. (**A**–**F**) Representative images and quantification of SA-β-gal (**A**, **C**, **E**) and Ki-67 (**B**, **D**, **F**) in THP1-derived macrophages intervened with PGE2 inhibitors and *PTGES* knockdown. Scale bars, 50 μm. TIS therapy-induced senescence, C. celecoxib, M. MF63. (**G**–**K**) Analysis of relative mRNA expression for cellular senescence markers, SASP factors, and PGE2-associated enzymes in THP1-derived macrophages, intervened with PGE2 inhibitors and *PTGES* knockdown. Data are mean ± s.d. One-way ANOVA with Tukey’s multiple comparisons test (**A**, **B**, **E**, **F**). Two-way ANOVA with Tukey’s multiple comparisons test (**G**–**K**). Exact *P* values are provided for all comparisons, except values below 0.0001, which are reported as *P* < 0.0001 and uploaded in Source data. In vitro cell experiments were conducted with biological triplicates or more. Source data are available online for this figure.

While PGE2 is widely recognized as a component of SASP that amplifies age-related inflammation, its capacity to act as a primary inducer of senescence has not been fully resolved (Gonçalves et al, 2021; Minhas et al, 2021; Penke et al, 2020). Previous studies have demonstrated pro-senescent effects of PGE2 on fibroblasts in chronic obstructive pulmonary disease (Dagouassat et al, 2013). However, whether PGE2 promotes macrophage senescence has remained unclear. To address this, we conducted extensive in vitro analyses using THP1-derived macrophages, a robust and reproducible macrophage model that enables mechanistic interrogation of PGE2-induced responses. Given the limited abundance and susceptibility to fibroblast contamination of primary uterine macrophages during ex vivo culture, THP-1-derived macrophages were used for mechanistic studies. PGE2 treatment markedly increased senescence-associated β-galactosidase (SA-β-gal) activity and significantly reduced proliferation markers Ki-67 and EdU expression (Fig. 4C), consistent with suppressed immune cell proliferation, as previously reported in CD8⁺ T cells (Morotti et al, 2024; Lacher et al, 2024). RT–PCR analyses further revealed activation of p16/p21-associated pathways and upregulation of SASP factors, particularly *IL1B* and *IL8* (Figs. 4B and EV3M), indicating that PGE2 induces a senescent macrophage phenotype characterized by growth arrest and enhanced inflammatory cytokine secretion. Using a THP1 therapy-induced senescence (TIS) model, we next examined whether inhibition of the PGES/PGE2 axis could reverse macrophage senescence. Treatment with the COX-2/PGES inhibitors celecoxib and MF63 significantly reduced SA-β-gal staining and downregulated senescence and SASP markers, while restoring Ki-67 expression. Importantly, supplementation with exogenous PGE2 abrogated these protective effects (Fig. EV4A,B,G,H). Similar outcomes were observed following *PTGES* silencing using siRNA (Fig. EV4C–F,I,J), demonstrating that PGES/PGE2 signaling is a critical driver of macrophage senescence. Consistent with reports that PGD2-induced senescence activates prostaglandin-related pathways (Wiley et al, 2021), PGE2 further amplified COX-2/PGES signaling, promoting a feed-forward loop of senescence (Fig. EV4K). This heightened signaling enhanced SASP secretion, including PGE2 itself, thereby reinforcing senescence and inflammatory phenotypes in macrophages.

Collectively, expanding on our previous findings that SnCs can transmit aging characteristics (Yin et al, 2021), these findings support a model that PGE2-driven early macrophage senescence represents a pivotal upstream event that precedes and potentially transmits senescence signals to uterine fibroblasts, laying the foundation for subsequent fibroblast senescence and excessive fibrosis.

### Senescent macrophages drive fibroblast activation and secondary senescence through the IL-1β/IL-1R axis

**Figure 5:**
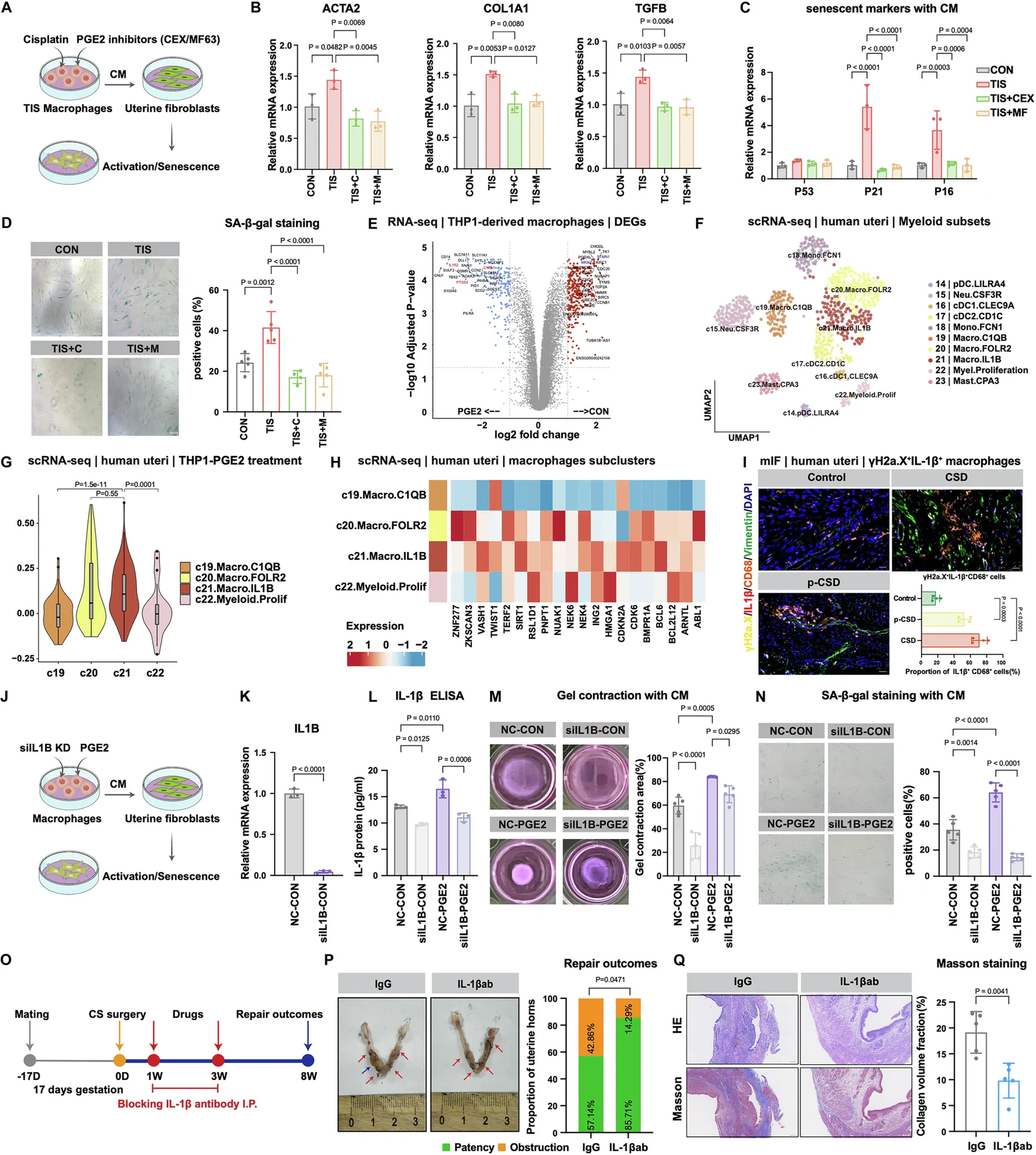
Senescent macrophages trigger uterine fibroblast activation and amplify aging via the IL-1β/IL1R axis. (**A**) Scheme of TIS in THP1-derived macrophages mediating utFBs activation and senescent phenotypes in vitro. (**B**) Relative mRNA expression levels of fibroblast activation markers in utFBs following treatment with CM from induced-senescent macrophages. TIS therapy-induced senescence, C. celecoxib, M. MF63. (**C**) Relative mRNA expression of cellular senescence markers in utFBs following treatment with CM from TIS macrophages. For P21, *P* = 1.71 × 10⁻⁷, 4.12 × 10⁻⁸, and 1.01 × 10⁻⁷ for CON vs TIS, TIS vs TIS + C, and TIS vs TIS + M, respectively. (**D**) Representative images of SA-β-gal staining (left) and frequency of positive staining cells among total cells (right) in utFBs after treatment with CM from TIS macrophages. Scale bars, 100 μm. *P* = 6.63 × 10⁻⁵ and 5.31 × 10⁻⁵ for TIS vs TIS + C and TIS vs TIS + M, respectively. (**E**) Volcano plot of DEGs in PGE2-treated THP1-derived macrophages. (**F**) UMAP for myeloid subclusters from uterine tissues of both healthy and CSD patients in scRNA-seq. (**G**) Expression patterns of the top 50 DEGs projected onto macrophage subclusters from scRNA-seq data. Violin plots show the distribution of expression values. In the embedded box plots, the center line indicates the median, the box bounds indicate the 25th and 75th percentiles, and the whiskers indicate the minimum and maximum values. (**H**) Heatmaps illustrating the expression of cellular senescence-related genes in macrophage subclusters. (**I**) Representative mIF images (left) and proportion of IL1β^+^ senescent macrophages (right) in CSD and healthy patients (*n* = 4 per group). Scale bars, 25 μm. For control vs CSD, *P* = 1.06 × 10⁻⁵. (**J**) Scheme of *IL1B* knockdown in senescent THP1-derived macrophages by PGE2 mediating utFBs activation and senescent phenotypes in vitro. (**K**) Relative mRNA expression levels of *IL1B* in macrophages. (**L**) ELISA quantification of IL-1β levels in CM from macrophages. (**M**, **N**) Representative images (left) and quantification (right) of gel contraction and SA-β-gal staining in utFBs after treatment with CM from *IL1B* knockdown macrophages with/without PGE2 treatment. Scale bars, 100 μm. For NC-CON vs siIL1B-CON, *P* = 1.15 × 10⁻⁵ (**M**); *P* = 5.02 × 10⁻⁶ for NC-CON vs NC-PGE2 and *P* = 2.47 × 10⁻⁹ for NC-PGE2 vs siIL1B-PGE2 (**N**). (**O**) Time scheme illustrating the establishment and analysis of mouse CS models with IL-1β blocking antibody treatment. (**P**) Representative gross morphological images of uteri and patency analysis in mouse CS models at 8 weeks post-CS in IL-1β blocking antibody treatment (*n* = 7 per group), with red arrows indicating CS suture endpoints and blue arrows marking obstructions. (**Q**) Representative images of HE and Masson staining (left) and quantification of collagen volume fraction (right) in IL-1β blocking antibody treatment (*n* = 5 per group). Scale bars, 100 μm. Data are mean ± s.d. One-way ANOVA with Tukey’s multiple comparisons test (**B**, **D**, **I**, **L**–**N**); two-way ANOVA with Tukey’s multiple comparisons test (**C**); unpaired *t* test (**K**, **Q**); Chi-squared test with one-tailed *P* value (**P**); DESeq2 with a two-sided Wald test and Benjamini–Hochberg correction for multiple testing (**E**); two-sided Wilcoxon rank-sum test (**G**). Independent biological samples were used; exact numbers of biological replicates are listed in each panel. In vitro cell experiments were conducted with biological triplicates or more. Source data are available online for this figure.

**Figure EV5:**
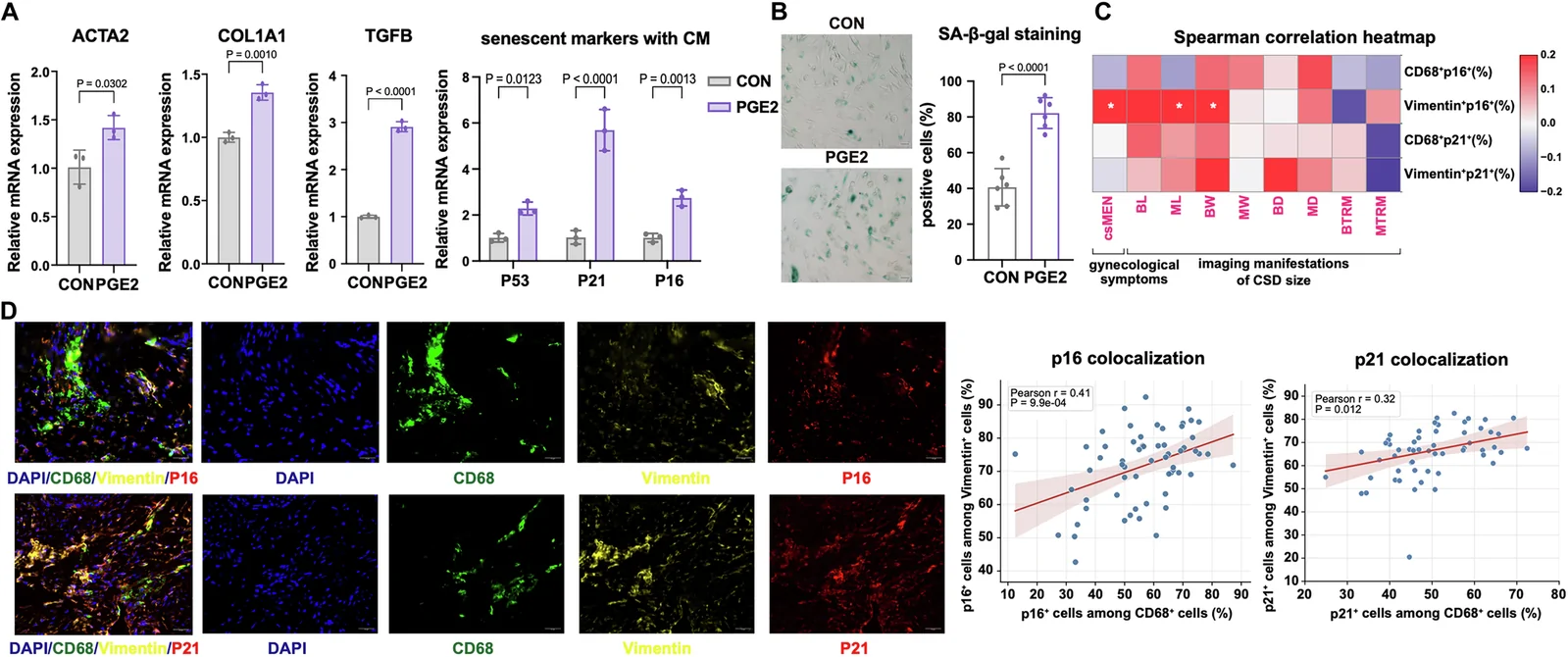
Senescent macrophages drive fibroblast senescence and fibrosis. (**A**) Relative mRNA expression levels of fibroblast activation and cellular senescence markers in utFBs following treatment with CM from PGE2-induced senescent macrophages. For CON versus PGE2, *P* = 7.26 × 10⁻⁶ for TGFB and P = 6.20 × 10⁻⁸ for P21. (**B**) Representative images of SA-β-gal staining (left) and frequency of positive staining cells among total cells (right) in utFBs after treatment with CM from PGE2-induced-senescent macrophages. Scale bars, 50 μm. For CON versus PGE2, *P* = 2.06 × 10⁻⁵. (**C**) Heatmap showing Spearman correlation coefficients between the colocalized expression of CD68/p16, CD68/p21, vimentin/p16, and vimentin/p21 detected by mIF and clinical characteristics of patients with CSD. Detailed clinical characteristics are provided in Fig. EV1H. Color intensity represents the magnitude of the correlation coefficient. *P < 0.05. (**D**) Representative mIF images of CSD TMAs (left) and correlation analysis of p16/p21-positive senescent macrophages and p16/p21-positive senescent fibroblasts (right) (*n* = 59-60). Scale bars, 25 μm. Data are mean ± s.d. unpaired *t* test (**A**, **B**); two-way ANOVA with Šídák’s multiple comparisons test (**A**); Spearman correlations analysis (**C**). Independent biological samples were used; exact numbers of biological replicates are listed in each panel. In vitro cell experiments were conducted with biological triplicates or more. Source data are available online for this figure.

During tissue repair, fibroblasts are transiently activated into myofibroblasts to facilitate extracellular matrix deposition and wound closure. However, excessive or sustained myofibroblast activation promotes cellular senescence, leading to pathological fibrosis and scar formation (Kato et al, 2020; Younesi et al, 2024). Having established that PGE2 preferentially induces early macrophage senescence and endows these cells with the capacity to transmit senescence-associated paracrine signals (Figs. 4C,D and EV4), we next sought to directly determine whether senescent macrophages drive fibroblast excessive activation and senescence. To this end, utFBs were cultured with conditioned medium (CM) derived from TIS macrophages or PGE2-treated THP1-derived macrophages (Figs. 5A and EV5A). Exposure to CM from senescent macrophages significantly increased the expression of fibroblast activation markers *ACTA2, COL1A1*, and *TGFB*, which was markedly attenuated when COX-2 or PGES inhibitors were applied (Figs. 5B and EV5A). In parallel, CM from TIS or PGE2-treated macrophages robustly enhanced SA-β-gal activity and increased expression of senescent markers in utFBs, effects that were similarly reversed by COX-2/PGES inhibition (Figs. 5C,D and EV5A,B). These findings indicate that soluble signals from PGE2-treated macrophages can promote fibroblast activation and senescence in vitro. To further investigate the clinical relevance of macrophage–fibroblast senescence coupling in CSD tissues, we performed mIF staining for p16/p21 together with CD68 (macrophages) and vimentin (fibroblasts) using another CSD tissue TMA cohort. Correlation analysis revealed that the abundance of vimentin^+^p16/p21^+^ senescent fibroblasts was positively correlated with CD68^+^p16/p21^+^ senescent macrophages. Furthermore, these cellular senescent signatures were positively associated with CSD size and inversely correlated with TRM (Fig. EV5C,D). These findings support a coordinated macrophage–fibroblast senescence program associated with defective tissue remodeling in CSD.

**Figure EV6:**
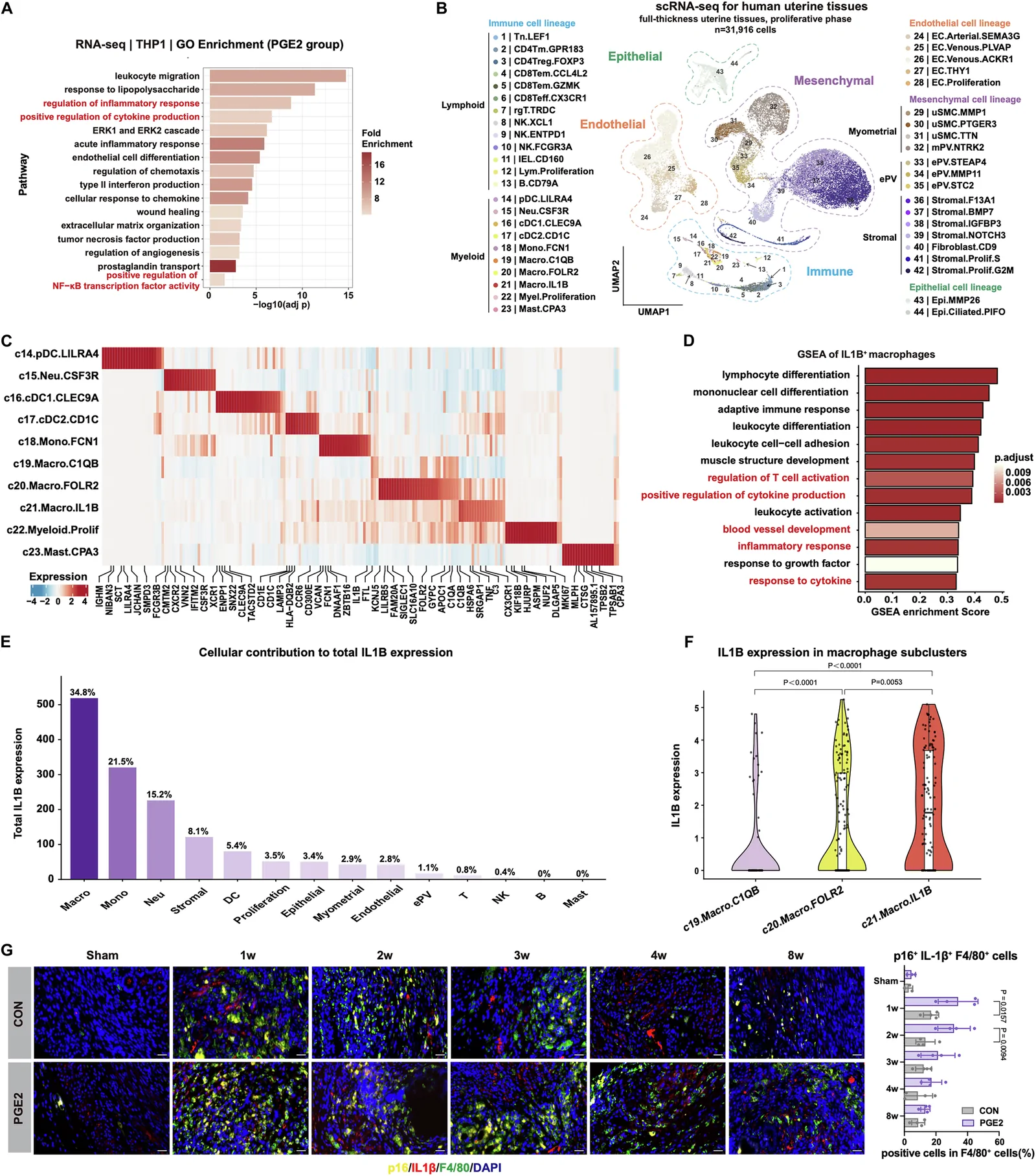
*IL1B*^+^ macrophages represent a pathogenic macrophage subset associated with impaired uterine repair. (**A**) GO enrichment pathway analysis on DEGs of THP1-derived macrophages treated with PGE2. (**B**) UMAP of scRNA-seq data from full-thickness CSD uterine tissues and healthy control uterine tissues (*n* = 3 per group). (**C**) Heatmap depicting scaled expression of marker genes for each myeloid subcluster in scRNA-seq. (**D**) Gene set enrichment analysis (GSEA) (GO biological processes) on DEGs of *IL1B*^+^ macrophages. (**E**) Bar plot showing the relative contribution of each cell type to total *IL1B* expression in scRNA-seq dataset. For each cell type, the contribution was calculated as the product of cell number and mean *IL1B* expression, divided by the summed products across all cell types. Percentages above the bars indicate the relative contribution of each cell type to total *IL1B* expression. (**F**) *IL1B* expression levels across macrophage subsets. Dots represent individual cells. In the embedded box plots, the center line indicates the median, the box bounds indicate the 25th and 75th percentiles, and the whiskers indicate the minimum and maximum values. c19 vs c20, p.adj 2.10 × 10⁻⁵; c19 vs c21p.adj 5.90 × 10⁻¹⁰. (**G**) Representative mIF images (left) and proportion of IL1β^+^ senescent macrophages (right) in over-time mouse CS models (*n* = 3–4 per group). Scale bars, 25 μm. Data are mean ± s.d. GO enrichment analysis was performed using a hypergeometric test and Benjamini–Hochberg correction for multiple testing (**A**, **D**); two-sided Wilcoxon rank-sum test (**F**); two-way ANOVA with Šídák’s multiple comparisons test (**G**). Independent biological samples were used; exact numbers of biological replicates are listed in each panel. Source data are available online for this figure.

To identify the key paracrine mediators released by senescent macrophages, we first performed bulk RNA sequencing on PGE2-treated THP1-derived macrophages. Differential gene expression analysis (DEGs) revealed significant enrichment of cytokine-related pathways, particularly NF-κB signaling (Figs. 5E and EV6A), suggesting that PGE2 induces a pro-inflammatory senescent macrophage phenotype. To further define the identity and functions of these macrophages in CSD patients, we conducted single-cell RNA sequencing (scRNA-seq) on unsorted full-thickness uterine tissues obtained from CSD patients undergoing vaginal repair (VR) surgery. To control for hormonal cycle-dependent variation, these data were integrated with scRNA-seq data of unsorted healthy proliferative-phase uterine full-thickness tissues reported by Wu et al (Wu et al, 2022). In the scRNA-seq, we analyzed 31,916 high-quality single cells (21,334 from CSD patients and 10,582 from healthy uterine tissues), categorizing them into four major cell lineages using canonical marker expressions (Fig. EV6B). Among them, three macrophage subsets were identified. *C1QB*⁺ macrophages exhibited complement-related gene expression, whereas *FOLR2*⁺ and *IL1B*⁺ macrophages resembled tissue-resident macrophages characterized by elevated *FOLR2* and *CD163* expression (Figs. 5F and EV6C). Mapping DEGs from PGE2-treated macrophages onto these defined subsets in scRNA-seq revealed enrichment of *IL1B*⁺ macrophages (Fig. 5G). Notably, among all macrophage subsets, *IL1B*⁺ macrophages displayed robust upregulation of senescence-associated genes (Fig. 5H). This population exhibited activation of pathways related to inflammatory responses, cytokine production, T cell activation, and angiogenesis (Fig. EV6D), all of which closely align with the histopathological features of CSD (Higuchi et al, 2022).

**Figure EV7:**
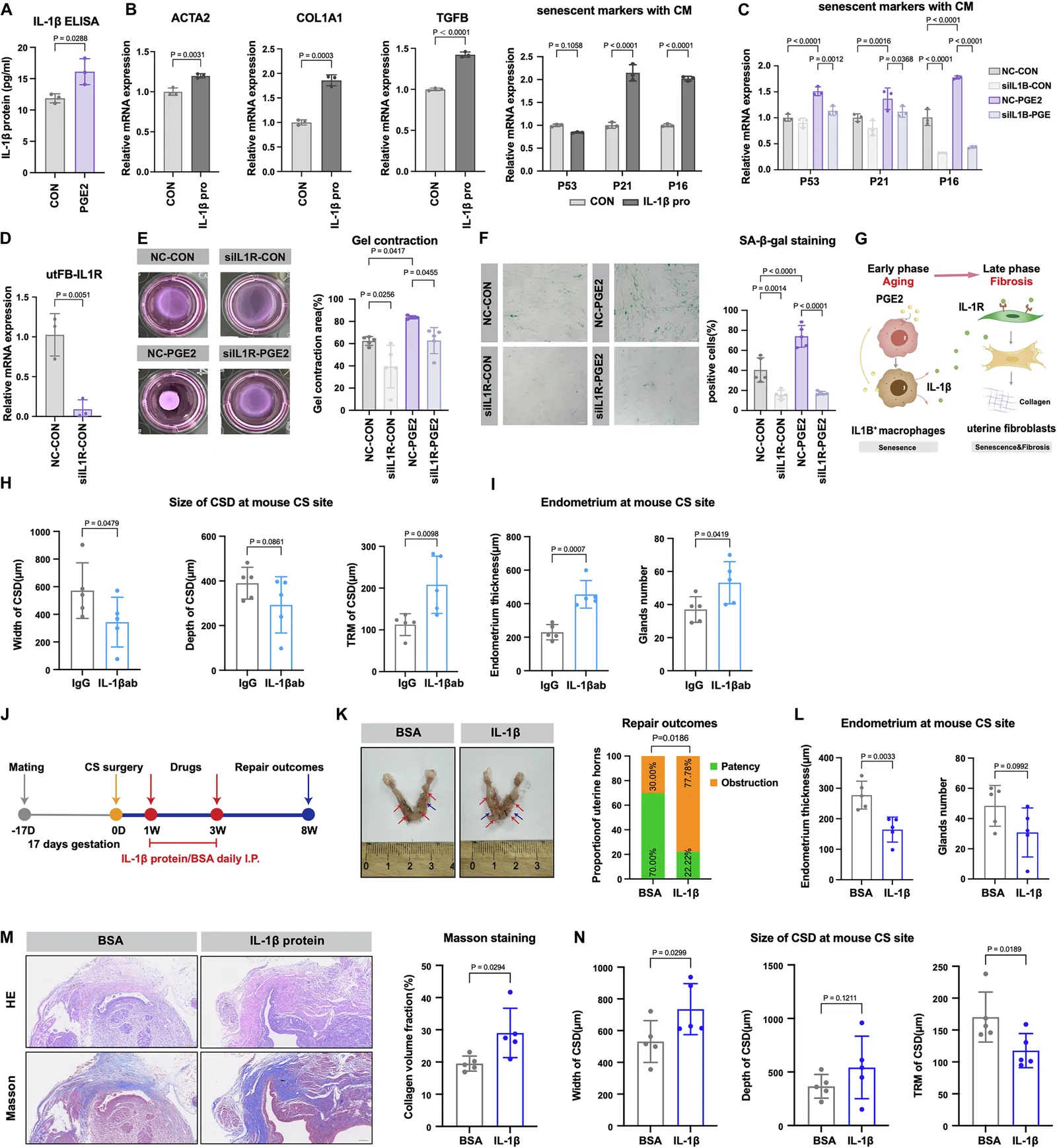
Blocking or supplementing IL-1β differentially regulates uterine incision fibrosis after CS. (**A**) ELISA quantification of IL-1β levels in CM from PGE2-induced senescence macrophages. (**B**) Relative mRNA expression levels of fibroblast activation and cellular senescence markers in utFBs after treatment with IL-1β protein. For CON versus IL-1β protein, *P* = 3.30 ×  10⁻⁵ for TGFB, *P* = 2.41 × 10⁻⁹ for P21, and *P* = 9.06 × 10⁻⁹ for P16. (**C**) Relative mRNA expression levels of cellular senescence markers in utFBs after treatment with CM from *IL1B* knockdown macrophages with/without PGE2 treatment. For P53, *P* = 2.43 × 10⁻⁵ for NC-CON versus NC-PGE2. For P16, *P* = 2.18 × 10⁻⁷, 2.53 × 10⁻⁸, and 2.87 × 10⁻¹³ for NC-CON versus siIL1B-CON, NC-CON versus NC-PGE2, and NC-PGE2 versus siIL1B-PGE2, respectively. (**D**) Relative mRNA expression levels of *IL1R* in utFBs after *IL1R* knockdown. (**E**, **F**) Representative images (left) and quantification (right) of gel contraction and SA-β-gal staining in *IL1R* knockdown utFBs after treatment with CM from PGE2-induced senescence macrophages. Scale bars, 100 μm. *P* = 5.99 × 10⁻⁵ for NC-CON versus NC-PGE2 and *P* = 7.05 × 10⁻⁸ for NC-PGE2 versus siIL1R-PGE2. (**G**) Scheme of *IL1B*^+^ macrophages transmitting senescence to induce uterine fibroblasts' senescence and fibrosis via IL-1β/IL1R axis. (**H**, **I**) Quantification of sizes of injury sites (**H**), endometrial thickness (**I**, left), and number of glands (**I**, right) at CS sites in mouse models at 8 weeks post-CS (*n* = 5 per group). (**J**) Time scheme illustrating the establishment and analysis of mouse CS models with IL-1β protein treatment. (**K**) Representative gross morphological images of uteri and patency analysis in mouse CS models at 8 weeks post-CS (*n* = 5 per group), with red arrows indicating CS suture endpoints and blue arrows marking obstructions. (**L**) Quantification of endometrial thickness (left), and number of glands (right) at CS sites in mouse models at 8 weeks post-CS (*n* = 5 per group). (**M**) Representative images of HE and Masson staining (left), and quantification of collagen volume fraction (right) at CS sites in mouse models at 8 weeks post-CS (*n* = 5 per group). Scale bars, 100 μm. (**N**) Quantification of sizes of injury sites at CS sites in mouse models at 8 weeks post-CS (*n* = 5 per group). Data are mean ± s.d. unpaired *t* test (**A**, **B**, **D**, **H**, **I**, **L**–**N**); two-way ANOVA with Tukey’s or Šídák’s multiple comparisons test (**B**, **C**); one-way ANOVA with Tukey’s multiple comparisons test (**E**, **F**); Chi-squared test with one-tailed *P* value (**K**). Independent biological samples were used; exact numbers of biological replicates are listed in each panel. In vitro cell experiments were conducted with biological triplicates or more. Source data are available online for this figure.

Single-cell transcriptomic analysis also revealed that *IL1B* expression was predominantly restricted to the myeloid compartment, with macrophages representing the major *IL1B*-expressing population compared with stromal cells, endothelial cells, and other immune cell subsets (Fig. EV6E–F). Multiplex immunofluorescence (mIF) staining further confirmed a pronounced increase in senescent γH2A.X⁺IL-1β⁺ macrophages in CSD tissues compared with controls, with these cells spatially co-localizing with fibroblasts (Fig. 5I). Consistently, temporal analysis in the mouse CS model demonstrated that p16⁺IL-1β⁺ senescent macrophages were highly enriched during the early phase of injury (Fig. EV6G), mirroring the findings observed in PGE2-treated macrophages. Given that IL-1β is a well-established pro-fibrotic SASP factor (Borthwick, 2016; Mitchell et al, 2023; Davis et al, 2020), we hypothesized that IL-1β mediates both fibroblast activation and senescence transmission. Indeed, ELISA confirmed elevated IL-1β secretion in CM from PGE2-treated macrophages (Fig. EV7A), and recombinant IL-1β protein alone was sufficient to induce excessive fibrotic activation and senescence phenotypes in utFBs (Fig. EV7B).

To functionally validate the IL-1β/IL-1R signaling axis, siRNA-mediated silencing of *IL1B* in macrophages significantly reduced IL-1β levels in CM and attenuated both fibrotic activation and senescence induction in utFBs during co-culture (Figs. 5J–N and EV7C). Conversely, knockdown of *IL1R* in utFBs markedly diminished their fibrotic and senescent responses to CM from PGE2-treated macrophages (Fig. EV7D–F). These results demonstrate that PGE2-induced senescent macrophages transmit pro-senescent signals to uterine fibroblasts predominantly through the IL-1β/IL-1R axis, and that disrupting this pathway effectively blocks the amplification of senescence and fibrosis (Fig. EV7G).

To further substantiate this mechanism in vivo, we administered an anti-IL-1β neutralizing antibody during the early post-injury phase (1–3 weeks) in CS mouse models. IL-1β blockade significantly attenuated fibrosis, reduced CSD size, and promoted endometrial repair at the incision site (Figs. 5O–Q and EV7H,I). In contrast, exogenous IL-1β protein administration during the same period markedly exacerbated fibrotic remodeling, increased defect size, and impaired endometrial repair (Fig. EV7J–N). Collectively, these pharmacological data are consistent with IL-1β signaling contributing to adverse uterine remodeling after CS.

### Senescent fibroblasts propagate pro-fibrotic signaling in uterine scar formation

**Figure EV8:**
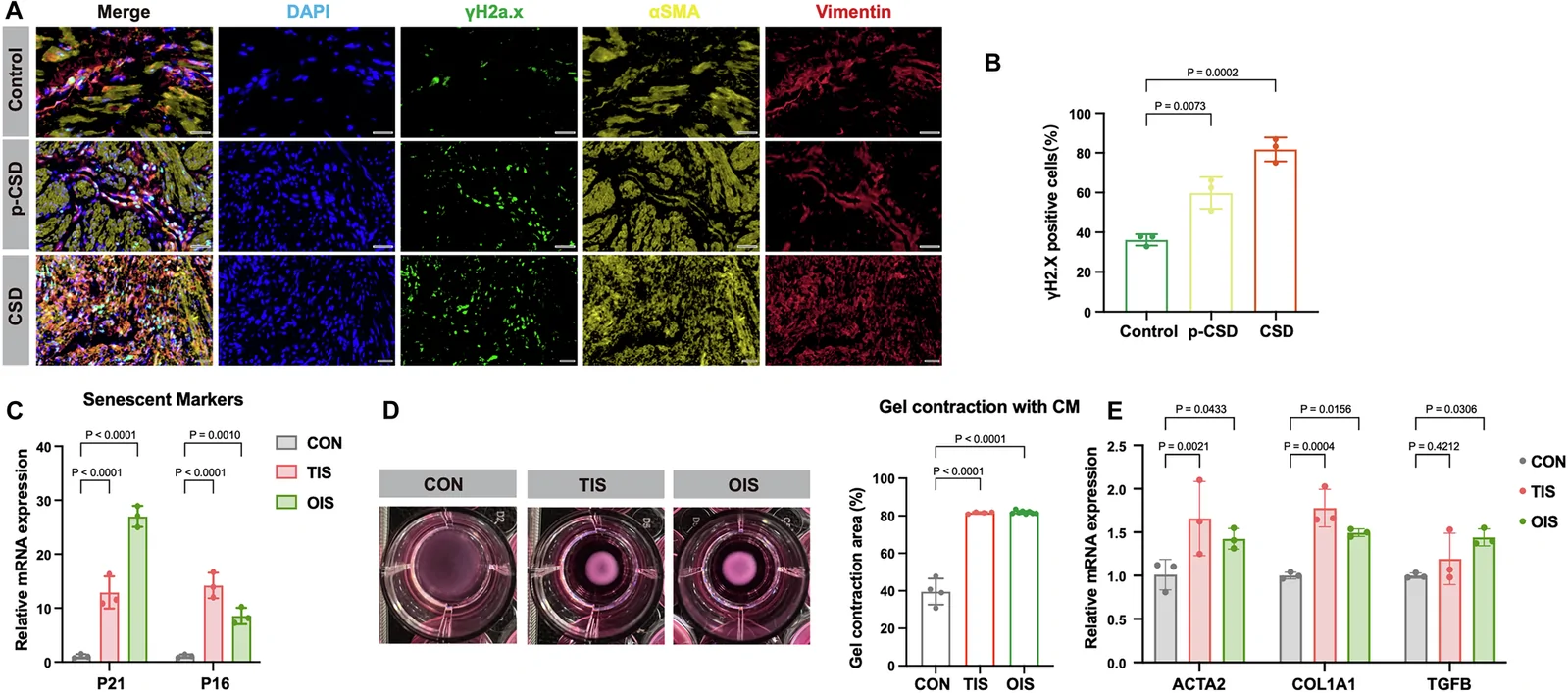
Senescent fibroblasts drive late-stage fibrotic niche formation. (**A**, **B**) Representative IF images (**A**) and frequency as a percentage of total cells (**B**) of γH2a.x expression in human uterine tissues (*n* = 3 per group). Scale bars, 25 μm. (**C**) Relative mRNA expression of cellular senescence markers in induced-senescence utFBs. OIS H_2_O_2_-induced senescence, TIS therapy-induced senescence. For P21, *P* = 1.36 × 10⁻⁵ and 2.47 × 10⁻⁹ for CON versus TIS and CON versus OIS, respectively; for P16, *P* = 4.76 × 10⁻⁶ for CON versus TIS. (**D**) Representative images (left) and quantification (right) of gel contraction in utFBs after treatment with CM from induced-senescence utFBs. *P* = 6.19 × 10⁻¹⁰ and 9.51 × 10⁻¹¹ for CON versus TIS and CON versus OIS, respectively. (**E**) Relative mRNA expression levels of fibrosis markers in utFBs after treatment with CM from induced-senescence utFBs. Data are mean ± s.d. One-way ANOVA with Tukey’s multiple comparisons test (**B**, **D**). Two-way ANOVA with Tukey’s multiple comparisons test (**C**, **E**). Biological replicates represent individual patients, with exact numbers listed in each panel. In vitro cell experiments were conducted with biological triplicates or more. Source data are available online for this figure.

Consistent with observations in the late phase of mouse CS models, we detected a substantial accumulation of senescent utFBs, including senescent activated fibroblasts, within fibrotic regions of CSD tissues (Fig. EV8A,B). This temporal concordance between fibroblast senescence and fibrosis progression suggests that the persistence of senescent fibroblasts represents a critical terminal event driving pathological fibrotic niche formation. To directly assess the fibrogenic capacity of senescent utFBs, we established two independent fibroblast senescence models: etoposide-induced TIS and H₂O₂-induced oxidative stress-induced senescence (OIS). In both models, senescent utFBs exhibited significantly enhanced gel contraction ability and marked upregulation of fibrotic markers (Fig. EV8C–E), demonstrating that fibroblast senescence alone is sufficient to promote fibrotic phenotypes. These findings indicate that senescent fibroblasts actively propagate pro-fibrotic cues, thereby reinforcing and perpetuating pathological tissue repair. Collectively, these results support a model in which CSD formation reflects a dynamic transition from an aging-dominated microenvironment to a fibrosis-dominated niche. Importantly, they further suggest that therapeutic strategies aimed at interrupting senescence accumulation or modulating the aging microenvironment may effectively halt or reverse fibrotic progression, providing a rationale for anti-aging strategies as an anti-fibrotic and potentially fertility-restorative approach in CSD.

### Therapeutic targeting of the aging microenvironment facilitates uterine repair

**Figure 6:**
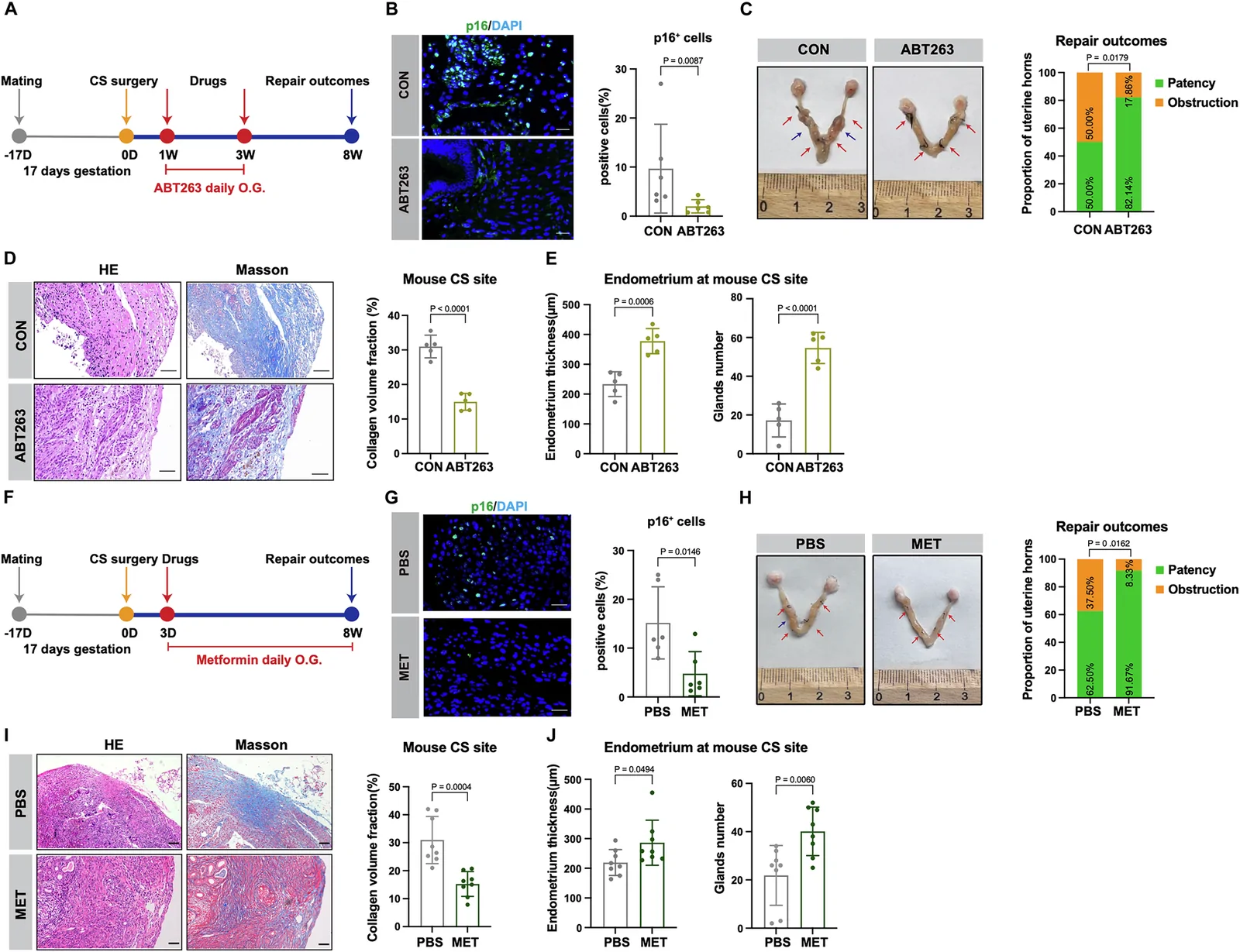
Anti-aging therapy mitigates cellular senescence and enhances uterine recovery post-CS. (**A**, **F**) Time scheme illustrating the establishment and analysis of mouse CS models with ABT263 or metformin treatment. (**B**, **G**) Representative mIF images (left) and proportion of senescent cells (right) at CS sites in mouse models at 8 weeks post-CS (*n* = 6 per group). Scale bars, 50 μm. (**C**, **H**) Representative gross morphological images of uteri and patency analysis in mouse CS models at 8 weeks post-CS (*n* = 10–14 per group), with red arrows indicating CS suture endpoints and blue arrows marking obstructions. (**D**, **I**) Representative images of HE and Masson staining (left) and quantification of collagen volume fraction (right) at CS sites in mouse models at 8 weeks post-CS (*n* = 5–8 per group). Scale bars, 50 μm. (**E**, **J**) Quantification of endometrial thickness (left), and number of glands (right) at CS sites in mouse models at 8 weeks post-CS (*n* = 5–8 per group). For gland number, *P* = 9.88 × 10⁻⁵ for CON vs ABT263. Data are mean ± s.d. Unpaired *t* test (**B**, **D**, **E**, **G**, **I**, **J**); Chi-squared test (**C**, **H**). Independent biological samples were used; exact numbers of biological replicates are listed in each panel. Source data are available online for this figure.

**Figure EV9:**
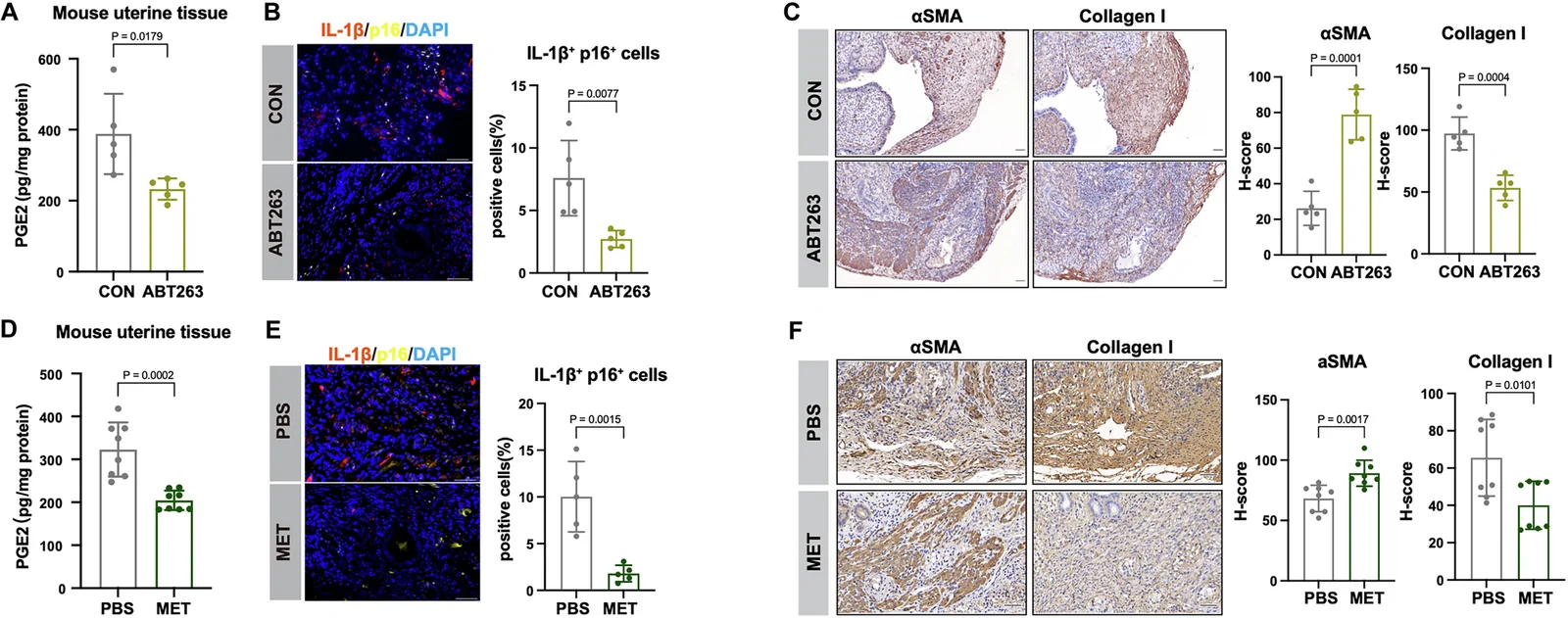
Targeting aging pathways promotes uterine incision repair after CS. (**A**, **D**) Quantification of PGE2 levels in mouse CS uterine tissues at 8 weeks post-CS by ELISA (*n* = 5–8 per group). (**B**, **E**) Representative images (left) and frequency of IL-1β^+^ senescent cells in total cells (right) in mouse CS uterine tissues at 8 weeks post-CS (*n* = 5 per group). Scale bars, 25 μm. (**C**, **F**) Representative IHC images of αSMA and collagen I expression (left) and quantification of H-score for αSMA and collagen I (right) at CS sites in mouse models at 8 weeks post-CS (*n* = 5–8 per group). Scale bars, 50 μm. Data are mean ± s.d. Unpaired *t* test (**A**–**F**). Biological replicates represent individual patients, with exact numbers listed in each panel. Source data are available online for this figure.

Given that CSD arises from early-stage macrophage senescence that propagates fibroblast activation and secondary senescence through the IL-1β/IL-1R axis, these findings indicate that fibrosis is a downstream consequence of a dysregulated aging microenvironment. We therefore reasoned that targeting the aging microenvironment, rather than individual signaling pathways, may also effectively disrupt the senescence–fibrosis circuit and promote coordinated uterine repair. Currently, two major therapeutic anti-aging strategies are widely recognized. Senolytic therapies selectively eliminate senescent cells (e.g., ABT263, dasatinib plus quercetin), whereas senomorphic approaches suppress SASP without inducing cell death, thereby attenuating aging-related dysfunction (e.g., metformin and mTOR inhibitors)(Di Micco et al, 2021; Yin et al, 2021). To evaluate whether eliminating senescent cells could alleviate excessive fibrosis following CS, we administered the senolytic agent ABT263 to mice during the peak phase of cellular senescence (1–3 weeks post-CS). ABT263 treatment markedly reduced the accumulation of senescent cells, including IL-1β⁺ senescent cells, decreased local PGE2 levels, and significantly improved uterine patency (Figs. 6A–C and EV9A,B). Histological analyses further demonstrated attenuated fibrotic deposition and enhanced restoration of the myometrial layer at the incision site (Figs. 6D and EV9C). Consistently, endometrial thickness and gland number were significantly increased following ABT263 treatment (Fig. 6E). These findings indicate that selectively targeting senescent cells effectively disrupts the PGE2-driven senescence–fibrosis cascade after CS.

To next assess whether modulating SASP signaling could similarly promote uterine repair, we administered the clinically approved senomorphic agent metformin to CS mice over an extended period (Fig. 6F). Metformin treatment significantly reduced PGE2 expression, decreased the abundance of senescent cells, improved uterine patency, and alleviated fibrosis (Figs. 6G–I and EV9D–F). In parallel, metformin markedly increased endometrial thickness and gland number (Fig. 6J), indicating coordinated recovery of both myometrium and endometrium. Collectively, these results demonstrate that therapeutic anti-aging strategies effectively mitigate PGE2-driven senescent cells accumulation and restore uterine structure and function following CS in preclinical models.

## Discussion

CSD is increasingly recognized as a complex uterine disorder that extends beyond the structural defect itself. To investigate whether metabolic alterations are associated with CSD, we performed metabolomic profiling of CSD and identified elevated PGE2 levels in CSD patients. Interestingly, formal comparisons did not reveal significant global metabolomic differences between paired CSD and para-CSD tissues, and PGE2 was similarly elevated in both regions. Together with the increase in serum PGE2, these findings suggest that CSD may involve a broader uterine inflammatory and metabolic microenvironment rather than an alteration confined to the anatomical defect. Circulating PGE2 may therefore warrant further evaluation as a candidate biomarker for CSD. In addition to PGE2, progesterone levels also showed a consistent downward trend in both CSD and para-CSD tissues, although its biological significance remains uncertain given physiological hormonal fluctuations and the lack of systematic hormonal measurements.

As a hormonally responsive organ with regenerative capacity, the uterus may be vulnerable to senescence-mediated impairment of incision healing after CS. In our scRNA-seq analysis and mIF validation, we also observed an increase in senescent *IL1B*^+^ macrophages in the defect tissues of CSD patients. Our experimental data further demonstrated that PGE2 contributes to macrophage senescence, and our bulk RNA-seq data suggest that this phenotype may involve PGE2-responsive inflammatory pathways, with NF-κB signaling providing a plausible link between PGE2 exposure and IL-1β induction. These findings are consistent with previous studies showing that PGE2 has been shown to induce the accumulation of *IL1B*^+^ macrophages (Caronni et al, 2023; Davis et al, 2020). Notably, the effects of PGE2 on cellular senescence and inflammation appear to be context- and cell type-dependent. In aging-related fibrotic diseases like chronic obstructive pulmonary disease, senescent lung fibroblasts release PGE2, increasing reactive oxygen species levels and upregulating p16 and p53. Activated p53 can further upregulate COX2 expression in receptor cells, thereby enhancing prostaglandin production and creating a positive feedback loop that amplifies PGE2 signaling (Gonçalves et al, 2021; Dagouassat et al, 2013). These findings suggest that PGE2 elevation may contribute to both the onset and amplification of senescence-associated inflammation. Conversely, in muscle stem cells, reduced PGE2 levels with increased PGE2-degrading enzyme 15-PGDH lead to mitochondrial dysfunction, cellular senescence, and subsequent loss of muscle strength and mass during sarcopenia (Palla et al, 2021; Prisk and Huard, 2003).

PGE2 is a well-established promoter of dysmenorrhea through the induction of myometrial and vascular contractions (Itani et al, 2022). CSD not only causes morphological changes at the uterine scar but also impairs endometrial function and is associated with increased incidences of dysmenorrhea and secondary infertility (Vissers et al, 2020). Persistent chronic inflammation, accompanied by the accumulation of inflammatory factors in the uterine cavity, has been proposed as a potential contributor to these CSD-associated functional abnormalities (Nobuta et al, 2022). Although our COX2/PGES intervention did not evaluate dysmenorrhea in mice, it improved several aspects of endometrial function, including increased endometrial thickness, gland number, and enhanced subsequent pregnancy rates. This discrepancy suggests that modulation of the COX2/PGES/PGE2 pathway may improve endometrial remodeling and reproductive function. Nevertheless, given that prolonged COX2 inhibition has been reported to cause pelvic muscle damage in pregnant rats, potentially leading to postpartum pelvic floor muscle dysfunction (Kobayashi et al, 2023), therapeutic strategies targeting the COX2/PGES/PGE2 signaling pathway or other anti-aging therapy post-CS must be carefully managed to effectively mitigate inflammaging and fibrosis while minimizing potential adverse effects.

Importantly, premature elimination of senescent cells may compromise wound repair(Rhinn et al, 2019), underscoring the need for precisely timed intervention. We found that senescent macrophages emerged as the predominant SnCs population at the incision site during the early post-CS period. Macrophage senescence has also been implicated in tumor microenvironments and endplate sclerosis, where it is associated with aging-related inflammation (Fan et al, 2024; Matacchione et al, 2022; Pence et al, 2022). During the late phase of wound healing, fibroblast senescence accompanied by persistent activation became more prominent, highlighting a temporally coordinated progression of senescence. This temporal pattern is consistent with the general sequence of wound healing, in which the macrophage-dominated inflammatory phase precedes fibroblast-driven tissue remodeling (Guo and DiPietro, 2010). Notably, macrophage-derived IL-1β may provide a mechanistic link between early macrophage senescence and fibroblast senescence during the later phase. However, the blockade of IL-1β did not fully reverse fibroblast responses, suggesting that additional factors may be involved in this process. Senescent fibroblasts are known to exhibit anti-apoptotic properties, contributing to the progression of fibrotic diseases (Shen et al, 2024). Although our mouse models differ and exhibit variations in CS site morphology, recent studies have similarly demonstrated that inhibiting aberrant fibroblast activation, such as through suppression of sFRP2 overexpression, promotes uterine wound healing (Cheng et al, 2025). Together, these findings indicate that senescent fibroblasts may serve as major downstream effectors that sustain extracellular matrix deposition and amplify the senescence microenvironment. Future lineage-tracing and cell type-specific depletion studies will be needed to define the contribution of fibroblasts in CSD, while broader functional analyses will be required to identify additional factors that mediate fibroblast senescence and further elucidate the underlying mechanisms.

Building on these findings, administration of the senolytic agent ABT263 and senomorphic agent metformin during the sub-inflammatory phase (1–3 weeks post-CS), corresponding to the period of peak SnC accumulation, significantly reduced fibrosis, promoted uterine incision repair, and improved uterine structure and function in mouse models. These findings indicate that limiting excessive senescence-associated signaling and preventing pathological SnCs persistence can facilitate effective uterine repair. However, metformin is pleiotropic, and additional senescence-independent mechanisms may also contribute, such as activating AMPK signaling, modulating mitochondrial homeostasis, and regulating oxidative stress, autophagy, and inflammatory responses (Foretz et al, 2019; Herzig and Shaw, 2018; Foretz et al, 2023). Collectively, our findings provide a framework for understanding CSD as a disorder of aberrant post-CS tissue remodeling and identify senescence-associated inflammation as a potentially actionable component of this pathological process.

## Methods

**Table:** Reagents and tools table

Reagent/resource	Reference or source	Identifier or catalog number
**Experimental models**		
Human CSD, paired para-CSD, and control myometrial tissues	Xinhua Hospital	Patient metadata: Dataset EV2
Primary human uterine macrophages	Xinhua Hospital	Patient metadata: Dataset EV2
C57BL/6 J mice, female	Shanghai Jihui Laboratory Animal Care Co., Ltd.	C57BL/6 J
THP-1 human monocytic cell line	Procell Life Science	Cat. no. CL-0233
Human uterine smooth muscle cells (utSMC)	Procell Life Science	Cat. no. CP-H053
Human uterine fibroblasts (utFB)	Procell Life Science	Cat. no. CP-H054
Human umbilical cord mesenchymal stem cells (hUC-MSC)	Procell Life Science	Cat. no. CP-CL11
**Recombinant DNA**		
AAV9 vector carrying shPTGES	GenePharma	5’-GCCAGAUGAGGCUGCGGAAGAtt-3’; 5’-UCUUCCGCAGCCUCAUCUGGCtt-3'
**Antibodies**		
Primary anti-COX2 antibody (Human; Multiplex immunofluorescence; 1:900)	Cell Signaling Technology	Cat. no. 12282
Primary anti-PGES antibody (Human; Mouse; Immunohistochemistry; 1:50; Human; Multiplex immunofluorescence; 1:150)	Santa Cruz Biotechnology	Cat. no. sc-166308
Primary anti-IL1β antibody (Human; Immunohistochemistry; 1:200)	Proteintech	Cat. no. 16806-1-AP
Primary anti-IL6 antibody (Human; Immunohistochemistry; 1:200)	Proteintech	Cat. no. 21865-1-AP
Primary anti-IL8 antibody (Human; Immunohistochemistry; 1:250)	Proteintech	Cat. no. 27095-1-AP
Primary anti-CCL2 antibody (Human; Immunohistochemistry; 1:200)	Servicebio	Cat. no. GB11199
Primary anti-TNFα antibody (Human; Immunohistochemistry; 1:200)	Cell Signaling Technology	Cat. no. 6945
Primary anti-IFNγ antibody (Human; Immunohistochemistry; 1:200)	Proteintech	Cat. no. 15365-1-AP
Primary anti-aSMA antibody (Mouse; Immunohistochemistry; 1:1500; Human; Multiplex immunofluorescence; 1:2000)	Proteintech	Cat. no. 14395-1-AP
Primary anti-collagen I antibody (Mouse; Immunohistochemistry; 1:2500; Human; Multiplex immunofluorescence; 1:2000)	Proteintech	Cat. no. 67288-1-Ig
Primary anti-Ki-67 antibody (Human; Immunohistochemistry; 1:500)	Servicebio	Cat. no. GB111499
Primary anti-Brdu antibody (Human; Immunohistochemistry; 1:200)	Servicebio	Cat. no. GB12051
Primary anti-β-galactosidase antibody (Human; Immunohistochemistry; 1:200)	Proteintech	Cat. no. 66586-1-Ig
Primary anti-P53 antibody (Human; Immunohistochemistry; 1:200)	Proteintech	Cat. no. 60283-2-Ig
Primary anti-P21 antibody (Human; Immunohistochemistry; 1:100; Human; Multiplex immunofluorescence; 1:50)	Cell Signaling Technology	Cat. no. 2947
Primary anti-P16 antibody (Human; Immunohistochemistry; 1:50; Human; Multiplex immunofluorescence; 1:100)	Abcam	Cat. no. ab270058
Primary anti-P16 antibody (Mouse; Immunofluorescence; 1:50; Mouse; Multiplex immunofluorescence; 1:200)	Abcam	Cat. no. ab211542
Primary anti-LaminB1 antibody (Human; Immunofluorescence; 1:200)	Proteintech	Cat. no. 12987-1-AP
Primary anti-IL1β antibody (Human; Multiplex immunofluorescence; 1:500)	Abcam	Cat. no. ab315084
Primary anti-vimentin antibody (Human; Multiplex immunofluorescence; 1:300; Mouse; Multiplex immunofluorescence; 1:750)	Abcam	Cat. no. ab8978
Primary anti-γH2a.X antibody (Human; Multiplex immunofluorescence; 1:100)	Abcam	Cat. no. ab81299
Primary anti-CD68 antibody (Human; Multiplex immunofluorescence; 1:5000)	Cell Signaling Technology	Cat. no. 76437
Primary anti-IL1β antibody (Mouse; Multiplex immunofluorescence; 1:200)	Cell Signaling Technology	Cat. no. 12242
Primary anti-F4/80 antibody (Mouse; Multiplex immunofluorescence; 1:500)	Cell Signaling Technology	Cat. no. 70076
Primary anti-vimentin antibody (Human; Multiplex immunofluorescence; 1:4000)	Proteintech	Cat. no. 60330-1-Ig
Primary anti-Ki-67 antibody (Human; Cellular immunofluorescence; 1:200)	Abcam	Cat. no. ab16667
Anti-human CD45-RB705 antibody (flow cytometry)	BD	clone HI30
Anti-human CD14-BV510 antibody (flow cytometry)	BioLegend	clone HCD14
Anti-human CD11b-PE-CY7 antibody (flow cytometry)	BioLegend	clone ICRF44
Anti-human CD68-APC antibody (flow cytometry)	BioLegend	clone Y1/82 A
Anti-human Ki-67-PE antibody (flow cytometry)	Miltenyi Biotec	clone REA183
Alexa Fluor 488-conjugated goat anti-rabbit IgG secondary antibody (flow cytometry; 1:1,000)	Abcam	Cat. no. ab150077
Anti-mouse/rat IL-1beta antibody (in vivo neutralization)	InVivoMAb	Cat. no. BE0246
Control Armenian hamster IgG (in vivo control)	InVivoMAb	Cat. no. BE0091
**Oligonucleotides and other sequence-based reagents**		
Human ACTB qPCR primer pair	This study	Forward: 5’-GGACTTCGAGCAAGAGATGG-3’; Reverse: 5’-AGGAAGGAAGGCTGGAAGAG-3'
Human AKR1C1 qPCR primer pair	This study	Forward: 5’-CATGCCTGTCCTGGGATTT-3’; Reverse: 5’-AGAATCAATATGGCGGAAGC-3'
Human AKR1C2 qPCR primer pair	This study	Forward: 5’-CTATGCGCCTGCAGAGGT-3’;Reverse: 5’-ACCTGCTCCTCATTATTGTAAACAT-3'
Human AKR1C3 qPCR primer pair	This study	Forward: 5’-CATTGGGGTGTCAAACTTCA-3’; Reverse: 5’-CCGGTTGAAATACGGATGAC-3'
Human ELOVL2 qPCR primer pair	This study	Forward: 5’-CACACTTCTCTCCGCGTACAT-3’; Reverse: 5’-GTTGTAGCCTCCTTCCCAAGT-3'
Human ELOVL4 qPCR primer pair	This study	Forward: 5’-CATTTGGCCCATGGATTC-3’; Reverse: 5’-CCAATGGTCACATGGAATTG-3'
Human ELOVL5 qPCR primer pair	This study	Forward: 5’-CCCTTCCATGCGTCCATA-3’; Reverse: 5’-GATTGTCAGCACAAACTGAAGC-3'
Human EPHX2 qPCR primer pair	This study	Forward: 5’-TTCTGCTGGACACCCTGAA-3’; Reverse: 5’-TTCAGATTAGCCCCGATGTC-3'
Human PTGDS qPCR primer pair	This study	Forward: 5’-CCAACTTCCAGCAGGACAAG-3’; Reverse: 5’-CCACAGACTTGCACATGGAC-3'
Human PTGES qPCR primer pair	This study	Forward: 5’-AGAGATGCCTGCCCACAG-3’; Reverse: 5’-TGGCCACCACGTACATCTT-3'
Human PTGES2 qPCR primer pair	This study	Forward: 5’-GTGACCGAGTTCGGCAATAAG-3’; Reverse: 5’-CGGACAATGTAGTCAAAGGACG-3'
Human PTGES3 qPCR primer pair	This study	Forward: 5’-TTAACAAAAGAAAGGGCAAAGC-3’; Reverse: 5’-TCAGAGAAACGATCAAAATTAGACAT-3'
Human PTGIS qPCR primer pair	This study	Forward: 5’-GATTTTTGATGTGCAGCTTCC-3’; Reverse: 5’-GTGAGTGCCTGGAGCTCTCT-3'
Human PTGR1 qPCR primer pair	This study	Forward: 5’-ATGATGGGGCAGCAAGTG-3’; Reverse: 5’-CCCATCAGAAATGGAGTGC-3'
Human PTGS1 qPCR primer pair	This study	Forward: 5’-TCCATGTTGGTGGACTATGG-3’; Reverse: 5’-GTGGTGGTCCATGTTCCTG-3'
Human PTGS2 qPCR primer pair	This study	Forward: 5’-GGTTGCTGGTGGTAGGAATGTTC-3’; Reverse: 5’-AAGACTGGTATTTCATCTGCCTGC-3'
Human TBXAS1 qPCR primer pair	This study	Forward: 5’-CTGCCCTATCTGGACATGGT-3’; Reverse: 5’-TGCCTCCCGTGTGAATCT-3'
Human P53 qPCR primer pair	This study	Forward: 5’-CAGCACATGACGGAGGTTGT-3’; Reverse: 5’-TCATCCAAATACTCCACACGC-3'
Human P21 qPCR primer pair	This study	Forward: 5’-AAGTCAGTTCCTTGTGGAGCC-3’; Reverse: 5’-GGTTCTGACGGACATCCCCA-3'
Human P16 qPCR primer pair	This study	Forward: 5’-GATTGAAAGAACCAGAGAGGCT-3’; Reverse: 5’-GACCTTCGGTGACTGATGAT-3'
Human P57 qPCR primer pair	This study	Forward: 5’-GGAAGGGGGTCACTCACAA-3’; Reverse: 5’-TACAGACGGCTCAGGAACCAT-3'
Human P15 qPCR primer pair	This study	Forward: 5’-ATGGCAAGTCACATGCGTAAA-3’; Reverse: 5’-TACCCCTCTGCTATCTGGTGG-3'
Human IL1B qPCR primer pair	This study	Forward: 5’-ATGATGGCTTATTACAGTGGCAA-3’; Reverse: 5’-GTCGGAGATTCGTAGCTGGA-3'
Human IL6 qPCR primer pair	This study	Forward: 5’-AACAACCTGAACCTTCCAAAG-3’; Reverse: 5’-CAAACTCCAAAAGACCAGTGA-3'
Human IL8 qPCR primer pair	This study	Forward: 5’-TTTTGCCAAGGAGTGCTAAAGA-3’; Reverse: 5’-AACCCTCTGCACCCAGTTTTC-3'
Human CCL2 qPCR primer pair	This study	Forward: 5’-CAGCCAGATGCAATCAATGCC-3’; Reverse: 5’-TGGAATCCTGAACCCACTTCT-3'
Human TNFA qPCR primer pair	This study	Forward: 5’-CCTCTCTCTAATCAGCCCTCTG-3’; Reverse: 5’-GAGGACCTGGGAGTAGATGAG-3'
Human IFNG qPCR primer pair	This study	Forward: 5’-TCGGTAACTGACTTGAATGTCCA-3’; Reverse: 5’-TCGCTTCCCTGTTTTAGCTGC-3'
Human ACTA2 qPCR primer pair	This study	Forward: 5’-CTATGCCTCTGGACGCACAACT-3’; Reverse: 5’-CAGATCCAGACGCATGATGGCA-3'
Human COL1A1 qPCR primer pair	This study	Forward: 5’-GTGCGATGACGTGATCTGTGA-3’; Reverse: 5’-CGGTGGTTTCTTGGTCGGT-3'
Human TGFB qPCR primer pair	This study	Forward: 5’-CAATTCCTGGCGATACCTCAG-3’; Reverse: 5’-GCACAACTCCGGTGACATCAA-3'
Human IL1R qPCR primer pair	This study	Forward: 5’-ATGAAATTGATGTTCGTCCCTGT-3’; Reverse: 5’-ACCACGCAATAGTAATGTCCTG-3'
siPTGES1 duplex	GenePharma	Sense: 5’-GCACGCUGCUGGUCAUCAAtt-3’; antisense: 5’-UUGAUGACCAGCAGCGUGCtt-3'
siPTGES2 duplex	GenePharma	Sense: 5’-CCGUGGCCUACCUGGGGAAtt-3’; antisense: 5’-UUCCCCAGGUAGGCCACGGtt-3'
siIL1B duplex	GenePharma	Sense: 5’-GAUGUCUGGUCCAUAUGAAtt-3’; antisense: 5’-UUCAUAUGGACCAGACAUCtt-3'
siIL1R duplex	GenePharma	Sense: 5’-CAGGAUUCAUCAACACAAAtt-3’; antisense: 5’-UUUGUGUUGAUGAAUCCUGtt-3'
**Chemicals, enzymes, and other reagents**		
Single Cell 3’ Library and Gel Bead Kit	10x Genomics	v3.1
TRIzol reagent for RNA-seq	Invitrogen	Cat. no. 15596018
TRIzol reagent for qRT-PCR	Takara	Cat. no. 9108
Prostaglandin E2 (PGE2)	Sigma	Cat. no. D2250000
Celecoxib	Absin	Cat. no. abs812001
CAY10526	MedChemExpress	Cat. no. HY-118119
Metformin	Sigma	Cat. no. 1396309
ABT263 (navitoclax)	MedChemExpress	Cat. no. HY-10087
Recombinant IL-1beta	PeproTech	Cat. no. 211-11B
PMA	MedChemExpress	Cat. no. HY-18739
Cisplatin	Selleck Chemicals	Cat. no. S1166
Etoposide	MedChemExpress	Cat. no. HY-13629
Hydrogen peroxide (H2O2)	Sigma	Cat. no. H1009
MF63	MedChemExpress	Cat. no. HY-13283
utSMC complete medium	Procell Life Science	Cat. no. CM-H053
utFB complete medium	Procell Life Science	Cat. no. CM-H054
hUC-MSC complete medium	Procell Life Science	Cat. no. CM-CL11
CD14 microbeads	Miltenyi Biotec	Cat. no. 130-050-201
Lipofectamine 2000	Thermofisher	Cat. no. 11668-019
SA-beta-galactosidase staining kit	Cell Signaling Technology	Cat. no. 9860
SA-beta-galactosidase staining kit	Beyotime	Cat. no. C0602
EdU staining kit	Beyotime	Cat. no. C0071
Foxp3/Transcription Factor Staining Set	Thermofisher	Cat. no. 00-5523-00
Rat-tail collagen type I solution	Corning	Cat. no. 354236
Immunostaining Blocking Solution	Beyotime	Cat. no. P0260
PrimeScript RT reagent kit	Takara	Cat. no. RR036A
SYBR Green qPCR reagent	Yeasen Biotechnology	Cat. no. 11202ES08
Prostaglandin E2 Parameter Assay kit	R&D Systems	Cat. no. KGE004B
**Software**		
Seurat	Seurat project	Version 5.1.0; v3 assay
clusterProfiler	Bioconductor	Version 4.12.6
limma	Bioconductor	Version 3.60.6
FlowJo	BD Biosciences	Version 10.9.0
ImageJ	National Institutes of Health	Version 1.54p
HALO Area Quantification algorithm	Indica Labs	HALO version 2.0
GraphPad Prism	GraphPad Software	Version 10.2.3
**Other**		
NovaSeq 6000 sequencing system	Illumina	NovaSeq 6000
NanoDrop ND-1000 spectrophotometer	Thermofisher	ND-1000
Agilent 2100 Bioanalyzer	Agilent Technologies	2100 Bioanalyzer
ExionLC AD system	SCIEX	ExionLC AD
QTRAP 6500+ mass spectrometer	SCIEX	QTRAP 6500+
BX53F microscope	Olympus	BX53F
Pannoramic MIDI slide scanner	3DHISTECH	Pannoramic MIDI
Confocal microscope	Leica	Leica TCS SP5

The collection of patient samples for this study was approved by the Ethics Committees of Xinhua Hospital (Approval No. XHEC-H-2018-002) and the Shanghai First Maternity and Infant Hospital, affiliated with Tongji University (Approval No. KS1512). Informed consent was obtained from all patients prior to the study. The study conformed to the principles set out in the WMA Declaration of Helsinki and the Department of Health and Human Services Belmont Report.

### Human samples collection

CSD was defined as a history of one or more CS, clinical symptoms of prolonged menstruation or intermenstrual bleeding after CS, and a uterine niche confirmed by transvaginal ultrasound (TVU) or magnetic resonance imaging (MRI). Exclusion criteria encompassed a history of endocrine disorders, pre-cesarean menstrual irregularities, coagulation disorders, use of intrauterine devices, or the presence of any other uterine pathology, such as submucosal fibroids, endometrial polyps, or hyperplasia or carcinoma. As controls, normal myometrial tissues were obtained from women under 40 years old undergoing surgery for uterine fibroids who had a history of CS but did not have CSD. Normal myometrial tissues were collected from areas more than 1 cm away from the lesion with no visible abnormalities. Detailed metadata for all sampled patients are presented in Dataset EV2. TMAs containing 118 or 60 paired CSD and para-CSD tissues from patients at Xinhua Hospital between 2015 and 2025 were produced. In addition, the TMA of the control group was made using 37 normal myometrial tissues from patients with benign gynecological diseases at Xinhua Hospital between 2020 and 2021.

### VR surgery

All VR surgeries were performed by a consistent surgical team. The surgical techniques previously detailed in our publication are briefly summarized here (Zhou et al, 2017). An initial anterior incision was made and positioned 0.5 cm below the reflexed vesicocervical junction. After entering the abdominal cavity and fully exposing the cervix, the precise location of the uterine defect was pinpointed. The extent of the niche in the lower uterine segment was assessed using the surgeon’s index finger in combination with an intrauterine sound. The CSD tissue was then excised, and the incision margins were trimmed to viable myometrium using dissecting scissors before closure.

### Follow-up

Prior to VR surgery, baseline demographic and clinical data were collected for CSD patients. This included age, CS history, uterine position, changes in menstrual patterns pre- and post-CS, and imaging data of uterine niches such as width, depth, length, and TRM as determined by TVU, MRI, and contrast-enhanced MRI (CE-MRI). All patients were scheduled for follow-up at 1, 3, and 6 months post-VR surgery. Follow-up assessments primarily focused on postoperative menstrual patterns and the imaging characteristics of uterine niches, with long-term follow-ups to determine the outcomes of subsequent pregnancies (details shown in Fig. EV1H).

### Single-cell RNA-sequence

CSD tissues were separated into endometrium and myometrium, cut into 1–2 mm³ pieces, and enzymatically dissociated in RPMI-1640 medium (Gibco) using gentleMACS (Miltenyi). The resulting suspensions were filtered through a 100-µm SmartStrainer, centrifuged at 400× *g* for 5 min, and treated with red blood cell lysis buffer (Miltenyi) on ice for 1–2 min. After washing with PBS (Gibco), cells were resuspended in sorting buffer (PBS + 2% FBS, Gibco) and stained with 7AAD for FACS sorting on a BD Melody. Viability and cell counts were assessed with a Countstar Automated Cell Counter. Endometrial and myometrial cells were then combined at a 1:1 ratio to generate single-cell suspensions for downstream analysis.

Single-cell capture and pre-amplification were performed on a GemCode instrument (10× Genomics) using the Single Cell 3′ Library and Gel Bead Kit v3.1, following the manufacturer’s instructions. Libraries were sequenced using an Illumina NovaSeq 6000 system. Cells with fewer than 200 expressed genes or fewer than 400 counts or over 20% mitochondrial UMIs were excluded. Gene expression matrices were normalized and scaled using the NormalizeData and ScaleData functions of the Seurat package (v.5.1.0) with the v3 assay version. Cells expressing markers for multiple major cell types were identified as doublets and removed. The remaining cells met the criteria for single-cell analysis. To integrate with data from healthy uterine tissues, CCA integration was performed using the FindIntegrationAnchors and IntegrateData functions (dim = 1:20) from Seurat. Dimensionality reduction was accomplished using UMAP via the RunUMAP function in Seurat, with principal components (PCs) (dims = 1:15) used for clustering being identical to those used for embedding calculations. Following the initial clustering to identify the major cell populations, we applied a second round of clustering. Specifically, the above Seurat pipeline was rerun with adjusted resolution tailored to the dataset characteristics using the top 10–15 PCs in each major cluster. Significantly overexpressed marker genes were identified using the FindAllMarkers function, defining cluster-specific signature genes with an adjusted *P* value < 0.05 via the Wilcoxon rank-sum test. These genes underwent GO enrichment analysis using the clusterProfiler package (v.4.12.6), considering GO terms with adjusted *P* values < 0.05 as significant.

### RNA-seq

Cell pellets were snap-frozen in liquid nitrogen and preserved in TRIzol (Invitrogen). Total RNA was extracted following the manufacturer’s instructions, and RNA quality was assessed using NanoDrop ND-1000 and Agilent Bioanalyzer 2100 (RIN > 7.0). Poly(A) RNA was enriched, fragmented, and reverse-transcribed into cDNA, followed by library preparation with adapter ligation and PCR amplification. Libraries with an average insert size of 300 ± 50 bp were sequenced on an Illumina NovaSeq 6000 platform (2 × 150 bp paired-end). Raw reads were processed with fastp to remove adapters and low-quality sequences. Clean reads were aligned to the human reference genome Homo sapiens GRCh38 using HISAT2, and transcript assembly was performed with StringTie. A merged transcriptome was generated using gffcompare, and transcript abundance was quantified as FPKM values. The RNA libraries were sequenced on the Illumina NovaseqTM 6000 platform by LC Bio Technology CO., Ltd (Hangzhou, China). The log2-transformed FPKM values were used for downstream analyses. Principal component analysis (PCA) was performed using the top 2000 most variable genes to visualize sample clustering. Differential expression analysis between groups was conducted using the LIMMA package (v3.60.6), and results were visualized with volcano plots. GO enrichment analysis was performed on significantly up- and downregulated genes using the clusterProfiler package (v4.12.6).

### Quasi-targeted metabolomics

Matched CSD tissues and para-CSD tissues were subjected to quasi-targeted metabolomics, along with normal myometrial tissues (*n* = 3). The study was conducted by Novogene Bioinformatics Technology Co., Ltd. in Beijing, China. Tissues were pulverized with liquid nitrogen, and the resulting homogenates were suspended in prechilled 80% methanol and centrifuged, preparing them for LC–MS/MS analyses. The LC–MS/MS analyses were carried out using an ExionLC™ AD system (SCIEX) paired with a QTRAP® 6500+ mass spectrometer (SCIEX). Metabolites were identified and quantified through multiple reaction monitoring (MRM) utilizing an in-house database. The data were processed using SCIEX OS software, which was strictly controlled to ensure accurate peak integration and noise reduction. Advanced bioinformatics analyses were performed using metaX software, enhancing our understanding of the metabolomic profiles and their implications.

### Targeted arachidonic acid metabolomics analysis

Paired uterine tissue samples from patients with CSD (*n* = 15), paired para-CSD tissues (*n* = 15), and healthy control myometrial tissues (*n* = 10) were subjected to targeted metabolomic profiling of arachidonic acid-related metabolites. Tissue samples were homogenized and processed for quantitative analysis using a targeted LC–MS/MS platform. A total of 42 metabolites were included in the targeted panel, covering key intermediates and downstream products involved in arachidonic acid metabolism, including prostaglandins, thromboxanes, hydroxyeicosatetraenoic acids (HETEs), epoxyeicosatrienoic acids (EETs), and related lipid mediators. In addition, progesterone and taurohyodeoxycholic acid, which were identified as enriched metabolites in healthy control tissues in the quasi-targeted metabolomic analysis, were incorporated into the targeted validation panel. Metabolite concentrations were normalized according to tissue weight, and differential metabolite abundance between CSD and healthy control tissues was assessed.

### Animal models and treatment

All animal procedures were conducted in accordance with guidelines approved by the Institutional Animal Care and Use Committee of the Model Animal Research Center at Xinhua Hospital, Shanghai Jiao Tong University School of Medicine (ethical approval number: XHEC-2019-KJB-005). All female C57BL/6J mice, aged 9 weeks, were acquired from Shanghai Jihui Laboratory Animal Care Co., Ltd. Throughout the experiments, all animals were provided free access to food and water and maintained under a standardized 12-h day–night light cycle.

To establish a mouse cesarean section model, female and male C57BL/6J mice of the same age were mated until the observation of a sperm plug indicated day 1 of gestation. On day 17 of gestation, the mice were anesthetized with an intraperitoneal injection of 0.3% pentobarbital sodium (0.1–0.2 ml/10 g). The surgery was performed under sterile conditions. A 1.5–2 cm abdominal incision was made, muscles and peritoneum were carefully separated, and the uterine horns were externalized. A 3-4 cm longitudinal incision was made along the midline above the uterine artery arch in the uterine horns. The fetuses and placenta were gently extracted through the hysterotomy. The hysterotomy was sutured continuously with 6-0 absorbable Prolene, and the edges of the uterine incision were marked with non-absorbable 5–0 Prolene to identify the site. The uterus was then returned to the abdominal cavity, which was rinsed with saline, and the peritoneum was closed with continuous 5-0 absorbable Prolene sutures. The skin was closed with interrupted 5-0 non-absorbable Prolene sutures. Three days of intraperitoneal penicillin were used after surgery to prevent infection. Interventions were carried out three days post-surgery to ensure the stability of the mice. Sham-operated mice underwent the same anesthesia, laparotomy, uterine-horn exposure, abdominal closure, and postoperative care as CS-operated mice, but no hysterotomy or uterine incision was performed. Sham animals received the corresponding vehicle treatments according to the same schedule as experimental controls.

Three days post-CS, mice received daily intraperitoneal injections of PGE2 in corn oil (500 μg/kg, Sigma, Cat no. D2250000), celecoxib in corn oil (10 mg/kg, Absin, Cat no. abs812001), and CAY10526 in corn oil (5 mg/kg, MCE, Cat no. HY-118119). In addition, daily oral administration of metformin (100 mg/kg, Sigma, Cat no. 1396309) in PBS was performed. Furthermore, from one to three weeks post-CS, ABT263 (MCE, Cat no. HY-10087) in a mixture of ethanol, PEG400, and Phosal 50 PG was administered via gavage at a dose of 50 mg/kg. Control groups received corresponding solvent treatments. During the same period, mice were intraperitoneally injected every 3 days with either anti-mouse/rat IL-1β antibody (InVivoMAb BE0246, 200 µg) or control Armenian hamster IgG (InVivoMAb BE0091, 200 µg). For IL-1β supplementation, mice were injected daily with 0.5 µg recombinant IL-1β (Peprotech, 211-11B) in 100 µl PBS containing 0.2% BSA, or vehicle (PBS + 0.2% BSA). To knock down *PTGES* expression in the murine uterus, shRNA against *PTGES* (5′- GCCAGAUGAGGCUGCGGAAGAtt-3′; 5′-UCUUCCGCAGCCUCAUCUGGCtt-3′) or a scrambled shRNA control carried by AAV9 (5 × 10^11^, GenePharma Biotechnology, Shanghai, China) was administered to mice via tail vein injection prior to mating. Mice were sacrificed at 1–4 and 8 weeks post-CS to observe the healing of the uterine incision. At 12 weeks post-CS, female mice were mated with proven fertile males to assess the function of the scarred uterus. The female mice were sacrificed 15 days after detection of vaginal plugs. The number, size, and implantation sites of fetuses in each horn were recorded for the fertility test.

### Uterine recovery assay

Eight weeks post-CS, mice were euthanized, and their uteri were fully excised and photographed to observe the incision sites. Each uterine horn was cut and subjected to methylene blue staining to assess patency rates. For a detailed examination of uterine incision repair, H&E staining was performed on paraffin-embedded sections of the uterine incision sites. Based on the H&E results, the width of the incision site was defined as the distance between the edges of the muscle layer defect; the depth was defined as the vertical distance from the line connecting the edges of the defect to the deepest part of the remaining tissue; and the TRM was defined as the thickness of the remaining fibrotic or muscular tissue connected to the depth. The endometrial thickness was determined by measuring the distance from the inner to the outer layer of the mouse endometrium, both horizontally and vertically, and then calculating the average of the four values. The number of glands at this level was also counted.

### Cell culture and treatment

Human macrophages were derived from THP1 cells (Procell, Cat no. CL-0233) treated with 100 nM PMA (MCE, Cat no. HY-18739) in RPMI-1640 containing 10% FBS and 100 U/ml penicillin-streptomycin for 48 h. Human uterine smooth muscle cells (utSMC) (Cat no. CP-H053), human uterine fibroblasts (utFB) (Cat no. CP-H054), and human umbilical cord mesenchymal stem cells (hUC-MSC) (Cat no. CP-CL11) were procured from Procell Life Science & Technology Co., Ltd. These cells were cultured in specialized complete medium: utSMC complete medium (Cat no. CM-H053), utFB complete medium (Cat no. CM-H054), and HucMSC complete medium (Cat no. CM-CL11). For primary human uterine macrophages, fresh human uterine tissues were enzymatically dissociated into single-cell suspensions using collagenase and DNase digestion. CD14^+^ myeloid cells were enriched by magnetic-activated cell sorting (MACS) using CD14 microbeads. The isolated cells were cultured in RPMI 1640 medium containing 10% fetal bovine serum and antibiotics. All primary cells were used within three passages.

Exogenous addition of 10 μM PGE2 (Sigma, Cat no. D2250000) for 48 h was used to assess proliferation and senescence in various cell types. To develop a therapy-induced senescence (TIS) model, 8 μM cisplatin (Selleck, Cat no. S1166) was applied to THP1-derived macrophages for 48 h. Treatment of utFB with 100 μM etoposide (MCE, Cat no. HY-13629) and 500 μM H_2_O_2_ (Sigma, Cat no. H1009) for 48 h was used to establish models for therapy-induced (TIS) and H_2_O_2_-induced senescence (OIS). After constructing the macrophage TIS model, 40 μM celecoxib (CXB) (Absin, Cat no. abs812001) and 10 μM MF63 (MF) (MCE, Cat no. HY-13283) were added for 48 h to evaluate the effects of COX2 and PGES inhibitors on senescence. Following the intervention, THP1 and utFB were cultured in FBS-free DMEM for 48 h, then the medium was centrifuged and filtered to prepare conditioned medium. This conditioned medium was mixed at a 1:1 ratio with complete utFB medium and added to 50% confluent utFB cells for subsequent functional assays.

### RNA interference

To generate THP1-induced macrophages with *PTGES* gene knockdown, si*PTGES*1 (sense 5′-GCACGCUGCUGGUCAUCAAtt-3′; antisense 5′-UUGAUGACCAGCAGCGUGCtt-3′) and si*PTGES*2 (sense 5′-CCGUGGCCUACCUGGGGAAtt-3′; antisense 5′-UUCCCCAGGUAGGCCACGGtt-3′) sequences; with *IL1B* gene knockdown, si*IL1B* (sense 5′- GAUGUCUGGUCCAUAUGAAtt-3′; antisense 5′-UUCAUAUGGACCAGACAUCtt-3′) and utFB with *IL1R* gene knockdown, si*IL1R* (sense 5′-CAGGAUUCAUCAACACAAAtt-3′; antisense 5′- UUUGUGUUGAUGAAUCCUGtt-3′) were designed and synthesized by GenePharma Biotechnology, Shanghai, China. Stealth siRNA negative control (siNC) duplexes with comparable GC content served as controls. The cells were transfected using Lipofectamine 2000 according to the manufacturer’s instructions.

### SA-β-gal and EdU staining

THP1 and utFB cells were prepared as described above and stained using colorimetric SA-β-galactosidase staining kits (Cell Signaling Technology, Cat no. 9860; Beyotime, Cat no. C0602) or EdU staining kit (Beyotime, Cat no. C0071) following the manufacturer’s instructions.

### Flow cytometric analysis (FACS)

Cells were stained with viability dye and surface antibodies to identify macrophages using the gating strategy of live singlets, CD45^+^CD14^+^CD11b^+^CD68^+^ cells. Surface staining was performed using anti-human CD45-RB705 (clone HI30), CD14-BV510 (clone HCD14), CD11b-PE-CY7 (clone ICRF44), and CD68-APC (clone Y1/82 A) antibodies. Following surface staining, cells were fixed and permeabilized using the Foxp3/Transcription Factor Staining Buffer Set (Thermo Fisher Scientific, Cat. no. 00-5523-00) according to the manufacturer’s instructions. Intracellular staining was performed using rabbit anti-human p16 antibody (Abcam, Cat. no. ab270058), followed by Alexa Fluor 488-conjugated goat anti-rabbit IgG secondary antibody (Abcam, Cat. no. ab150077). Ki-67 expression was detected using anti-human Ki-67-PE antibody (clone REA183, Miltenyi Biotec). Fluorescence intensity was acquired by flow cytometry, and p16 and Ki-67 expression levels were quantified as geometric mean fluorescence intensity (gMFI) within macrophages. Flow cytometry data were analyzed using FlowJo software.

### Gel contraction assay

The gel contraction assay was conducted using utFB cells prepared as described previously. The three-dimensional gels were formed by combining the cells with a neutralized rat tail collagen type 1 solution (3 mg/ml, pH 9, Corning, Cat no. 354236), achieving final concentrations of 1.0 mg/ml collagen and 3 × 10^4^ cells/ml matrix. This mixture was distributed into 24-well culture dishes (0.5 ml/well) pre-treated with PBS + 2% BSA. Digital images were captured after 96 h of treatment, and the average gel contraction ratio was quantified using imaging software.

### Tissue histochemistry and immunohistochemistry

Tissues were carefully dissected and immediately fixed in 4% paraformaldehyde at room temperature overnight. After fixation, tissues were embedded in paraffin, with human uterine tissues prepared as transverse cross-sections and mouse tissues as circular cross-sections of the uterine horn. Paraffin-embedded tissues were sectioned and then stained using H&E and Masson methods according to established protocols. Histological staining was performed by Shanghai Wo Deng and Wuhan Servicebio Biotechnology Co., Ltd. Slides containing human myometrium tissues and mouse uteri were deparaffinized and rehydrated. They were then incubated with various primary antibodies overnight at 4 °C, followed by incubation with secondary antibodies at room temperature for 1 h, a DAB chromogenic reaction, and counterstaining with hematoxylin. The stained samples were examined using a high-quality microscope (BX53F, Olympus), or scanned with a slice scanner (Pannoramic MIDI: 3Dhistech, Hungary) and the images were analyzed with ImageJ software using the H-score method.

### Tissue immunofluorescence

The fixation, embedding, sectioning, deparaffinization, and rehydration of uterine tissues from humans and mice were performed as previously described. Subsequent processing involved membrane permeabilization, antigen retrieval, and blocking of the slides from human and mouse tissues. For single-target immunofluorescence staining, the slides were incubated with primary antibodies overnight, followed by secondary antibodies for 1 h, and DAPI was utilized to stain cell nuclei. The stained samples were either examined using a confocal microscope or scanned with a slice scanner (Pannoramic MIDI: 3Dhistech, Hungary), and images were analyzed using ImageJ software. Protein expression was quantified as the mean value across three different sections.

mIF staining was applied to human and mouse uterine tissues, as well as to TMAs. Slides were sequentially incubated with primary antibodies, followed by signal amplification using a TSA kit (Wuhan Servicebio Technology Co., Ltd., China). Nuclei were counterstained with DAPI. The slides were scanned by the same slice scanner, and image analysis was performed using the HALO 2.0 Area Quantification algorithm (Indica Labs, Corrales, NM) at Zhenjiang Biosc Biotechnology Co., Ltd. (China).

### Cellular immunofluorescence

After various treatments, THP1 were fixed in 4% paraformaldehyde for 20 min. They were then blocked using QuickBlock™ Immunostaining Blocking Solution (Beyotime, Cat no. P0260) for 20 min at room temperature. Overnight incubation with primary antibodies was conducted at 4 °C. This was followed by a 1-h room temperature incubation with secondary antibodies and DAPI. The stained cells were imaged with a microscope (BX53F, Olympus), and the images were analyzed using ImageJ software.

### RNA isolation and quantitative real-time PCR (qRT-PCR)

Total RNA was extracted from tissues and cells utilizing TRIzol reagent (Takara, Cat no. 9108). This RNA was then reverse-transcribed into complementary DNA using the PrimeScript™ RT reagent kit (Takara, Cat no. RR036A), following the manufacturer’s protocols. qRT-PCR was conducted with SYBR green (Yeasen Biotechnology, Cat no. 11202ES08), and the expression levels were normalized to ACTB. The stability of ACTB expression was evaluated by comparing its raw Ct values across experimental groups with low variation. Data are expressed as means and standard deviations from three separate experiments. The primer sequences for the prostaglandin family were referenced from previously published articles (Wiley et al, 2021).

### ELISA

Human uterine tissues and serum, along with mouse uterine tissues, were rapidly frozen in liquid nitrogen and homogenized for the analysis of PGE2 expression levels. PGE2 levels were measured using the Prostaglandin E2 Parameter Assay kit (R&D Systems, KGE004B) following the manufacturer’s guidelines. The concentration of PGE2 in the samples was determined by comparing them to a standard curve prepared with known concentrations of PGE2.

### Statistical analyses

Statistical analyses were conducted using GraphPad Prism software, with results expressed as mean ± SD or as box plots, showing the median, interquartile range, and whiskers representing the minimum to maximum values. For normally distributed data, the unpaired Student’s *t* test was utilized for comparing two groups, while Welch’s correction was applied when variances were unequal. For data not following a normal distribution, significance was assessed using the Mann–Whitney test. Multiple group comparisons for normally distributed data were performed using one-way ANOVA; for non-normally distributed data, the Kruskal–Wallis test was used. For datasets involving two fixed factors, a two-way ANOVA was employed. Correlation analysis was done using either the Spearman or the Pearson methods. Statistical analysis used in each panel was described in the figure legends. Exact *P* values are provided for all comparisons, except values below 0.0001, which are reported as *P* < 0.0001.

## Supplementary information

Peer Review File

Dataset EV1

Dataset EV2

Figure EV5 Source Data

Source data Fig. 1

Source data Fig. 2

Source data Fig. 3

Source data Fig. 4

Source data Fig. 5

Source data Fig. 6

Figure EV1 Source Data

Figure EV2 Source Data

Figure EV3 Source Data

Figure EV4 Source Data

Figure EV6 Source Data

Figure EV7 Source Data

Figure EV8 Source Data

Figure EV9 Source Data

Expanded View Figures

## Data Availability

The scRNA-seq data of CSD and the bulk RNA-seq data of THP1-derived macrophages supporting the findings of this study are deposited at GSA-Human (HRA009739 and HRA013228). The source data of this paper are collected in the following database record: biostudies:S-SCDT-10_1038-S44321-026-00521-6.
