# Supplementary material for: Prostaglandin E2-mediated aging microenvironment fuels pathogenic fibrosis in cesarean section scar defect
Source: EMBO Mol Med. 2026 Oct 2;18(11):4300–33. doi: 10.1038/s44321-026-00521-6 (PMC13748614; doi:10.1038/s44321-026-00521-6)
Supplement: Supplementary file 1 [file 44321_2026_521_MOESM19_ESM.pdf]

Expanded View Figures

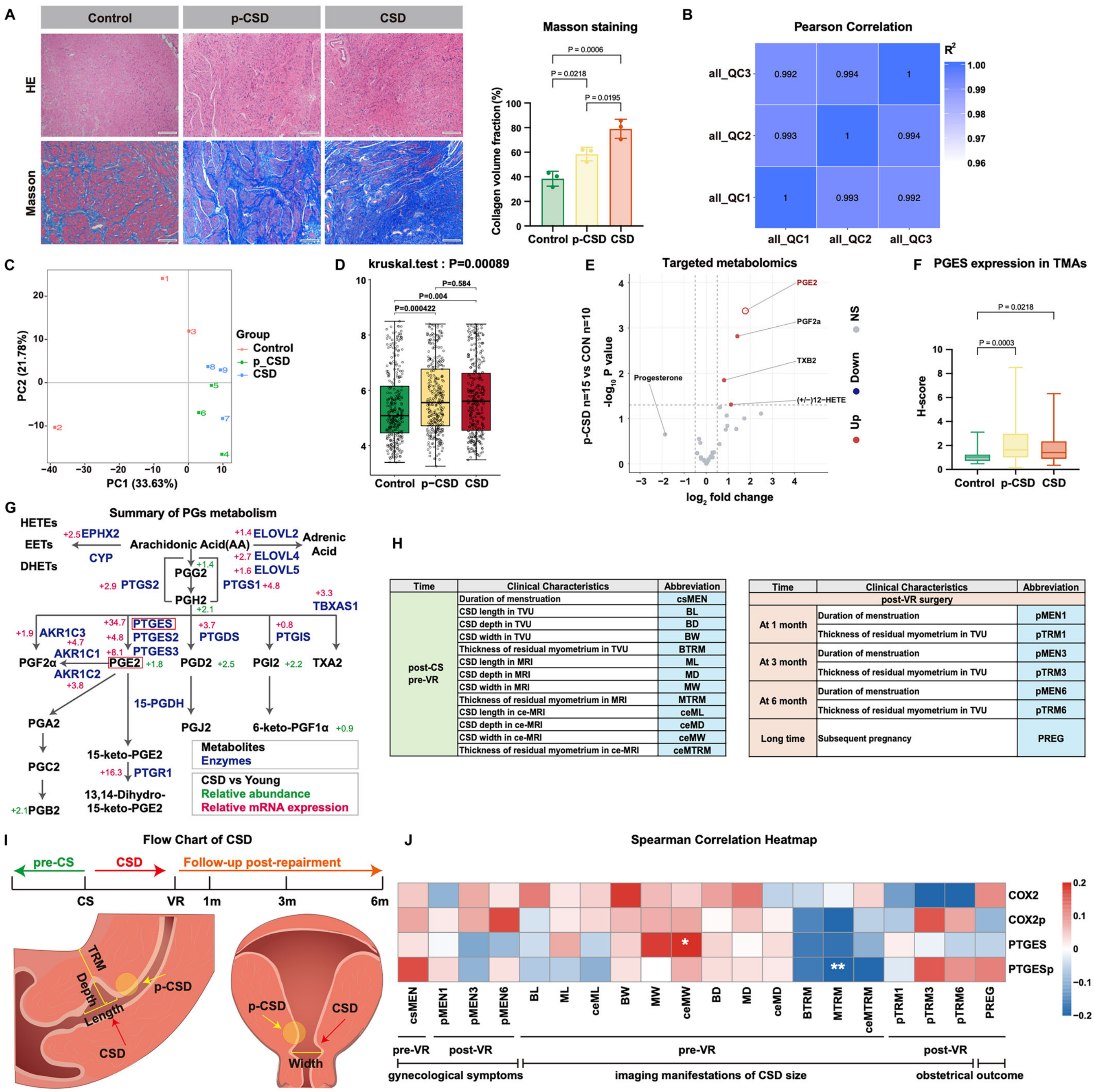

Time	Clinical Characteristics	Abbreviation
post-CS pre-VR	Duration of menstruation	csMEN
	CSD length in TVU	BL
	CSD depth in TVU	BD
	CSD width in TVU	BW
	Thickness of residual myometrium in TVU	BTRM
	CSD length in MRI	ML
	CSD depth in MRI	MD
	CSD width in MRI	MW
	Thickness of residual myometrium in MRI	MTRM
	CSD length in ce-MRI	ceML
	CSD depth in ce-MRI	ceMD
	CSD width in ce-MRI	ceMW
	Thickness of residual myometrium in ce-MRI	ceMTRM

Time	Clinical Characteristics	Abbreviation
post-VR surgery		
At 1 month	Duration of menstruation	pMEN1
	Thickness of residual myometrium in TVU	pTRM1
At 3 month	Duration of menstruation	pMEN3
	Thickness of residual myometrium in TVU	pTRM3
At 6 month	Duration of menstruation	pMEN6
	Thickness of residual myometrium in TVU	pTRM6
Long time	Subsequent pregnancy	PREG

Figure EV1. Correlation of PGES/PGE2 expression with adverse clinical features in CSD patients.

(**A**) Representative images of HE and Masson staining (left) and comparison of collagen volume fraction by Masson staining (right) among paired CSD and para-CSD tissues, healthy controls. Scale bars, 200 μm. ($n = 3$ per group). (**B**, **C**) Quality control and principal component analysis (PCA) of quasi-targeted metabolomics in human uterine tissues. (**D**) Statistical comparison of metabolite profiles among CSD, para-CSD tissues, and healthy controls ($n = 3$). (**E**) Volcano plot showing differential metabolites detected by targeted arachidonic acid metabolomics in para-CSD tissues ($n = 15$) and healthy control uterine tissues ($n = 10$). (**F**) H-score quantification of PGES protein expression in human myometrium TMAs ($n = 118$ for CSD and 37 for CON). (**G**) Prostaglandin metabolic pathway diagram highlighting key metabolites (black), enzymes (blue), relative abundance (green), and mRNA expression (pink) in CSD versus healthy controls. (**H**) Tables of clinical characteristics during the diagnosis and treatment of CSD. Left panel, baseline values before VR surgery; right panel, follow-up values after VR surgery. (**I**) Flowchart of clinical management from cesarean section to post-vaginal repair follow-up in CSD patients. (**J**) Heatmap of Spearman correlations between COX2/PGES protein expression in paired CSD and para-CSD TMAs and clinical features, with color intensity denoting correlation coefficients. *$P < 0.05$, **$P < 0.01$. Data are presented as box plots or mean ± s.d., showing the median, interquartile range, and whiskers representing the minimum to maximum values. One-way ANOVA with Tukey's multiple comparisons test (**A**, **F**); Welch's two-sample *t* test (**E**); Kruskal–Wallis test (**D**); Spearman's correlations analysis (**J**). Biological replicates represent individual patients, with exact numbers listed in each panel. Source data are available online for this figure.

 © The Author(s)

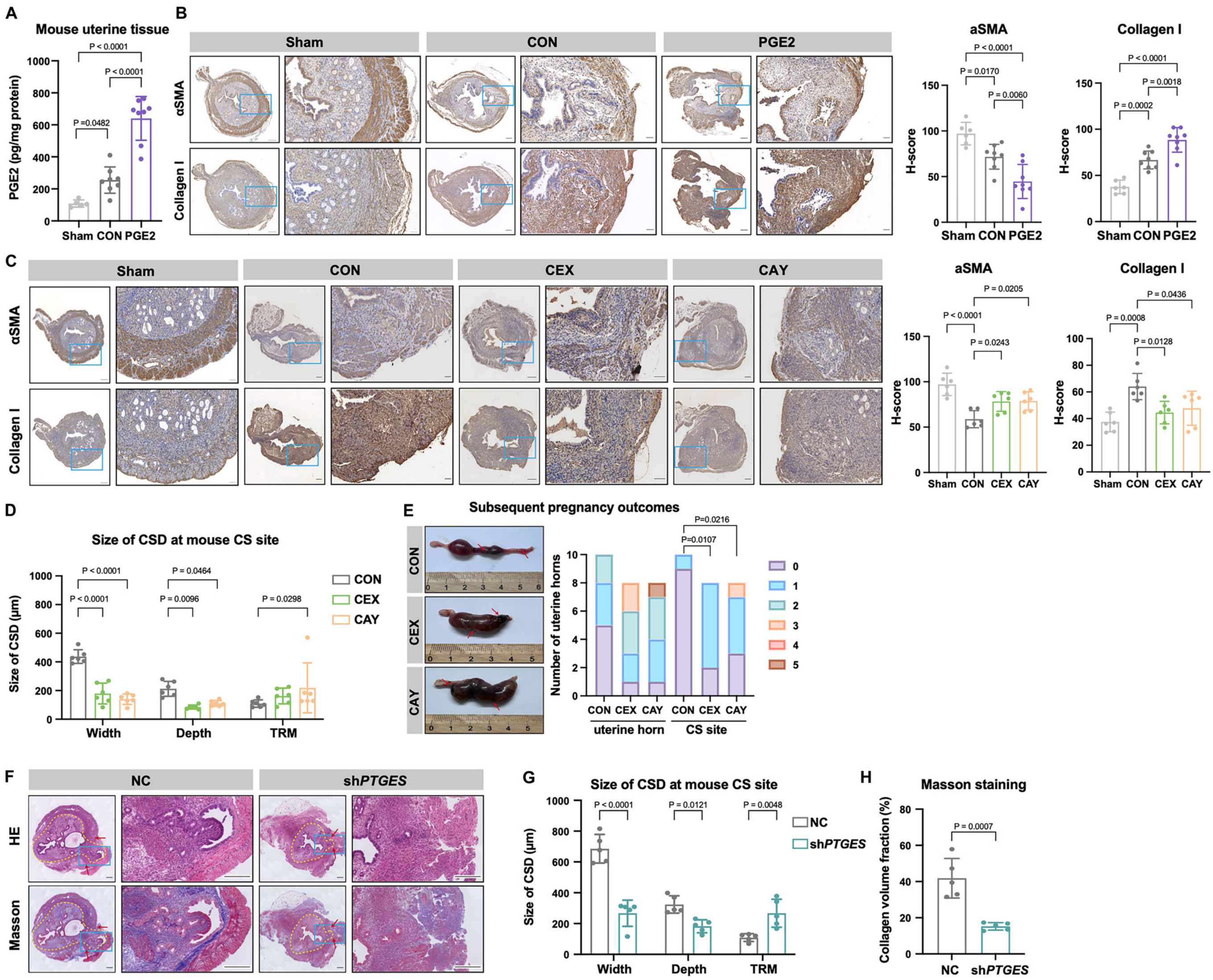

Figure EV2. Impacts of the COX2/PGES/PGE2 axis on uterine recovery post-CS.

(**A**) Quantification of PGE2 levels in uterine tissues from mouse CS models using ELISA at 8 weeks post-CS ($n = 5$–8 per group). $P = 1.21 \times 10^{-6}$ for CON versus PGE2 and $P = 7.50 \times 10^{-8}$ for Sham versus PGE2. (**B**) Representative IHC images of αSMA and collagen I at CS sites in PGE2-treated mice 8 weeks post-CS (left) with quantification shown via H-scores (right) ($n = 6$–8 per group). Scale bars, 200 and 50 µm. For Sham versus PGE2, $P = 1.29 \times 10^{-5}$ for αSMA and $P = 1.13 \times 10^{-7}$ for collagen I. (**C**) Representative IHC images (left) and H-score of αSMA and Collagen I expression (right) in COX2/PGES inhibitors-treated mouse CS uterine tissues at 8 weeks post-CS ($n = 6$ per group). Scale bars, 200 and 50 µm. (**D**) Quantification of sizes of CSD at CS sites 8 weeks post-CS ($n = 6$ per group). For CSD width, $P = 4.03 \times 10^{-7}$ and 1.57×10^{-8} for CON versus CEX and CON versus CAY, respectively. (**E**) Representative images of pregnant uteri (left) and quantification of fetuses in uterine horns and CS sites (right) ($n = 4$–5 per group). Red arrows indicate the location of the uterine incision. (**F**) Representative images of HE and Masson staining of *PTGES* knockdown in vivo at mouse CS sites at 8 weeks post-CS. Scale bars, 200 and 50 µm. Yellow circles demarcate the boundary between the endometrium and myometrium. Red arrows indicate the location of the uterine incision. (**G**) Quantification of sizes of CSD at CS sites 8 weeks post-CS ($n = 5$ per group). For CSD width, $P = 4.63 \times 10^{-9}$ for NC versus sh*PTGES*. (**H**) Quantification of collagen volume fraction at CS sites at 8 weeks post-CS ($n = 5$ per group). Data are mean ± s.d. One-way ANOVA with Tukey's multiple comparisons test (**A–C**); two-way ANOVA with Tukey's or Šídák's multiple comparisons test (**D**, **G**); unpaired *t* test (**H**), Kruskal–Wallis test (**E**). Independent biological samples were used; exact numbers of biological replicates are listed in each panel. Source data are available online for this figure.

© The Author(s)

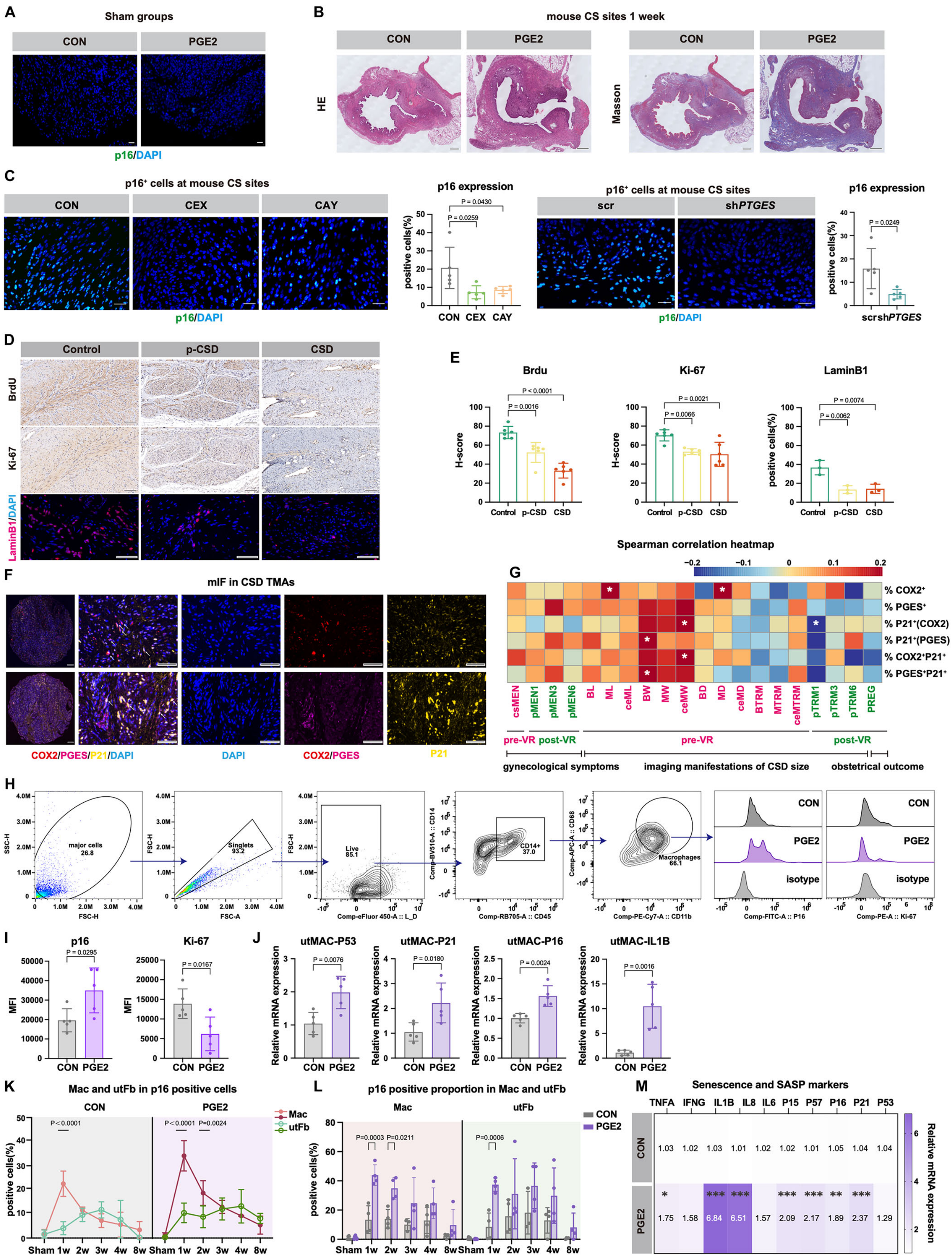
A
Sham groups
CON
PGE2
p16/DAPI
B
mouse CS sites 1 week
HE
Masson
C
p16⁺ cells at mouse CS sites
CEX
CAY
p16 expression
scr
shPTGES
D
Control
p-CSD
CSD
BrdU
Ki-67
LaminB1/DAPI
E
Brdu
LaminB1
F
mIF in CSD TMAs
COX2/PGES/P21/DAPI
G
Spearman correlation heatmap
gynecological symptoms
imaging manifestations of CSD size
obstetrical outcome
H
I
J
utMAC-P53
utMAC-P21
utMAC-P16
utMAC-IL1B
K
Mac and utFb in p16 positive cells
L
p16 positive proportion in Mac and utFb
M
Senescence and SASP markers

Figure EV3. COX2/PGES axis-driven senescence associates with adverse clinical features in CSD patients.

(**A**) Representative images of senescent cells in sham groups. (**B**) Representative images of HE and Masson staining of mouse uteri at 1 week after CS. Scale bars, 200 µm. (**C**) Representative images (left) and quantification (right) of p16-positive cells as a proportion of total cells in mouse CS uterine tissues in COX2/PGES inhibitors and AAV-sh*PTGES* treatment ($n = 5$ per group). Scale bars, 25 µm. (**D**) Representative IHC and IF images showing BrdU, Ki-67, and LaminB1 staining in human uterine tissues. Scale bars, 100 and 50 µm. (**E**) Quantification of H-scores for BrdU and Ki-67 proliferation markers and the frequency of LaminB1-positive cells in the total cells ($n = 3$–6 per group). (**F**) Representative mIF images showing colocalization of COX2/PGES and p21 in CSD TMAs. White arrows indicate colocalized cells. Scale bars, 200 and 50 µm. (**G**) Heatmap of Spearman correlations between colocalized expressions of COX2/PGES and p21 proteins by mIF and clinical characteristics of CSD patients. Clinical characteristics are detailed in Fig. EV1H. Color intensity indicates correlation coefficients. *$P < 0.05$. (**H**, **I**) Representative flow cytometry plots showing the isolation of human uterine macrophages, followed by PGE2 treatment, and quantification of p16 and Ki-67 expression by mean fluorescence intensity (MFI) ($n = 5$). (**J**) RT–qPCR of the relative mRNA expression of cellular senescent and SASP markers in human uterine macrophages following PGE2 treatment ($n = 5$). (**K**, **L**) Quantification of F4/80$^+$ macrophages and vimentin$^+$ utFBs within total p16-positive cells (**K**), and p16-positive cells within F4/80$^+$ macrophages or vimentin$^+$ utFBs (**L**) over time post-CS ($n = 3$–6 per group). At 1 week, $P = 7.23 \times 10^{-9}$ for macrophages versus uterine fibroblasts in CON mice and $P = 1.19 \times 10^{-12}$ for the corresponding comparison in PGE2-treated mice. (**M**) Heatmap depicting relative mRNA expression levels of cellular senescence and SASP markers in THP1-derived macrophages, with color intensity indicating expression levels. *$P < 0.05$, **$P < 0.01$, ***$P < 0.001$. Data are mean ± s.d. One-way ANOVA with Tukey's multiple comparisons test (**C**, **E**); Spearman correlations analysis (**G**); two-way ANOVA with Tukey's multiple comparisons test (**K**, **L**); Unpaired *t* test (**I**, **J**, **M**). Independent biological samples were used; exact numbers of biological replicates are listed in each panel. In vitro cell experiments were conducted with biological triplicates or more. Source data are available online for this figure.

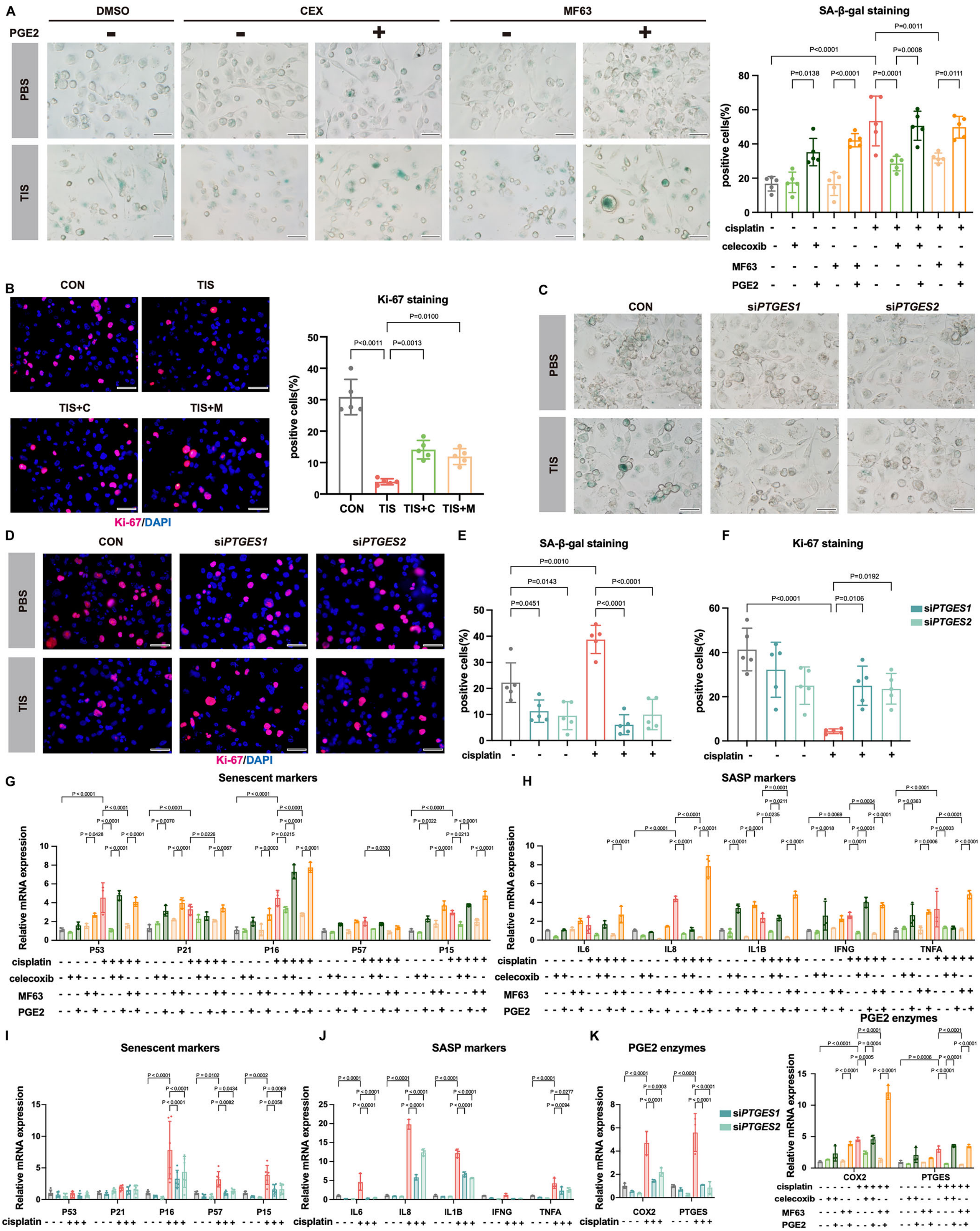
A
DMSO
CEX
MF63
PGE2
PBS
TIS
SA-β-gal staining
positive cells(%)
cisplatin
celecoxib
MF63
PGE2
B
CON
TIS
TIS+C
TIS+M
Ki-67/DAPI
Ki-67 staining
C
siPTGES1
siPTGES2
D
E
F
G
Senescent markers
Relative mRNA expression
P53
P21
P16
P57
P15
H
SASP markers
IL6
IL8
IL1B
IFNG
TNFA
I
J
K
PGE2 enzymes
COX2
PTGES

Figure EV4. Targeting the COX2/PGES axis alleviates senescence and SASP in macrophages.

(A–F) Representative images and quantification of SA-β-gal (**A**, **C**, **E**) and Ki-67 (**B**, **D**, **F**) in THP1-derived macrophages intervened with PGE2 inhibitors and *PTGES* knockdown. Scale bars, 50 µm. TIS therapy-induced senescence, C. celecoxib, M. MF63. (**G–K**) Analysis of relative mRNA expression for cellular senescence markers, SASP factors, and PGE2-associated enzymes in THP1-derived macrophages, intervened with PGE2 inhibitors and *PTGES* knockdown. Data are mean ± s.d. One-way ANOVA with Tukey's multiple comparisons test (**A**, **B**, **E**, **F**). Two-way ANOVA with Tukey's multiple comparisons test (**G–K**). Exact *P* values are provided for all comparisons, except values below 0.0001, which are reported as $P < 0.0001$ and uploaded in Source data. In vitro cell experiments were conducted with biological triplicates or more. Source data are available online for this figure.

© The Author(s)

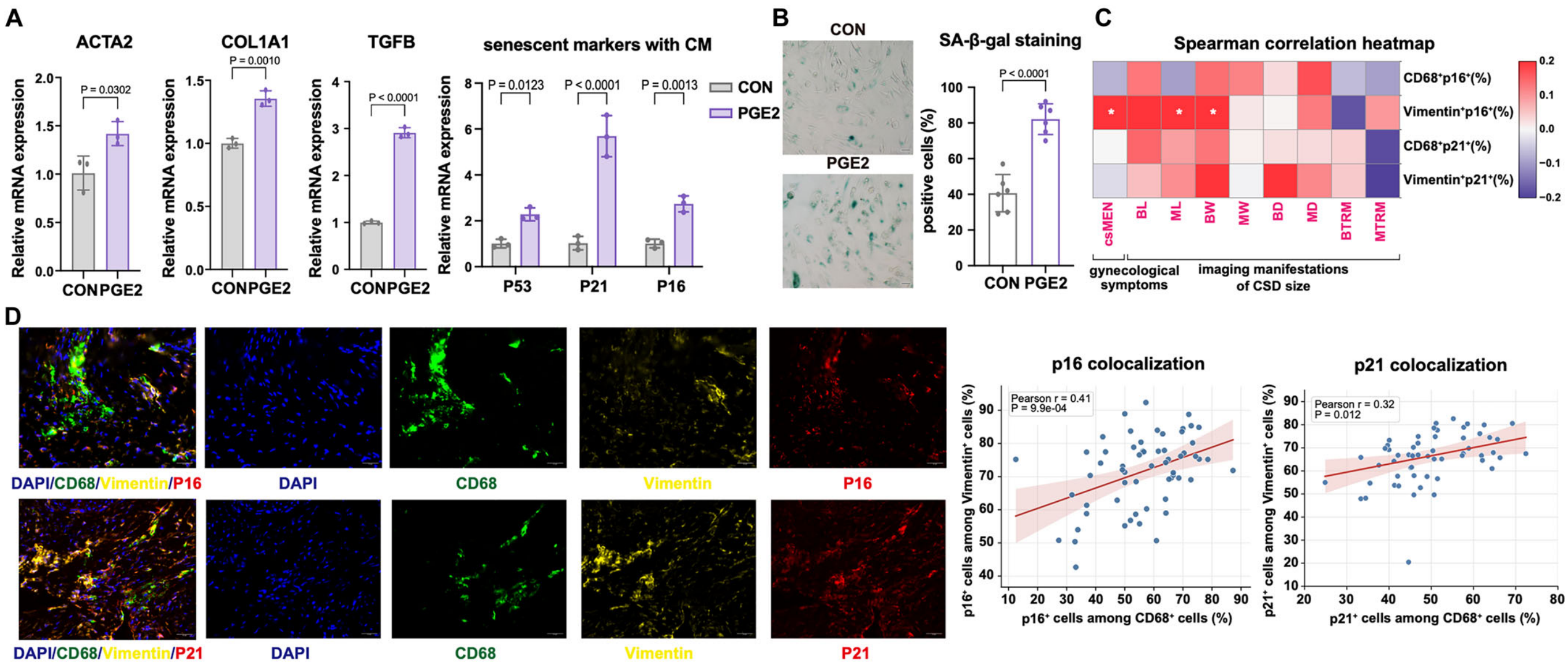

Figure EV5. Senescent macrophages drive fibroblast senescence and fibrosis.

(**A**) Relative mRNA expression levels of fibroblast activation and cellular senescence markers in utFBs following treatment with CM from PGE2-induced senescent macrophages. For CON versus PGE2, $P = 7.26 \times 10^{-6}$ for TGFB and $P = 6.20 \times 10^{-8}$ for P21. (**B**) Representative images of SA-β-gal staining (left) and frequency of positive staining cells among total cells (right) in utFBs after treatment with CM from PGE2-induced-senescent macrophages. Scale bars, 50 μm. For CON versus PGE2, $P = 2.06 \times 10^{-5}$. (**C**) Heatmap showing Spearman correlation coefficients between the colocalized expression of CD68/p16, CD68/p21, vimentin/p16, and vimentin/p21 detected by mIF and clinical characteristics of patients with CSD. Detailed clinical characteristics are provided in Fig. EV1H. Color intensity represents the magnitude of the correlation coefficient. $^{*}P < 0.05$. (**D**) Representative mIF images of CSD TMAs (left) and correlation analysis of p16/p21-positive senescent macrophages and p16/p21-positive senescent fibroblasts (right) ($n = 59$-60). Scale bars, 25 μm. Data are mean ± s.d. unpaired *t* test (**A**, **B**); two-way ANOVA with Šídák's multiple comparisons test (**A**); Spearman correlations analysis (**C**). Independent biological samples were used; exact numbers of biological replicates are listed in each panel. In vitro cell experiments were conducted with biological triplicates or more. Source data are available online for this figure.

 © The Author(s)

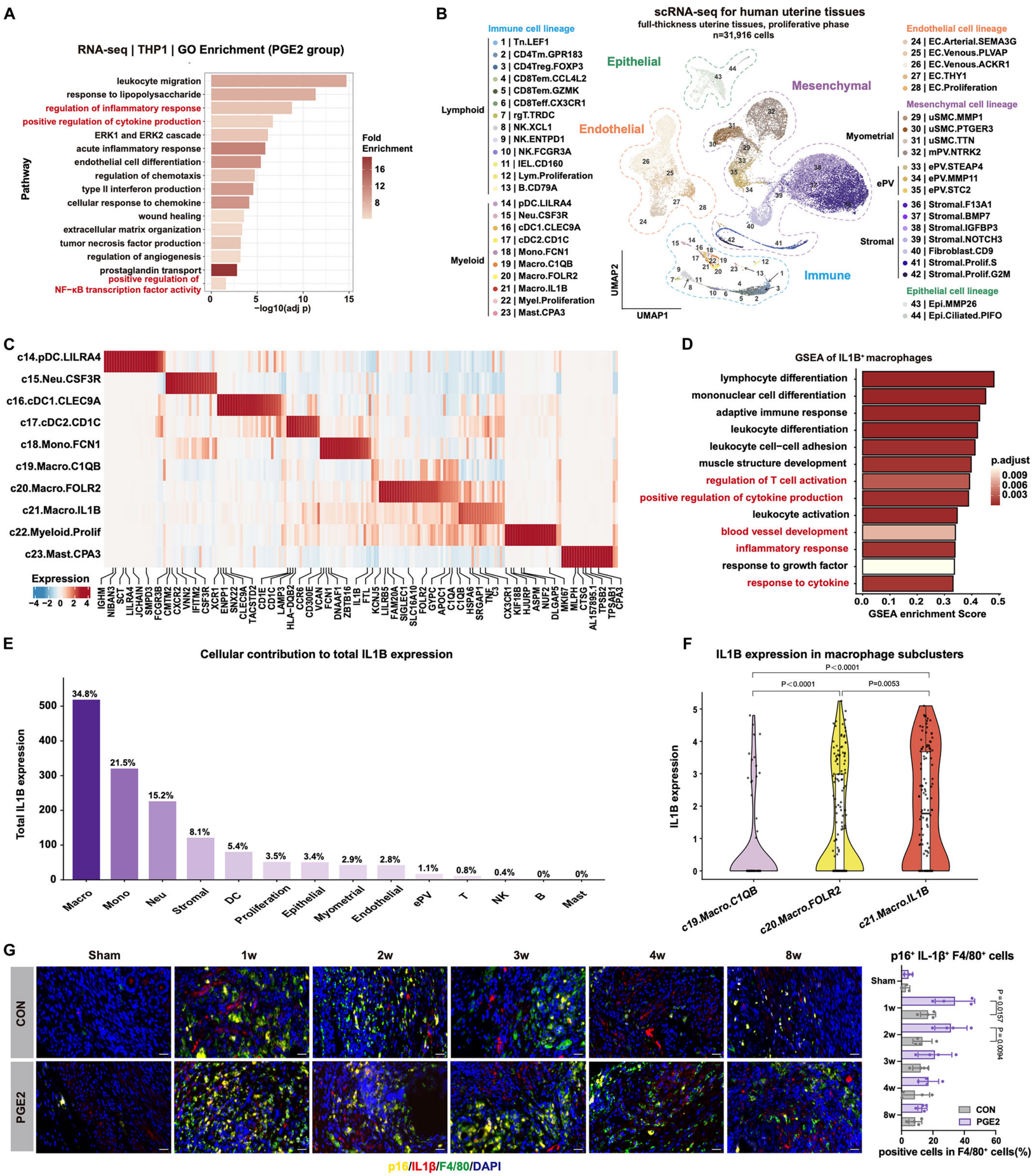
A
RNA-seq | THP1 | GO Enrichment (PGE2 group)
leukocyte migration
response to lipopolysaccharide
regulation of inflammatory response
positive regulation of cytokine production
ERK1 and ERK2 cascade
acute inflammatory response
endothelial cell differentiation
regulation of chemotaxis
type II interferon production
cellular response to chemokine
wound healing
extracellular matrix organization
tumor necrosis factor production
regulation of angiogenesis
prostaglandin transport
positive regulation of NF-κB transcription factor activity
Pathway
−log10(adj p)
Fold Enrichment
B
scRNA-seq for human uterine tissues
full-thickness uterine tissues, proliferative phase
n=31,916 cells
Immune cell lineage
Lymphoid
1 | Tn.LEF1
2 | CD4Tm.GPR183
3 | CD4Treg.FOXP3
4 | CD8Tem.CCL4L2
5 | CD8Tem.GZMK
6 | CD8Teff.CX3CR1
7 | rgT.TRDC
8 | NK.XCL1
9 | NK.ENTPD1
10 | NK.FCGR3A
11 | IEL.CD160
12 | Lym.Proliferation
13 | B.CD79A
Myeloid
14 | pDC.LILRA4
15 | Neu.CSF3R
16 | cDC1.CLEC9A
17 | cDC2.CD1C
18 | Mono.FCN1
19 | Macro.C1QB
20 | Macro.FOLR2
21 | Macro.IL1B
22 | Myel.Proliferation
23 | Mast.CPA3
Epithelial
Endothelial
Mesenchymal
Immune
UMAP1
UMAP2
Endothelial cell lineage
24 | EC.Arterial.SEMA3G
25 | EC.Venous.PLVAP
26 | EC.Venous.ACKR1
27 | EC.THY1
28 | EC.Proliferation
Mesenchymal cell lineage
Myometrial
29 | uSMC.MMP1
30 | uSMC.PTGER3
31 | uSMC.TTN
32 | mPV.NTRK2
ePV
33 | ePV.STEAP4
34 | ePV.MMP11
35 | ePV.STC2
Stromal
36 | Stromal.F13A1
37 | Stromal.BMP7
38 | Stromal.IGFBP3
39 | Stromal.NOTCH3
40 | Fibroblast.CD9
41 | Stromal.Prolif.S
42 | Stromal.Prolif.G2M
Epithelial cell lineage
43 | Epi.MMP26
44 | Epi.Ciliated.PIFO
C
c14.pDC.LILRA4
c15.Neu.CSF3R
c16.cDC1.CLEC9A
c17.cDC2.CD1C
c18.Mono.FCN1
c19.Macro.C1QB
c20.Macro.FOLR2
c21.Macro.IL1B
c22.Myeloid.Prolif
c23.Mast.CPA3
Expression
D
GSEA of IL1B⁺ macrophages
lymphocyte differentiation
mononuclear cell differentiation
adaptive immune response
leukocyte differentiation
leukocyte cell−cell adhesion
muscle structure development
regulation of T cell activation
positive regulation of cytokine production
leukocyte activation
blood vessel development
inflammatory response
response to growth factor
response to cytokine
p.adjust
GSEA enrichment Score
E
Cellular contribution to total IL1B expression
Total IL1B expression
34.8%
21.5%
15.2%
8.1%
5.4%
3.5%
3.4%
2.9%
2.8%
1.1%
0.8%
0.4%
0%
0%
Macro
Mono
Neu
Stromal
DC
Proliferation
Epithelial
Myometrial
Endothelial
ePV
T
NK
B
Mast
F
IL1B expression in macrophage subclusters
P<0.0001
P<0.0001
P=0.0053
IL1B expression
c19.Macro.C1QB
c20.Macro.FOLR2
c21.Macro.IL1B
G
Sham
1w
2w
3w
4w
8w
CON
PGE2
p16/IL1β/F4/80/DAPI
p16⁺ IL-1β⁺ F4/80⁺ cells
P=0.0157
P=0.0094
CON
PGE2
positive cells in F4/80⁺ cells(%)

Figure EV6. *IL1B*$^{+}$ macrophages represent a pathogenic macrophage subset associated with impaired uterine repair.

(**A**) GO enrichment pathway analysis on DEGs of THP1-derived macrophages treated with PGE2. (**B**) UMAP of scRNA-seq data from full-thickness CSD uterine tissues and healthy control uterine tissues ($n = 3$ per group). (**C**) Heatmap depicting scaled expression of marker genes for each myeloid subcluster in scRNA-seq. (**D**) Gene set enrichment analysis (GSEA) (GO biological processes) on DEGs of *IL1B*$^{+}$ macrophages. (**E**) Bar plot showing the relative contribution of each cell type to total *IL1B* expression in scRNA-seq dataset. For each cell type, the contribution was calculated as the product of cell number and mean *IL1B* expression, divided by the summed products across all cell types. Percentages above the bars indicate the relative contribution of each cell type to total *IL1B* expression. (**F**) *IL1B* expression levels across macrophage subsets. Dots represent individual cells. In the embedded box plots, the center line indicates the median, the box bounds indicate the 25th and 75th percentiles, and the whiskers indicate the minimum and maximum values. c19 vs c20, p.adj 2.10×10^{-5}; c19 vs c21p.adj 5.90×10^{-10}. (**G**) Representative mIF images (left) and proportion of IL1β^{+} senescent macrophages (right) in over-time mouse CS models ($n = 3$–4 per group). Scale bars, 25 µm. Data are mean ± s.d. GO enrichment analysis was performed using a hypergeometric test and Benjamini–Hochberg correction for multiple testing (**A**, **D**); two-sided Wilcoxon rank-sum test (**F**); two-way ANOVA with Šídák's multiple comparisons test (**G**). Independent biological samples were used; exact numbers of biological replicates are listed in each panel. Source data are available online for this figure.

 © The Author(s)

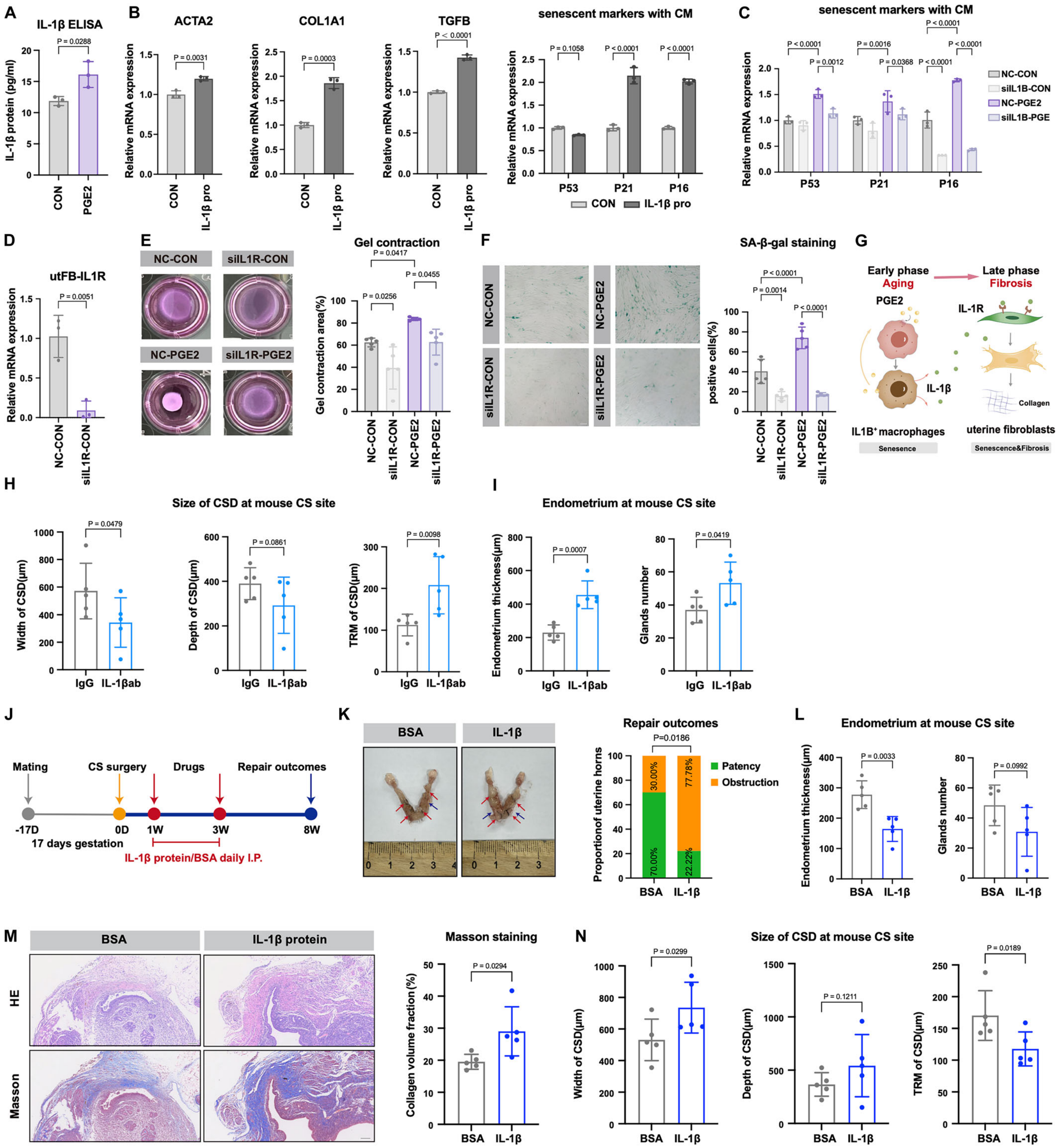
A
IL-1β ELISA
B
ACTA2
COL1A1
TGFB
senescent markers with CM
C
senescent markers with CM
D
utFB-IL1R
E
Gel contraction
F
SA-β-gal staining
G
Early phase Aging
Late phase Fibrosis
PGE2
IL-1R
IL-1β
Collagen
IL1B⁺ macrophages
uterine fibroblasts
H
Size of CSD at mouse CS site
I
Endometrium at mouse CS site
J
Mating
CS surgery
Drugs
Repair outcomes
17 days gestation
IL-1β protein/BSA daily I.P.
K
Repair outcomes
L
Endometrium at mouse CS site
M
Masson staining
N
Size of CSD at mouse CS site

Figure EV7. Blocking or supplementing IL-1β differentially regulates uterine incision fibrosis after CS.

(**A**) ELISA quantification of IL-1β levels in CM from PGE2-induced senescence macrophages. (**B**) Relative mRNA expression levels of fibroblast activation and cellular senescence markers in utFBs after treatment with IL-1β protein. For CON versus IL-1β protein, $P = 3.30 \times 10^{-5}$ for TGFB, $P = 2.41 \times 10^{-9}$ for P21, and $P = 9.06 \times 10^{-9}$ for P16. (**C**) Relative mRNA expression levels of cellular senescence markers in utFBs after treatment with CM from *IL1B* knockdown macrophages with/without PGE2 treatment. For P53, $P = 2.43 \times 10^{-5}$ for NC-CON versus NC-PGE2. For P16, $P = 2.18 \times 10^{-7}$, 2.53×10^{-8}, and 2.87×10^{-13} for NC-CON versus siIL1B-CON, NC-CON versus NC-PGE2, and NC-PGE2 versus siIL1B-PGE2, respectively. (**D**) Relative mRNA expression levels of *IL1R* in utFBs after *IL1R* knockdown. (**E**, **F**) Representative images (left) and quantification (right) of gel contraction and SA-β-gal staining in *IL1R* knockdown utFBs after treatment with CM from PGE2-induced senescence macrophages. Scale bars, 100 µm. $P = 5.99 \times 10^{-5}$ for NC-CON versus NC-PGE2 and $P = 7.05 \times 10^{-8}$ for NC-PGE2 versus siIL1R-PGE2. (**G**) Scheme of *IL1B*$^{+}$ macrophages transmitting senescence to induce uterine fibroblasts' senescence and fibrosis via IL-1β/IL1R axis. (**H**, **I**) Quantification of sizes of injury sites (**H**), endometrial thickness (**I**, left), and number of glands (**I**, right) at CS sites in mouse models at 8 weeks post-CS ($n = 5$ per group). (**J**) Time scheme illustrating the establishment and analysis of mouse CS models with IL-1β protein treatment. (**K**) Representative gross morphological images of uteri and patency analysis in mouse CS models at 8 weeks post-CS ($n = 5$ per group), with red arrows indicating CS suture endpoints and blue arrows marking obstructions. (**L**) Quantification of endometrial thickness (left), and number of glands (right) at CS sites in mouse models at 8 weeks post-CS ($n = 5$ per group). (**M**) Representative images of HE and Masson staining (left), and quantification of collagen volume fraction (right) at CS sites in mouse models at 8 weeks post-CS ($n = 5$ per group). Scale bars, 100 µm. (**N**) Quantification of sizes of injury sites at CS sites in mouse models at 8 weeks post-CS ($n = 5$ per group). Data are mean ± s.d. unpaired *t* test (**A**, **B**, **D**, **H**, **I**, **L–N**); two-way ANOVA with Tukey's or Šídák's multiple comparisons test (**B**, **C**); one-way ANOVA with Tukey's multiple comparisons test (**E**, **F**); Chi-squared test with one-tailed *P* value (**K**). Independent biological samples were used; exact numbers of biological replicates are listed in each panel. In vitro cell experiments were conducted with biological triplicates or more. Source data are available online for this figure.

 © The Author(s)

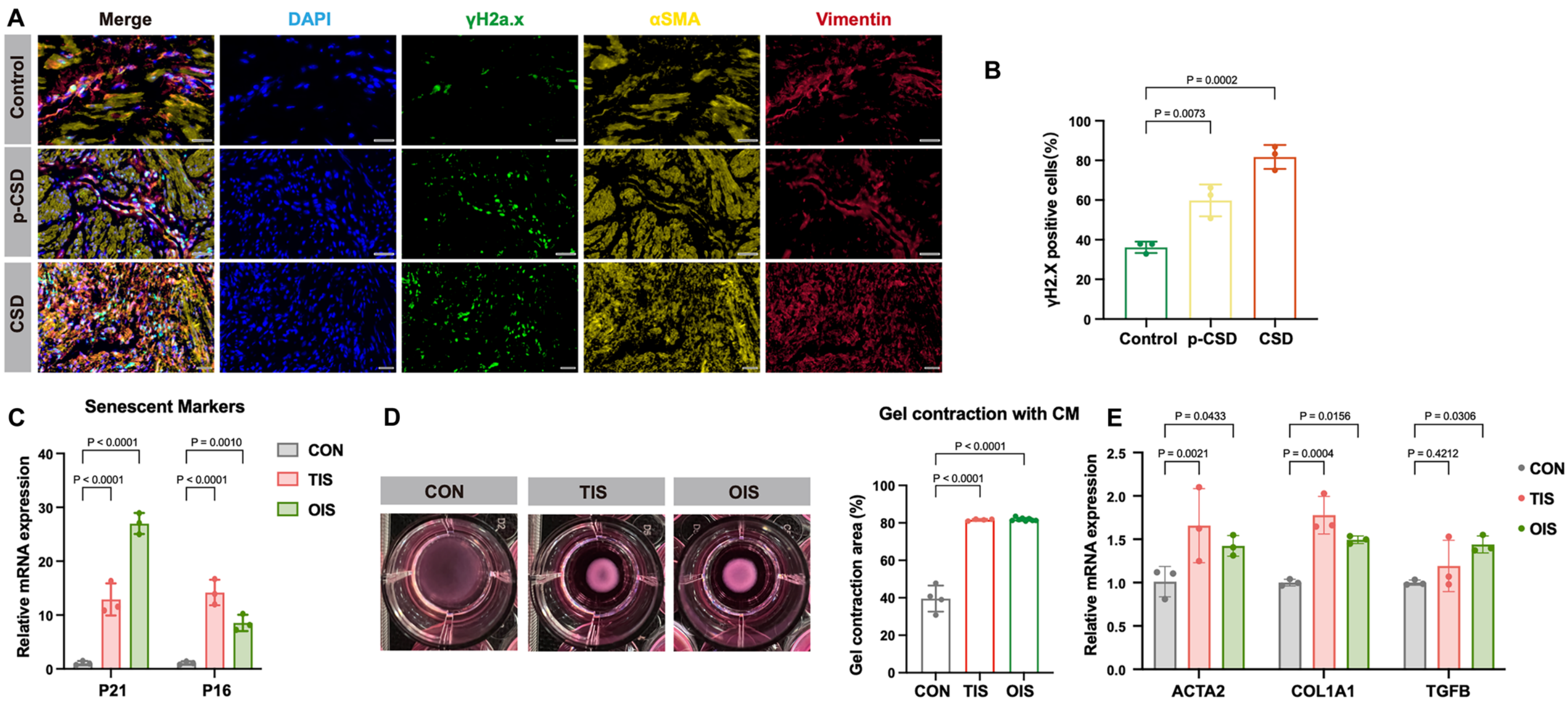

Figure EV8. Senescent fibroblasts drive late-stage fibrotic niche formation.

(**A**, **B**) Representative IF images (**A**) and frequency as a percentage of total cells (**B**) of γH2a.x expression in human uterine tissues ($n = 3$ per group). Scale bars, 25 μm. (**C**) Relative mRNA expression of cellular senescence markers in induced-senescence utFBs. OIS H_2O_2-induced senescence, TIS therapy-induced senescence. For P21, $P = 1.36 \times 10^{-5}$ and 2.47×10^{-9} for CON versus TIS and CON versus OIS, respectively; for P16, $P = 4.76 \times 10^{-6}$ for CON versus TIS. (**D**) Representative images (left) and quantification (right) of gel contraction in utFBs after treatment with CM from induced-senescence utFBs. $P = 6.19 \times 10^{-10}$ and 9.51×10^{-11} for CON versus TIS and CON versus OIS, respectively. (**E**) Relative mRNA expression levels of fibrosis markers in utFBs after treatment with CM from induced-senescence utFBs. Data are mean ± s.d. One-way ANOVA with Tukey's multiple comparisons test (**B**, **D**). Two-way ANOVA with Tukey's multiple comparisons test (**C**, **E**). Biological replicates represent individual patients, with exact numbers listed in each panel. In vitro cell experiments were conducted with biological triplicates or more. Source data are available online for this figure.

© The Author(s)

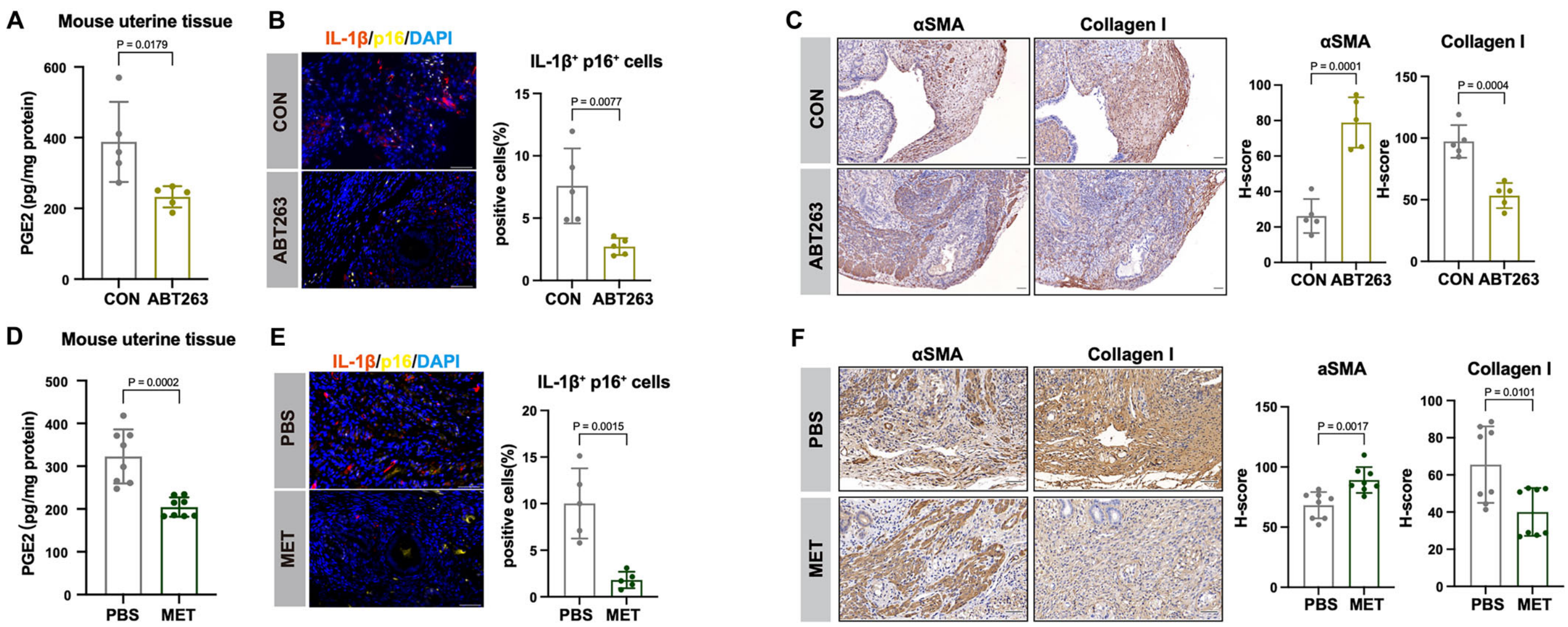

Figure EV9. Targeting aging pathways promotes uterine incision repair after CS.

(**A**, **D**) Quantification of PGE2 levels in mouse CS uterine tissues at 8 weeks post-CS by ELISA ($n = 5$–8 per group). (**B**, **E**) Representative images (left) and frequency of IL-1β$^+$ senescent cells in total cells (right) in mouse CS uterine tissues at 8 weeks post-CS ($n = 5$ per group). Scale bars, 25 µm. (**C**, **F**) Representative IHC images of αSMA and collagen I expression (left) and quantification of H-score for αSMA and collagen I (right) at CS sites in mouse models at 8 weeks post-CS ($n = 5$–8 per group). Scale bars, 50 µm. Data are mean ± s.d. Unpaired *t* test (**A**–**F**). Biological replicates represent individual patients, with exact numbers listed in each panel. Source data are available online for this figure.

 © The Author(s)
