# Supplementary material for: Prostaglandin E2-mediated aging microenvironment fuels pathogenic fibrosis in cesarean section scar defect
Source: EMBO Mol Med. 2026 Oct 2;18(11):4300–33. doi: 10.1038/s44321-026-00521-6 (PMC13748614; doi:10.1038/s44321-026-00521-6)
Supplement: Supplementary file 2 [file 44321_2026_521_MOESM1_ESM.pdf]

EMBO Molecular Medicine

PGE2-mediated aging microenvironment fuels pathogenic fibrosis in cesarean section scar defect

Yujia Yin, Huihui Chen, Weiwei Xie, Yizhi Wang, Yujing Qian, Rong Sheng, Danning Li, Jiarui Li, Xingchen Zhou, Zhiyuan Dai, Zhiyong Mao, Ziyi Li, and Xipeng Wang

Corresponding authors: Xipeng Wang (wangxipeng@xinhuamed.com.cn), Ziyi Li (lzy@ism.pumc.edu.cn)

Review Timeline:

Submission Date:	24th Mar 26
Editorial Decision:	29th Apr 26
Revision Received:	5th Aug 26
Editorial Decision:	19th Aug 26
Revision Received:	29th Aug 26
Accepted:	2nd Sep 26

Editor: Jingyi Hou

Transaction Report:

(Note: With the exception of the correction of typographical or spelling errors that could be a source of ambiguity, letters and reports are not edited. Depending on transfer agreements, referee reports obtained elsewhere may or may not be included in this compilation. Referee reports are anonymous unless the Referee chooses to sign their reports.)

29th Apr 2026

Dear Prof. Wang,

Thank you for submitting your work to EMBO Molecular Medicine. We have now heard back from the two reviewers who agreed to evaluate your manuscript. Overall, the reviewers find your study of interest but have raised several important concerns that will need to be addressed in a major revision of the manuscript.

As detailed below, Reviewer #2 is enthusiastic and raises only relatively minor issues, whereas Reviewer #1 identifies a number of concerns, particularly regarding the initial metabolomics analysis, and requests additional experiments to strengthen the mechanistic interpretation and causal conclusions.

In addressing the reviewers' comments, we recommend the following:

• With respect to Reviewer #1's concerns about the metabolomics analysis, increasing the sample size to some extent would be advisable.
• Regarding Reviewer #1's comments 2, 5, and 7, we would not require additional experiments; however, these points should be discussed as limitations or future directions.
• All other issues should be addressed.

As you may know, our editorial policy generally allows for a single round of major revision, so it is important to provide responses to the reviewers' comments that are as complete as possible. Please feel free to contact me if you would like to discuss any of the issues raised-either via email or a video call.

EMBO Molecular Medicine has a "scooping protection" policy, whereby similar findings that are published by others during review or revision are not a criterion for rejection. Should you decide to submit a revised version, I do ask that you get in touch after three months if you have not completed it, to update us on the status.

Please also contact us as soon as possible if similar work is published elsewhere. If other work is published, we may not be able to extend the revision period beyond three months.

Please read below for important editorial formatting and consult our author's guidelines for proper formatting of your revised article for EMBO Molecular Medicine.

I look forward to receiving your revised manuscript.

Sincerely,
Jingyi

Jingyi Hou
Senior Editor
EMBO Molecular Medicine

*****
When preparing your revised manuscript, please refer to our guidelines: https://link.springer.com/journal/44321/submission-guidelines#cms-Revised-submissions. We perform an initial quality control of all revised manuscripts before re-review; failure to include requested items will delay the evaluation of your revision.

We require:

1) A .docx formatted version of the manuscript text (including legends for main figures, EV figures and tables). Please make sure that the changes are highlighted to be clearly visible.

2) Individual production quality figure files as .eps, .tif, .jpg (one file per figure). For guidance, download the 'Figure Guide PDF': https://media.springernature.com/original/springer-cms/rest/v1/content/27825798/data/v1.

3) A .docx formatted letter INCLUDING the reviewers' reports and your detailed point-by-point responses to their comments. As

part of the EMBO Press transparent editorial process, the point-by-point response is part of the Review Process File (RPF), which will be published alongside your paper.

4) A complete author checklist, which you can download from our author guidelines. Please insert information in the checklist that is also reflected in the manuscript. The completed author checklist will also be part of the RPF.

5) Please note that all corresponding authors are required to supply an ORCID ID for their name upon submission of a revised manuscript.

6) It is mandatory to include a 'Data Availability' section after the Materials and Methods. Before submitting your revision, primary datasets produced in this study need to be deposited in an appropriate public database, and the accession numbers and database listed under 'Data Availability'. Please remember to provide a reviewer password if the datasets are not yet public.

In case you have no data that requires deposition in a public database, please state so in this section. Note that the Data Availability Section is restricted to new primary data that are part of this study.

7) For data quantification: please specify the name of the statistical test used to generate error bars and P values, the number (n) of independent experiments (specify technical or biological replicates) underlying each data point and the test used to calculate p-values in each figure legend. The figure legends should contain a basic description of n, P and the test applied. Graphs must include a description of the bars and the error bars (s.d., s.e.m.).

8) At EMBO Press we ask authors to provide source data for the main manuscript figures. You will receive a separate email with instructions for providing source data with your revised manuscript, including how to upload and organize the files.

9) Our journal encourages inclusion of *data citations in the reference list* to directly cite datasets that were re-used and obtained from public databases. Data citations in the article text are distinct from normal bibliographical citations and should directly link to the database records from which the data can be accessed. In the main text, data citations are formatted as follows: "Data ref: Smith et al, 2001" or "Data ref: NCBI Sequence Read Archive PRJNA342805, 2017". In the Reference list, data citations must be labeled with "[DATASET]". A data reference must provide the database name, accession number/identifiers and a resolvable link to the landing page from which the data can be accessed at the end of the reference.

10) We replaced Supplementary Information with Expanded View (EV) Figures and Tables that are collapsible/expandable online. A maximum of 5 EV Figures can be typeset. EV Figures should be cited as 'Figure EV1, Figure EV2" etc... in the text and their respective legends should be included in the main text after the legends of regular figures.

- For the figures that you do NOT wish to display as Expanded View figures, they should be bundled together with their legends in a single PDF file called *Appendix*, which should start with a short Table of Content. Appendix figures should be referred to in the main text as: "Appendix Figure S1, Appendix Figure S2" etc.

- Additional Tables/Datasets should be labeled and referred to as Table EV1, Dataset EV1, etc. Legends have to be provided in a separate tab in case of .xls files. Alternatively, the legend can be supplied as a separate text file (README) and zipped together with the Table/Dataset file.

11) The paper explained: EMBO Molecular Medicine articles are accompanied by a summary of the articles to emphasize the major findings in the paper and their medical implications for the non-specialist reader. Please provide a draft summary of your article highlighting

- the medical issue you are addressing,

- the results obtained and

- their clinical impact.

This may be edited to ensure that readers understand the significance and context of the research. Please refer to any of our published articles for an example.

12) Author contributions: You will be asked to provide CRediT (Contributor Role Taxonomy) terms in the submission system. These replace a narrative author contribution section in the manuscript.

13) A Conflict of Interest statement should be provided in the main text.

14) Please provide a 'Synopsis' to further enhance discoverability. Synopses are displayed on the journal webpage and are freely accessible to all readers. They include a short stand first (maximum of 300 characters, including space) as well as 2-5

one-sentences bullet points that summarizes the paper. Please write the bullet points to summarize the key NEW findings. They should be designed to be complementary to the abstract - i.e. not repeat the same text. We encourage inclusion of key acronyms and quantitative information (maximum of 30 words / bullet point). Please use the passive voice. Please attach these in a separate file or send them by email, we will incorporate them accordingly.

Please provide a visual abstract to illustrate your article as a PNG file 550 px wide x 300 px high.

15) All Materials and Methods need to be described in the main text using our 'Structured Methods' format. According to this format, the Methods section includes a Reagents and Tools Table (listing key reagents, experimental models, software and relevant equipment and including their sources and relevant identifiers) followed by a Methods and Protocols section describing the methods, ideally using a step-by-step protocol format. The aim is to facilitate adoption of the methodologies across labs.

Please download and fill our Reagents and Tools Table template (.docx), which you can find in our author guidelines: https://link.springer.com/journal/44320/submission-guidelines#structuredmethods.

When submitting your revised manuscript, please do not include the Reagents and Tools Table in the Methods section of the manuscript but upload it as a separate file choosing the file type "Reagent Table".

EMBO Molecular Medicine has a "scooping protection" policy, whereby similar findings that are published by others during review or revision are not a criterion for rejection. Should you decide to submit a revised version, I do ask that you get in touch after three months if you have not completed it, to update us on the status.

***** Reviewer's comments *****

Referee #1 (Remarks for Author):

This study by Yin and colleagues investigates the molecular mechanisms underlying cesarean section scar defect (CSD), a common and clinically challenging complication following cesarean section. The authors demonstrate that elevated PGE2 preferentially induces early senescence in macrophages, which in turn transmit pro-senescent signals to uterine fibroblasts via IL-1β/IL-1R signaling, establishing a macrophage-fibroblast senescence relay that promotes excessive fibroblast activation and progressive fibrosis. Additionally, inhibition of the PGES/PGE2 pathway, as well as anti-aging interventions including the senolytic ABT263 and the senomorphic agent metformin, significantly attenuate fibrosis and improve uterine structural and functional recovery in preclinical models. A prospective clinical trial further suggests that adjunctive metformin treatment may enhance surgical outcomes in CSD patients. Despite the study's strengths, several methodological and conceptual limitations warrant careful consideration.

1. It is well known that metabolomics analysis requires a sufficient sample size due to significant individual variability; generally, each group should contain more than 30 samples. The sample size in this study is too small, with only 3 samples per group, which compromises the reproducibility of the results.
2. The authors' claim that PGE2 first promotes macrophage senescence, and that senescent macrophages subsequently induce fibroblast senescence and fibrosis, lacks sufficient evidence. The temporal and causal relationship between macrophage senescence and fibroblast senescence is inferred from correlative in vivo time-course data and in vitro co-culture experiments, but definitive lineage-tracing or selective depletion studies (e.g., conditional depletion of senescent macrophages in vivo) are lacking to establish that macrophage senescence is both necessary and sufficient for subsequent fibroblast senescence and fibrosis.
3. The claim that PGE2 preferentially induces macrophage senescence over other cell types is based largely on in vitro assays with a limited set of cell lines; primary human uterine macrophages and fibroblasts were not used for validation, leaving open the possibility that cell-type-specific responses in vivo may differ due to microenvironmental cues.
4. For Figure 4, the senescence of fibroblasts also increases significantly during the first week and remains at a high level. The number of fibroblasts far exceeds that of macrophages; therefore, even though the proportion of senescent fibroblasts is lower than that of macrophages, the harm they cause may be far greater.
5. This study mainly clarifies the observed phenomena but lacks mechanistic exploration. For example, why does elevated PGE2 promote IL1β expression? What is the molecular mechanism? Why do ABT263 and metformin inhibit PGE2 expression? Elucidating these mechanisms would be more meaningful.
6. What is the expression pattern of IL1β in human CSD tissue specimens? Co-staining with macrophages, fibroblasts, endothelial cells, and other immune cells should be performed to determine whether macrophages are the primary source of IL1β. Furthermore, additional senescence markers in macrophages-such as P21, P16, and P53-need to be examined in human specimens, along with the correlation between fibroblast senescence and fibrosis.
7. The proposed IL-1β/IL-1R axis as the key paracrine mediator is supported by siRNA knockdown experiments, but the absence of in vivo cell-type-specific genetic ablation of Il1b in macrophages or Il1r1 in fibroblasts limits the strength of causal inference.
8. The study would benefit from a more rigorous assessment of whether other SASP factors contribute redundantly, as IL-1β blockade only partially reversed fibroblast activation in some assays.

9. Extended Data Fig. 7: The two senescence models shown differ from the authors' hypothesis that IL1β induces fibroblast senescence.
10. For clinical trial, the observed improvements in menstrual duration and residual myometrial thickness, while promising, should be interpreted as hypothesis-generating rather than definitive evidence of efficacy. From a mechanistic standpoint, the bulk RNA-seq analysis of patient tissues after metformin treatment shows pathway-level changes but does not directly demonstrate reduced senescent cell burden or PGE2 signaling in the same patients, leaving a gap between the proposed mechanism and clinical response. Finally, the manuscript lacks a clear discussion of potential off-target effects of the anti-aging interventions (e.g., ABT263, metformin) on normal tissue repair or reproductive outcomes beyond fibrosis, which is critical given the uterus's regenerative demands and the known dual roles of PGE2 in muscle homeostasis and inflammation resolution.

Referee #2 (Comments on Novelty/Model System for Author):

The authors have employed appropriate experimental models and provided a comprehensive analysis of their hypothesis, which is sufficient to support publication of the manuscript. In future studies, the use of genetic approaches to selectively deplete senescent macrophages (inducible depletion model) would be valuable to further delineate their specific contribution to CSD development and progression.

Referee #2 (Remarks for Author):

The manuscript entitled "Prostaglandin E2-mediated aging microenvironment fuels pathogenic fibrosis in cesarean section scar defect" investigates the biological mechanisms underlying cesarean section scar defect (CSD), identifying the PGES/PGE2 signaling pathway as a key driver of fibrosis and impaired uterine healing. The study reveals that elevated PGE2 induces early macrophage senescence, which transmits pro-senescent signals to fibroblasts via IL-1β/IL-1R, leading to excessive fibrosis. Therapeutic interventions targeting PGE2 signaling and cellular senescence; such as inhibitors, senolytics. They further provide evidence that the anti-diabetic drug metformin, effectively improve uterine repair in mouse models and show promising results in a clinical trial. These findings suggest that modulating the senescence and inflammation pathways could enhance healing, prevent CSD and improve fertility outcomes after cesarean delivery.
The manuscript presents a comprehensive and well-structured investigation into the pathogenesis of CSD. The authors conducted a well-designed and comprehensive study, with an impressive integration of metabolomics, transcriptomics, in vitro and in vivo models, as well as clinical trial data. The work provides compelling support for the central hypothesis, identifies key cellular targets, and delineates the molecular pathways involved in disease pathogenesis. The inclusion of clinical samples, a prospective clinical trial with metformin, and validated mouse models strengthen the clinical applicability. The reported findings are novel and carry significant implications for the development of future therapeutic strategies aimed at the prevention of CSD. This manuscript provides a significant contribution to understanding the cellular and molecular mechanisms of CSD and introduces promising therapeutic strategies. The translational potential of targeting this pathway is well-supported, and the combination of experimental and clinical data is a major strength. With minor revisions for clarity, detail, and contextual discussion, it will be a valuable addition to the field. I strongly recommend acceptance of this manuscript to the Journal of EMBO Molecular Medicine after minor revisions.

Some minor comments/suggestions:

The Materials and Methods section is comprehensive, however,
1. Eensure that all experimental procedures, especially animal treatments and clinical trial design, include sufficient detail for reproducibility.
2. Clarify the timing of interventions relative to surgery/injury and repair phases, especially for therapeutic treatments.
3. The interim clinical results are promising, however, acknowledge limitations such as sample size, follow-up duration, and potential confounders.
4. Discuss plans for longer-term follow-up and larger randomized studies.
5. The description of the sham-operated control group is missing from the Methods section.
6. As standard practice, relative gene expression in qPCR experiments is typically normalized using the geometric mean of multiple housekeeping genes (generally three to five) to ensure robust and reliable normalization. In the present study, only ACTB was used as the reference gene (Page 27, line 609). Could the authors provide a justification for this choice? Additionally, please indicate whether the stability of ACTB expression was assessed and confirmed across the patient samples included in the analysis.

Result and Discussion:

7. Figure 1B: Progesterone levels appear to differ markedly between control, p-CSD, and CSD tissues; yet this is not discussed in the manuscript. The authors should comment on this pronounced difference and discuss its potential biological or clinical relevance. Progesterone is known to modulate inflammatory responses, prostaglandin signaling, angiogenesis, and extracellular matrix remodeling, all of which are key processes in wound repair. Although progesterone is unlikely to be a primary determinant of CSD formation (as it occurs postpartum), altered local progesterone signaling could influence tissue remodeling and scar

maturation. Clarification of whether this difference is mechanistically relevant or a consequence of CSD can help better interpretation of the data.
8. Based on Figure 1g, the authors may briefly comment on whether serum PGE_2 levels have potential utility as biomarkers for early detection or prognosis of CSD.
9. Differences between p-CSD and CSD: The manuscript does not sufficiently discuss the differences between the p-CSD and CSD groups. The heat map shown in Figure 1 display distinct expression patterns in these two conditions. The authors should comment on these differences to clarify.
10. Please discuss if patient-specific factors (e.g., age, hormonal status, surgical techniques) influence PGE2 levels, senescence, and fibrosis in CSD?
11. The rationale for administering metformin one month prior to VR surgery (Fig. 7A, page 14, line 303) is unclear. This design appears inconsistent with the murine experiments, in which metformin demonstrated a significant therapeutic effect even when administered post surgery (Fig. 6F-J). The authors should clarify the reasoning behind the preoperative treatment strategy for women and explain why a post-surgical intervention, which more closely aligned with the assessed animal model, was not evaluated.
12. A proportion of pregnant women with pre existing type 2 diabetes are treated with metformin prior to conception, and metformin is also commonly prescribed for the management of gestational diabetes mellitus (GDM). It is well established that cesarean section delivery and CSD is more frequent among women with diabetes, largely attributed to impaired wound healing. Both GDM and pre existing diabetes have been identified as significant patient related risk factors for postoperative wound complications. In this context, it remains unclear why metformin does not appear to confer a protective benefit in this population. Could the authors comment on this apparent lack of benefit? Additionally, have patient level data been analyzed to specifically assess this association?
13. Metformin is a pleiotropic drug with multiple cellular and metabolic targets. In the context of CSD, the specific mechanism/s by which metformin mediates the observed beneficial effects remain unclear. The authors should comment on the possible mechanism of action of metformin in the context of CSD.
14. While agents such as celecoxib and CAY10526 demonstrate efficacy in the mouse model, both are broad inhibitors of prostaglandin synthesis. As such, their effects may not be specific to pathways directly implicated in CSD pathogenesis. Potential off-target actions and systemic consequences, particularly in a clinical setting, warrant further discussion. The authors should address the limitations of broadly suppressing prostaglandin signaling and discuss the potential safety concerns and translational challenges associated with these agents in humans.
15. The clinical trial is an interim analysis with a small sample size (30 patients). Long-term outcomes, fertility data, and adverse event monitoring are necessary before definitive conclusions. Details on randomization, blinding, and controls should be mentioned to assess bias.

-

Review Response Letter

Response to Reviewer 1's Comments

Referee #1 (Remarks for Author):

This study by Yin and colleagues investigates the molecular mechanisms underlying cesarean section scar defect (CSD), a common and clinically challenging complication following cesarean section. The authors demonstrate that elevated PGE2 preferentially induces early senescence in macrophages, which in turn transmit pro-senescent signals to uterine fibroblasts via IL-1β/IL-1R signaling, establishing a macrophage-fibroblast senescence relay that promotes excessive fibroblast activation and progressive fibrosis. Additionally, inhibition of the PGES/PGE2 pathway, as well as anti-aging interventions including the senolytic ABT263 and the senomorphic agent metformin, significantly attenuate fibrosis and improve uterine structural and functional recovery in preclinical models. A prospective clinical trial further suggests that adjunctive metformin treatment may enhance surgical outcomes in CSD patients. Despite the study's strengths, several methodological and conceptual limitations warrant careful consideration.

1. It is well known that metabolomics analysis requires a sufficient sample size due to significant individual variability; generally, each group should contain more than 30 samples. The sample size in this study is too small, with only 3 samples per group, which compromises the reproducibility of the results.

Response: Thank you for this valuable suggestion. We agree that the original quasi-targeted discovery cohort was too small to provide stand-alone validation. We therefore retained it as a discovery dataset and added targeted LC-MS/MS validation in an expanded cohort comprising 15 paired CSD tissues, 15 paired para-CSD tissues, and 10 healthy control myometrial tissues (**Results Page5 Line83-89**). The targeted panel contained 42 metabolites, including arachidonic acid pathway intermediates and two candidates identified higher in healthy controls in the discovery analysis (**supplementary table 1**). This analysis confirmed increased PGE2 and other arachidonic acid metabolites in CSD-associated tissues and progesterone levels showed a consistent downward trend in CSD tissues (**Fig. 1d**) and para-CSD tissues (**Fig. EV1e**).

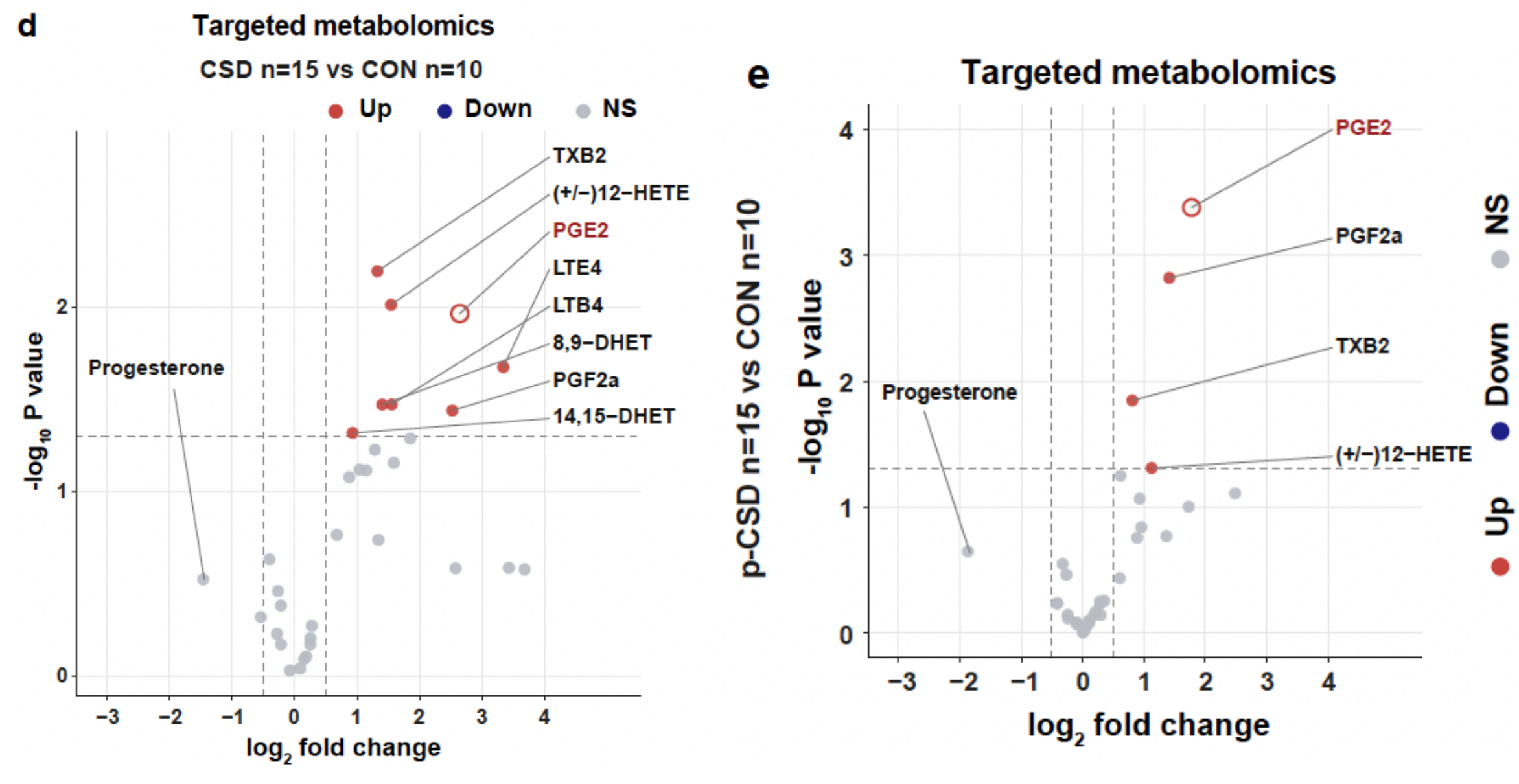

Fig. 1d, Volcano plot showing differential metabolites detected by targeted arachidonic acid metabolomics in CSD tissues (n = 15) and healthy control uterine tissues (n = 10).

Fig. EV1e, Volcano plot showing differential metabolites detected by targeted arachidonic acid metabolomics in para-CSD tissues (n = 15) and healthy control uterine tissues (n = 10).

2. The authors' claim that PGE2 first promotes macrophage senescence, and that senescent macrophages subsequently induce fibroblast senescence and fibrosis, lacks sufficient evidence. The temporal and causal relationship between

macrophage senescence and fibroblast senescence is inferred from correlative in vivo time-course data and in vitro co-culture experiments, but definitive lineage-tracing or selective depletion studies (e.g., conditional depletion of senescent macrophages in vivo) are lacking to establish that macrophage senescence is both necessary and sufficient for subsequent fibroblast senescence and fibrosis.

Response: We agree that the time-course, conditioned-medium, and co-culture experiments do not definitely support that macrophage senescence is necessary and sufficient for later fibroblast senescence in vivo. We also acknowledge that lineage tracing or selective depletion of senescent macrophages will be required to further establish a definitive causal hierarchy. We therefore moderated causal wording throughout the **Results** (**Page11 Line227-228**) and **Discussion** (**Page19 Line414-415**). The revised manuscript now describes that the data support a model in which macrophage senescence appears early and may contribute to later fibroblast senescence.

3. The claim that PGE2 preferentially induces macrophage senescence over other cell types is based largely on in vitro assays with a limited set of cell lines; primary human uterine macrophages and fibroblasts were not used for validation, leaving open the possibility that cell-type-specific responses in vivo may differ due to microenvironmental cues.

Response: Thank you for this comment. First, we would like to clarify that the uterine fibroblasts and smooth-muscle cells used here were primary human cells. We also added experiments using CD14-enriched primary human uterine macrophages, which showed increased p16 and reduced Ki-67 after PGE2 exposure (**Fig. EV3h-j**). THP-1-derived macrophages were retained for detailed mechanistic studies because primary uterine macrophages are scarce and would undergo fibroblasts contamination during ex vivo culture (**Page10 Line209-212**).

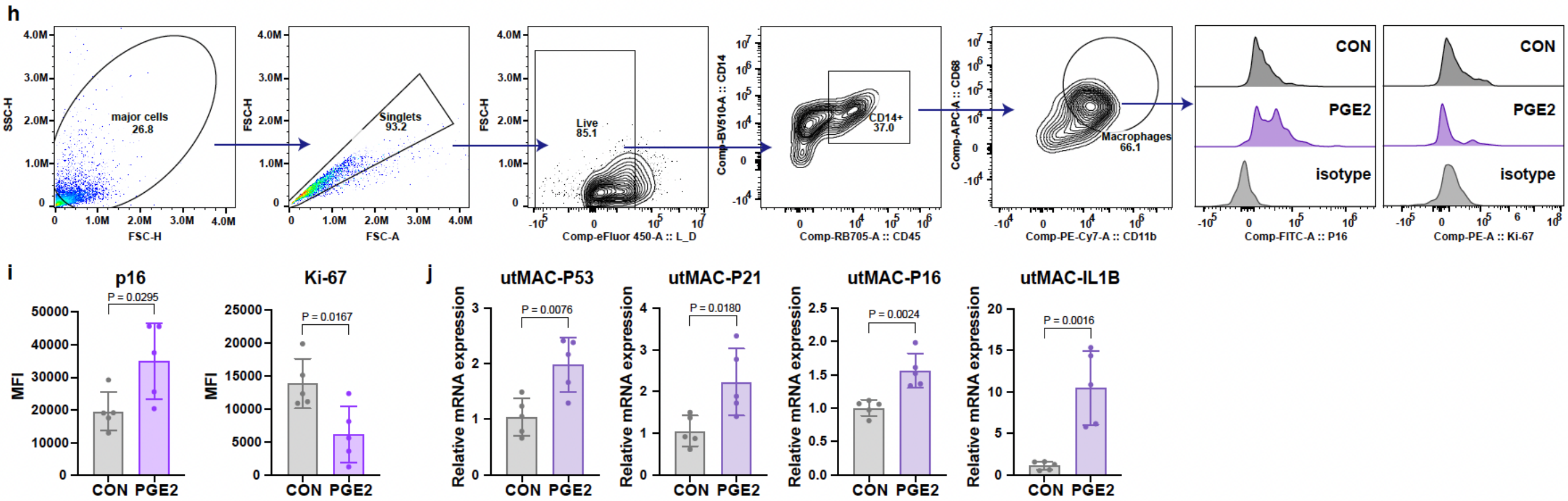

Fig. EV3h-i, Representative flow cytometry plots showing the isolation of human uterine macrophages, followed by PGE2 treatment, and quantification of p16 and Ki-67 expression by mean fluorescence intensity (MFI) (n=5). **j,** RT–qPCR of the relative mRNA expression of cellular senescent and SASP markers in human uterine macrophages following PGE2 treatment (n=5)**.**

4. For Fig. 4, the senescence of fibroblasts also increases significantly during the first week and remains at a high level. The number of fibroblasts far exceeds that of macrophages; therefore, even though the proportion of senescent fibroblasts is lower than that of macrophages, the harm they cause may be far greater.

Response: In our data, macrophages constitute the major senescent cell population at the early stage, with a higher proportion of senescence than fibroblasts. This temporal pattern is also consistent with the general sequence of wound healing, in which the inflammatory phase precedes fibroblast-driven tissue remodeling. This distinction does not imply that fibroblasts are less important. In our study, senescent fibroblasts may serve as major downstream effectors that sustain extracellular matrix deposition and amplify aging microenvironment. Future studies will be required to definitively validate this model and focus on the contribution of fibroblasts to CSD. We have clarified this point in the revised manuscript (**Discussion Page19 Line410-415**).

5. This study mainly clarifies the observed phenomena but lacks mechanistic exploration. For example, why does elevated PGE2 promote IL1β expression? What is the molecular mechanism? Why do ABT263 and metformin inhibit PGE2 expression? Elucidating these mechanisms would be more meaningful.

Response: In our current bulk RNA-seq analysis, we identified several candidate pathways that may be involved in PGE2-induced macrophage senescence, including NF-κB pathway, which may provide a plausible mechanistic link between elevated PGE2 and IL1β induction. However, detailed study of this mechanism is beyond the scope of our present study and will require more investigation in future work. In line with the editorial guidance, we therefore expanded the **Discussion** to outline these plausible mechanisms and future directions more clearly (**Page18 Line404-406**). We also clarified that lower PGE2 after ABT263 or metformin could reflect a reduced senescent-cell burden, broader inflammatory suppression, or other metabolic effects (**Fig. 6-7**, **Page17 Line369-371**).

6. What is the expression pattern of IL1β in human CSD tissue specimens? Co-staining with macrophages, fibroblasts, endothelial cells, and other immune cells should be performed to determine whether macrophages are the primary source of IL1β. Furthermore, additional senescence markers in macrophages-such as P21, P16, and P53-need to be examined in human specimens, along with the correlation between fibroblast senescence and fibrosis.

Response: We reanalyzed the human CSD single-cell dataset and found that macrophages represented the primary cellular source of *IL1B* expression compared with stromal, endothelial, and other immune-cell subsets (**Fig. EV6e-f**). Multiplex immunofluorescence further showed increased IL-1β$^+$ γH2AX$^+$ macrophages in CSD tissues in previous manuscript (**Fig. 5i**). To further validate additional senescence markers in macrophages and fibroblasts in human specimens, we added p16 and p21 co-staining with CD68 and vimentin in an independent TMA cohort, and observed positive correlations between macrophage and fibroblast senescence-associated signatures, CSD size, and residual myometrial thickness (**Fig. EV5c-d**). These findings support the importance of senescent macrophages as a key inflammatory component associated with impaired uterine repair and fibrotic progression.

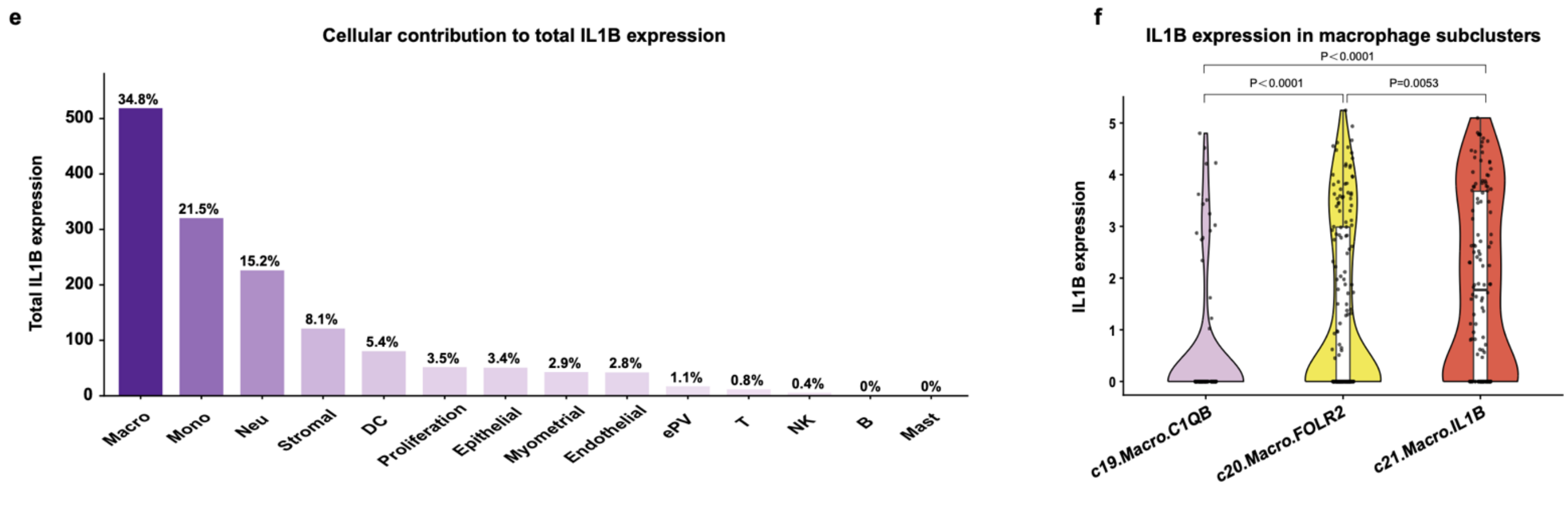

Fig. EV6e, Bar plot showing the relative contribution of each cell type to total *IL1B* expression in scRNA-seq dataset. For each cell type, the contribution was calculated as the product of cell number and mean *IL1B* expression, divided by the summed products across all cell types. Percentages above the bars indicate the relative contribution of each cell type to total *IL1B* expression. **f,** *IL1B* expression levels across macrophages subsets.

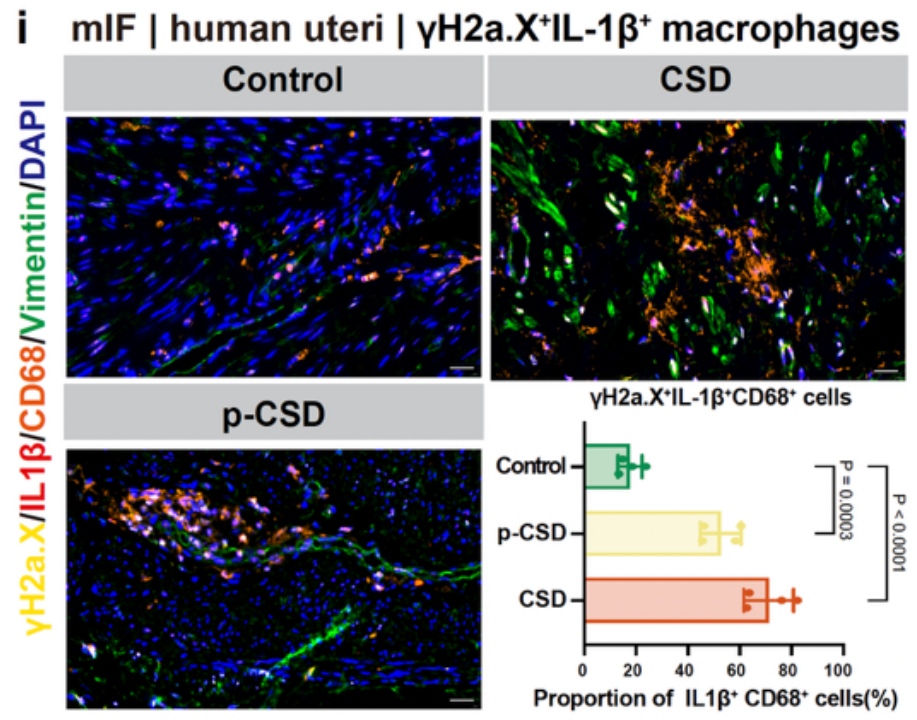

Fig. 5i, Representative mIF images (**left**) and proportion of IL1β^{+} senescent macrophages (**right**) in CSD and healthy patients (n = 4 per group). Scale bars, 25μm.

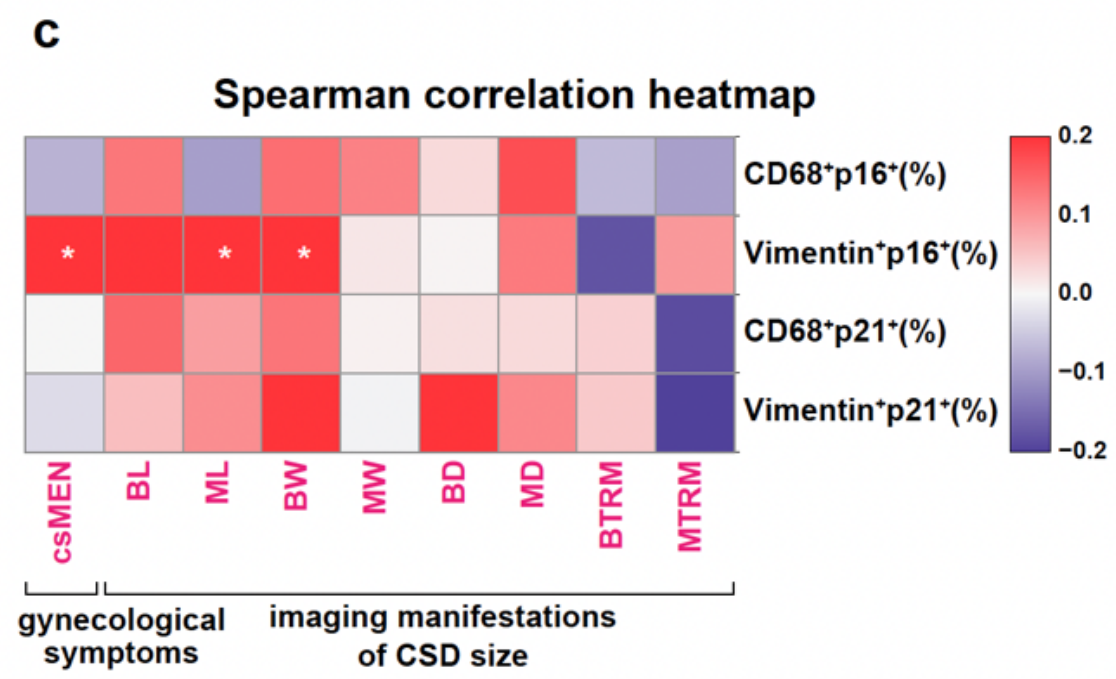

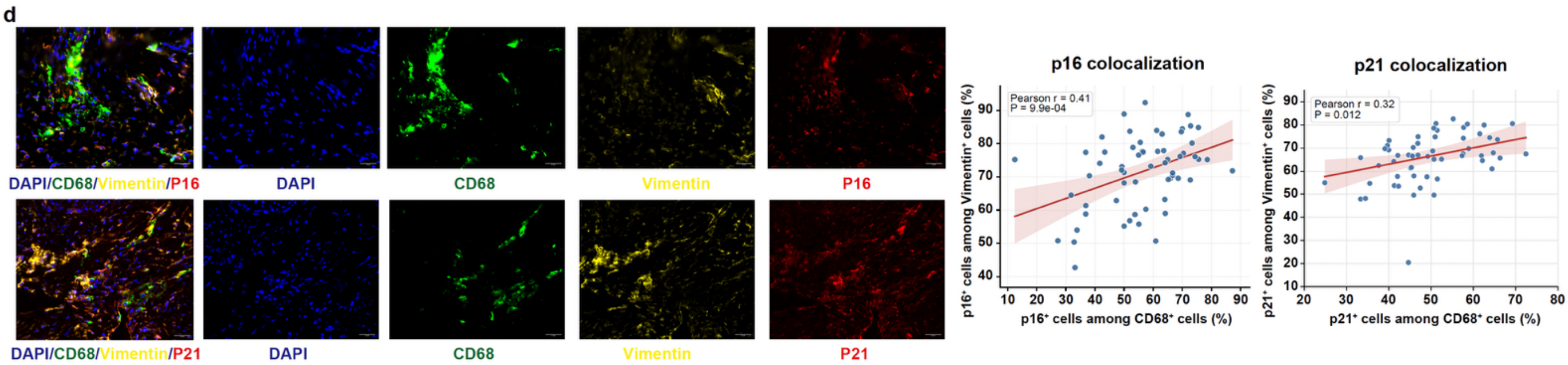

Fig. EV5c, Heatmap showing spearman correlation coefficients between the colocalized expression of CD68/p16, CD68/p21, vimentin/p16, and vimentin/p21 detected by mIF and clinical characteristics of patients with CSD. Detailed clinical characteristics are provided in Fig. EV. 1h. Color intensity represents the magnitude of the correlation coefficient. *P < 0.05. **d,** Representative mIF images of CSD TMAs (left) and correlation analysis of p16/p21-positive senescent macrophages and p16/p21-positive senescent fibroblasts in a time-course mouse CS model (right) (n = 59-60). Scale bars, 25 μm.

7. The proposed IL-1β/IL-1R axis as the key paracrine mediator is supported by siRNA knockdown experiments, but the absence of in vivo cell-type-specific genetic ablation of Il1b in macrophages or Il1r1 in fibroblasts limits the strength of causal inference.

Response: We agree that establishing a cell type-specific genetic ablation model in vivo would provide stronger evidence for causal inference. However, the primary focus of this study was to define the pathological relevance and translational implications of PGE2-associated senescence in CSD, rather than to comprehensively dissect every molecular component of this signaling cascade. Following the editor's suggestion, we have acknowledged this limitation in the revised **Discussion** (**Page19 Line414-415**) and clarified that definitive validation of this causal relationship will require future studies using cell-type-specific genetic models.

8. The study would benefit from a more rigorous assessment of whether other SASP factors contribute redundantly, as IL-1β blockade only partially reversed fibroblast activation in some assays.

Response: We acknowledge that the potential contribution of other SASP factors was not systematically investigated in the current study. Our bulk RNA-seq data identified IL-1β as a major downstream mediator of PGE2-induced senescence (**Fig. 5e**), and its inhibition effectively attenuates the senescence-associated phenotype (**Fig. 5j-q and Fig. EV7**). These findings support an important role for IL-1β in mediating fibroblast senescence and fibrosis during CSD progression. The partial reversal of fibroblast activation following IL-1β blockade may reflect both the residual activity of IL-1β after siRNA-mediated knockdown or receptor blockade or the contribution of additional SASP factors acting in synergistically. However, a comprehensive dissection of the contribution of individual SASP components was beyond the scope of the current study. We have therefore acknowledged this possibility and discussed the potential involvement of additional factors in the revised **Discussion** (**Page19 Line415-417**).

9. Fig. EV. 7: The two senescence models shown differ from the authors' hypothesis that IL1β induces fibroblast senescence.

Response: The purpose of the experiments presented in **Fig. EV. 8** (Fig. EV. 7 pre) was to define the functional consequence of fibroblast senescence after its induction, rather than to identify the initiating event of fibroblast senescence. In our study, we demonstrated that PGE2-induced *IL1B*$^{+}$ macrophage senescence represents an early inflammatory event that promotes fibroblast senescence through IL-1β/IL-1R signaling, while senescent fibroblasts subsequently acquire autonomous pro-fibrotic activity and further amplify the pathological microenvironment. Consistent with this model, our time-course analyses demonstrated that macrophage senescence emerges earlier during the post-CS inflammatory phase, preceding the accumulation of senescent fibroblasts during the fibrotic remodeling phase (**Fig. 4d-e**). The fibroblast senescence models in updated Fig. EV. 8 revealed that senescent fibroblasts are not merely passive targets of inflammatory signaling but actively propagate pro-fibrotic responses through enhanced contractile activity and fibrotic marker expression. Therefore, these findings extend our hypothesis that IL-1β-induced fibroblast senescence contributes to fibrosis persistence and progression.

10. For clinical trial, the observed improvements in menstrual duration and residual myometrial thickness, while promising, should be interpreted as hypothesis-generating rather than definitive evidence of efficacy. From a mechanistic standpoint, the bulk RNA-seq analysis of patient tissues after metformin treatment shows pathway-level changes but does not directly demonstrate reduced senescent cell burden or PGE2 signaling in the same patients, leaving a gap between the proposed mechanism and clinical response. Finally, the manuscript lacks a clear discussion of potential off-target effects of the anti-aging interventions (e.g., ABT263, metformin) on normal tissue repair or reproductive outcomes beyond fibrosis, which is critical given the uterus's regenerative demands and the known dual roles of PGE2 in muscle homeostasis and inflammation resolution.

Response: We thank the reviewer for this important comment. To further address the mechanistic gap between the proposed mechanism and the clinical observations, we performed additional analyses of tissue samples from metformin-treated patients, including PGE2 ELISA, Masson staining, and IL-1β^{+} γH2AX^{+} mIF analyses. These analyses demonstrated reduced PGE2 levels, collagen deposition, and inflammatory/senescence-associated readouts in metformin-treated CSD and para-CSD tissues (**Fig. 7e-g**). Together, these additional findings provide further evidence supporting the proposed mechanism underlying the observed clinical improvements, while we acknowledge that they do not establish definitive causal relationships. We have also revised the **Discussion** part to address the potential off-target effects of anti-aging interventions, as well as the known dual roles of PGE2 in muscle homeostasis and inflammation resolution (**Page19 Line425-429**).

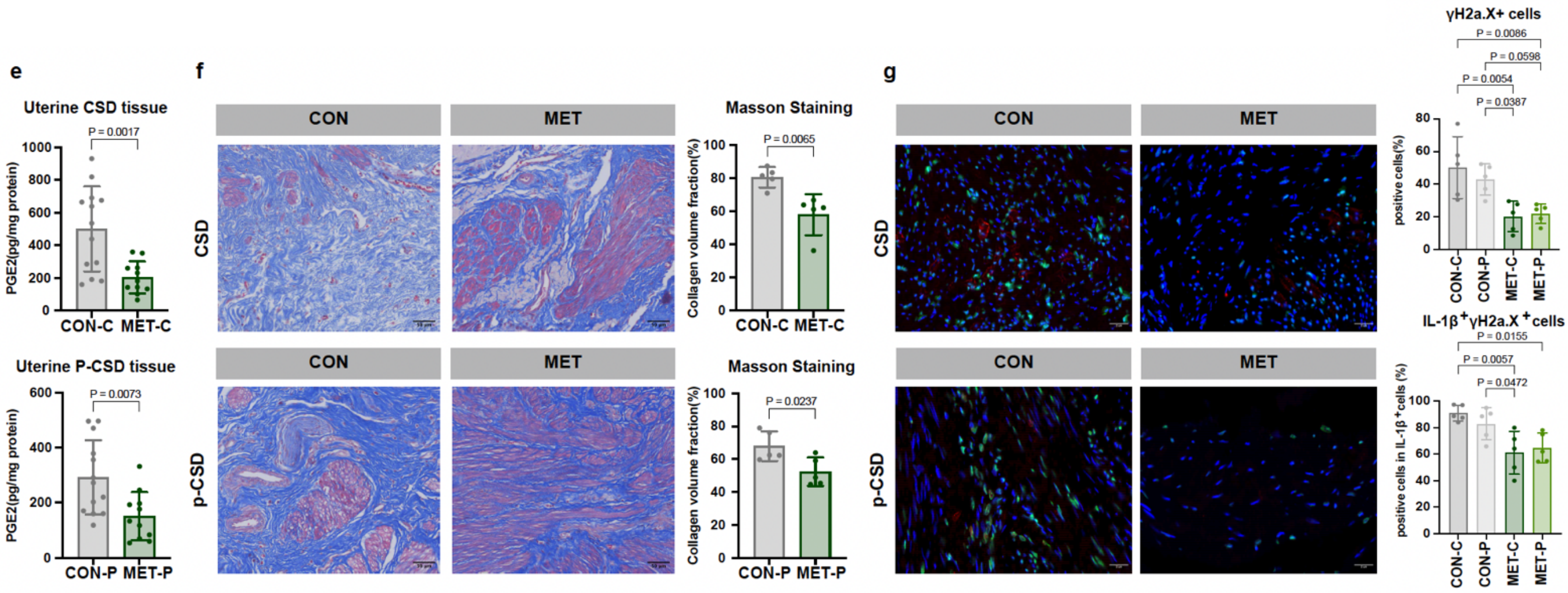

Fig. 7e, ELISA quantification of PGE2 levels in CSD tissues (upper) and para-CSD tissues (lower) from the control and metformin-treated groups (n = 11–13). **f,** Representative Masson staining and quantification of collagen volume fraction in CSD tissues (upper) and para-CSD tissues (lower) from the CON and MET groups (n = 5). Scale bars, 50μm. **g,** Representative mIF staining and quantification of IL-1β^{+}γH2a.X^{+} senescent cells in CSD tissues (upper) and para-CSD tissues (lower) from the CON and MET groups (n = 5). Scale bars, 25μm.

Response to Reviewer 2's Comments

Referee #2 (Comments on Novelty/Model System for Author):

The authors have employed appropriate experimental models and provided a comprehensive analysis of their hypothesis, which is sufficient to support publication of the manuscript. In future studies, the use of genetic approaches to selectively deplete senescent macrophages (inducible depletion model) would be valuable to further delineate their specific contribution to CSD development and progression.

Referee #2 (Remarks for Author):

The manuscript entitled "Prostaglandin E2-mediated aging microenvironment fuels pathogenic fibrosis in cesarean section scar defect" investigates the biological mechanisms underlying cesarean section scar defect (CSD), identifying the PGES/PGE2 signaling pathway as a key driver of fibrosis and impaired uterine healing. The study reveals that elevated PGE2 induces early macrophage senescence, which transmits pro-senescent signals to fibroblasts via IL-1β/IL-1R, leading to excessive fibrosis. Therapeutic interventions targeting PGE2 signaling and cellular senescence; such as inhibitors, senolytics. They further provide evidence that the anti-diabetic drug metformin, effectively improve uterine repair in mouse models and show promising results in a clinical trial. These findings suggest that modulating the senescence and inflammation pathways could enhance healing, prevent CSD and improve fertility outcomes after cesarean delivery.
The manuscript presents a comprehensive and well-structured investigation into the pathogenesis of CSD. The authors conducted a well-designed and comprehensive study, with an impressive integration of metabolomics, transcriptomics, in vitro and in vivo models, as well as clinical trial data. The work provides compelling support for the central hypothesis, identifies key cellular targets, and delineates the molecular pathways involved in disease pathogenesis. The inclusion of clinical samples, a prospective clinical trial with metformin, and validated mouse models strengthen the clinical applicability. The reported findings are novel and carry significant implications for the development of future therapeutic strategies aimed at the prevention of CSD. This manuscript provides a significant contribution to understanding the cellular and molecular mechanisms of CSD and introduces promising therapeutic strategies. The translational potential of targeting this pathway is well-supported, and the combination of experimental and clinical data is a major strength. With minor revisions for clarity, detail, and contextual discussion, it will be a valuable addition to the field. I strongly recommend acceptance of this manuscript to the Journal of EMBO Molecular Medicine after minor revisions.

Some minor comments/suggestions:

The Materials and Methods section is comprehensive, however,
1. Eensure that all experimental procedures, especially animal treatments and clinical trial design, include sufficient detail for reproducibility.
2. Clarify the timing of interventions relative to surgery/injury and repair phases, especially for therapeutic treatments.

Response: We have clarified the experimental procedures, including animal modeling, treatment schedules, sample collection time points, and clinical intervention design in Methods section (**Page24-26 Line551-586**). In addition, the corresponding experimental timelines also have been added to each figure to improve clarity and reproducibility (**Fig. 1i; Fig. 2a/f; Fig. 5o; Fig. 6a/f; Fig. EV7j**).

3. The interim clinical results are promising, however, acknowledge limitations such as sample size, follow-up duration, and potential confounders.
4. Discuss plans for longer-term follow-up and larger randomized studies.

Response: Thank you for these valuable suggestions. We have expanded the **Discussion** section to acknowledge the limitations of the interim clinical analysis. We have also added a statement regarding the necessity of longer-term follow-up and larger multicenter randomized controlled studies (**Page17 Line373-378**).

5. The description of the sham-operated control group is missing from the Methods section.

Response: We added a description of the sham-operated group to the animal models in the Methods (**Page25 Line568-570**).

6. As standard practice, relative gene expression in qPCR experiments is typically normalized using the geometric mean of multiple housekeeping genes (generally three to five) to ensure robust and reliable normalization. In the present study, only ACTB was used as the reference gene (Page 27, line 609). Could the authors provide a justification for this choice? Additionally, please indicate whether the stability of ACTB expression was assessed and confirmed across the patient samples included in the analysis.

Response: We thank the reviewer for this comment. ACTB was selected as the reference gene because it has been widely used for normalization in human uterine tissues [1] and macrophage-related experiments [2] and showed stable expression in our experimental system. We retrospectively examined the raw Ct values of ACTB across the patient samples (shown as below **table 1**). The ACTB expression levels showed no significant differences among patients' groups (shown as below **figure 1**), supporting the stability of ACTB expression in the analyzed samples. Although normalization using multiple validated housekeeping genes may further improve robustness, a single well-validated reference gene remains commonly used in many experimental studies. Based on the stable ACTB expression observed in our cohort and its consistent application in human uterine tissue and macrophage-related experiments, we considered ACTB an appropriate reference gene for the current analysis. In the revised manuscript, we have added a statement in the **Methods** section describing the rationale for selecting ACTB as the reference gene and confirming its expression stability across the analyzed samples (**Page30 Line693-694**).

Group	ACTB	Group	ACTB	Group	ACTB
Control	18.52088292	p-CSD	19.68309466	CSD	21.57107099
Control	22.40941175	p-CSD	19.28292592	CSD	20.44294866
Control	19.07552083	p-CSD	19.36289914	CSD	18.80011686
Control	18.64324125	p-CSD	19.05305290	CSD	20.37487920
Control	21.99660492	p-CSD	22.68721390	CSD	23.97683207
Control	18.96020826	p-CSD	19.31396421	CSD	21.23404821

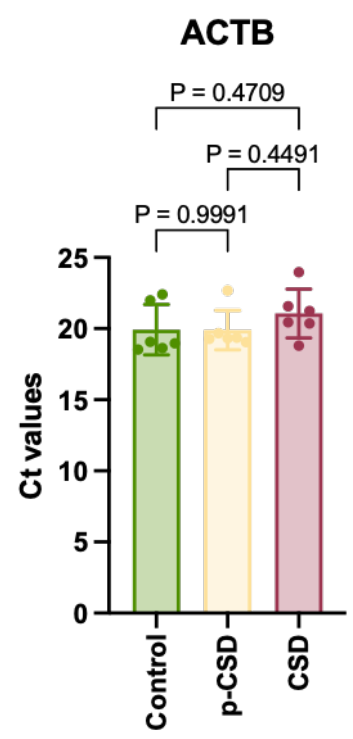

Table 1 and figure 1: Raw Ct values of ACTB in different patients (n=6).

Reference:

[1] Xu B, Geerts D, Bu Z, et al. Regulation of endometrial receptivity by the highly expressed HOXA9, HOXA11 and HOXD10 HOX-class homeobox genes. Human Reproduction. 2014;29(4):781–790. doi:10.1093/humrep/deu004.

[2] Ma J, Wei K, Liu J, et al. Glycogen metabolism regulates macrophage-mediated acute inflammatory responses. Nature Communications. 2020;11:1769. doi:10.1038/s41467-020-15636-8.

Result and Discussion:

7. Fig. 1B: Progesterone levels appear to differ markedly between control, p-CSD, and CSD tissues; yet this is not discussed in the manuscript. The authors should comment on this pronounced difference and discuss its potential biological or clinical relevance. Progesterone is known to modulate inflammatory responses, prostaglandin signaling, angiogenesis, and extracellular matrix remodeling, all of which are key processes in wound repair. Although progesterone is unlikely to be a primary determinant of CSD formation (as it occurs postpartum), altered local progesterone signaling could influence tissue

remodeling and scar maturation. Clarification of whether this difference is mechanistically relevant or a consequence of CSD can help better interpretation of the data.

Response: We thank the reviewer for highlighting the potential biological relevance of the altered progesterone levels in CSD tissues. In the revised manuscript, we performed an expanded targeted metabolomic analysis of arachidonic acid pathway-related metabolites in a larger cohort, and progesterone was additionally included in this validation panel based on its enrichment in healthy control tissues identified by the quasi-targeted metabolomic analysis. The targeted analysis confirmed a consistent decrease in progesterone levels in both CSD and para-CSD tissues compared with healthy controls; however, this difference did not reach statistical significance (**Fig. 1d and Fig. EV1e, supplementary table 1**). Therefore, although altered local progesterone signaling may potentially contribute to tissue remodeling, inflammation, and scar maturation, we cannot determine whether this change represents a mechanistic driver or a secondary consequence of CSD based on the current cross-sectional cohort. In contrast, among the metabolic alterations identified, PGE2 showed a robust and statistically significant elevation in CSD-associated tissues, which was further supported by molecular validation of the PGES/PGE2 axis and functional experiments in vivo and in vitro. Therefore, we focused our mechanistic investigation on PGE2 as a potential driver of CSD-associated fibrotic remodeling. We have added discussion regarding the potential biological relevance of progesterone and other altered metabolites in the revised **Discussion** section (**Page17 Line382-385**), while emphasizing that their causal roles require further investigation. We sincerely appreciate the reviewer's insightful suggestion, which has helped us improve the interpretation of our metabolomic findings.

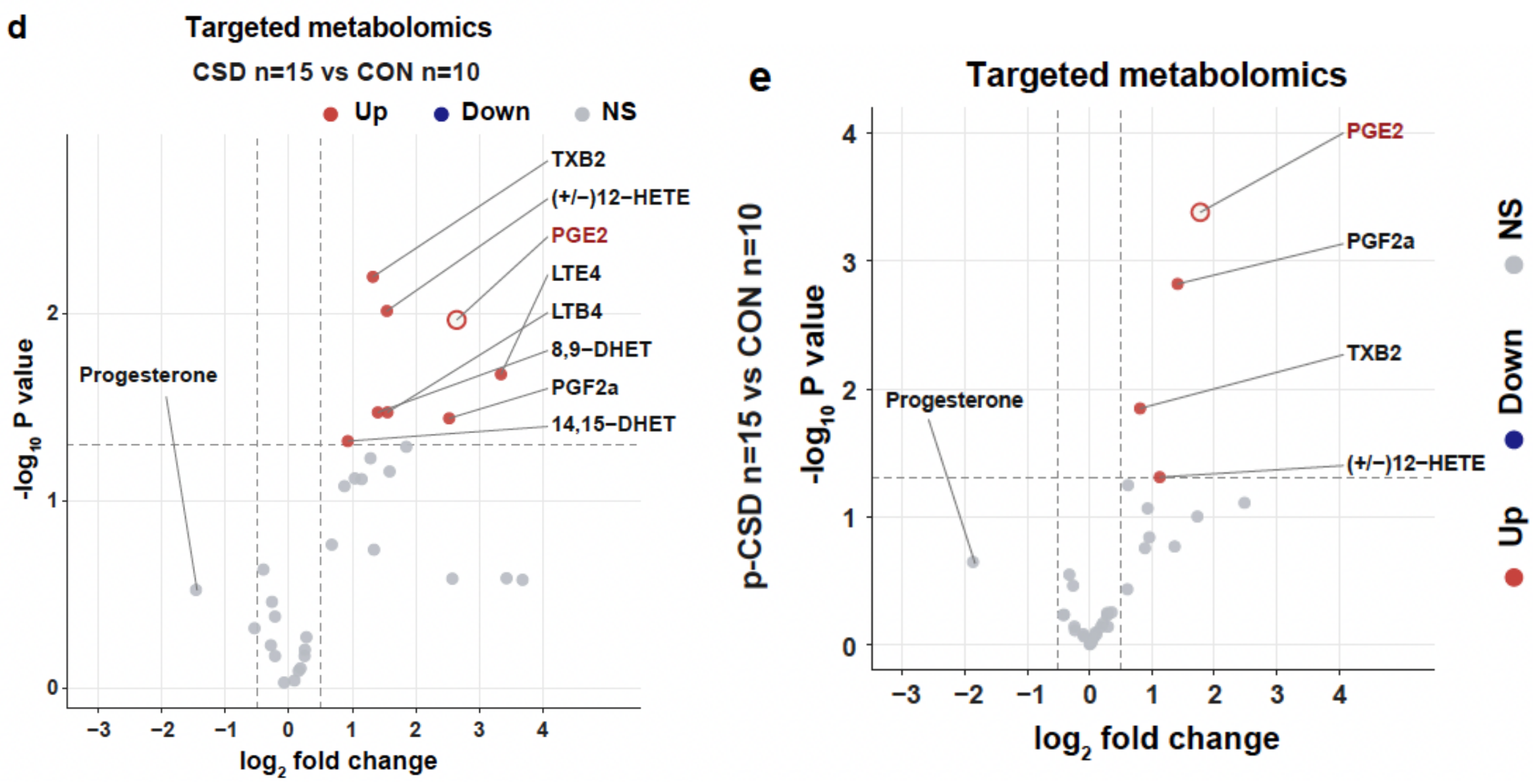

Fig. 1d, Volcano plot showing differential metabolites detected by targeted arachidonic acid metabolomics in CSD tissues (n = 15) and healthy control uterine tissues (n = 10).
Fig. EV1e, Volcano plot showing differential metabolites detected by targeted arachidonic acid metabolomics in para-CSD tissues (n = 15) and healthy control uterine tissues (n = 10).

8. Based on Fig. 1g, the authors may briefly comment on whether serum PGE_2 levels have potential utility as biomarkers for early detection or prognosis of CSD.

Response: In the revised manuscript, we have added a discussion regarding the potential utility of serum PGE2 as a candidate biomarker for CSD detection or disease assessment. We have also emphasized that, although elevated serum PGE2 levels may reflect systemic or local inflammatory alterations associated with CSD, further prospective studies with larger cohorts are required to determine its diagnostic performance, prognostic value, temporal stability, and clinically applicable cutoff values (**Page18 Line389-390**).

9. Differences between p-CSD and CSD: The manuscript does not sufficiently discuss the differences between the p-CSD and CSD groups. The heat map shown in Fig. 1 display distinct expression patterns in these two conditions. The authors should comment on these differences to clarify.

Response: We thank the reviewer for highlighting the potential differences between CSD and para-CSD tissues. Paired para-CSD tissues were included as an intra-patient reference to determine whether the metabolic alterations were confined to the fibrotic scar niche or extended into the surrounding uterine tissue. This paired design also minimizes inter-individual variability arising from factors such as age, hormonal background, and previous cesarean history. By comparing CSD, para-CSD, and healthy control tissues, we aimed to distinguish lesion-specific changes from a broader disease-associated uterine microenvironment. Although the heatmap in Fig. 1b showed visually distinct metabolic patterns between CSD and para-CSD tissues, these differences did not reach statistical significance in the subsequent paired comparison analysis (**Fig. EV. 1d**). Furthermore, targeted metabolomic validation in an expanded cohort confirmed that PGE2 were similarly elevated in both CSD and para-CSD regions compared with healthy controls. These findings suggest that increased PGE2 signaling may represent a broader uterine inflammatory and metabolic alteration associated with CSD patients, rather than a change restricted exclusively to the scar defect region. We have added this interpretation in the revised **Discussion** to clarify the relationship between CSD and para-CSD tissues. (**Page18 Line386-389**).

10. Please discuss if patient-specific factors (e.g., age, hormonal status, surgical techniques) influence PGE2 levels, senescence, and fibrosis in CSD?

Response: We have carefully reviewed the baseline clinical characteristics of our cohort (**Supplementary Table 3**). There were no significant differences between the control and CSD groups in age (33.78 ± 3.87 vs. 33.44 ± 3.98 years, P = 0.728), BMI (21.43 ± 2.57 vs. 21.37 ± 2.63 kg/m², P = 0.915), blood glucose levels (5.00 ± 0.50 vs. 4.99 ± 0.54 mmol/L, P = 0.993), or cesarean section history (1.26 ± 0.45 vs. 1.37 ± 0.54, P = 0.411). Notably, all individuals in the control group had experienced at least one previous cesarean delivery, which minimizes the potential confounding effect of prior cesarean-associated uterine remodeling. Regarding surgical factors, cesarean section procedures in our cohort were performed under relatively standardized clinical conditions. In current clinical practice in China, double-layer uterine closure is routinely adopted, and therefore major variations in suturing techniques were unlikely to represent a major source of heterogeneity in our cohort. Nevertheless, we acknowledge that the potential contribution of hormonal status to PGE2 signaling and fibrosis in CSD remains unclear. As systematic hormonal measurements were not routinely collected in this surgical cohort, we were unable to directly evaluate their potential influence. We have acknowledged this limitation and added a discussion of the potential impact of hormonal status and patient heterogeneity in the revised **Discussion** section (**Page17 Line384-385**).

11. The rationale for administering metformin one month prior to VR surgery (Fig. 7A, page 14, line 303) is unclear. This design appears inconsistent with the murine experiments, in which metformin demonstrated a significant therapeutic effect even when administered post surgery (Fig. 6F-J). The authors should clarify the reasoning behind the preoperative treatment strategy for women and explain why a post-surgical intervention, which more closely aligned with the assessed animal model, was not evaluated.

Response: We thank the reviewer for raising this important point regarding the timing of metformin administration. Unlike the mouse CS model, in which uterine injury is experimentally induced in otherwise healthy animals, patients enrolled in our clinical trial already presented with an established CSD-associated pathological microenvironment characterized by chronic inflammation, fibrosis, and senescence-associated changes. Therefore, metformin was initiated one month before VR surgery to precondition the pathological niche and potentially improve the regenerative environment before surgical reconstruction. And then, metformin treatment was continued for six months after VR surgery. This postoperative treatment duration was selected because it corresponds to the clinically relevant period of CSD formation after cesarean section [3]. Thus, although the timing of intervention differs between the mouse injury model and the clinical CSD setting, both

approaches were designed to target the critical period of senescence-associated tissue remodeling. We have clarified the rationale in the revised **Discussion** sections (**Page17 Line365-369**).

[3] Post BL, Verberkt C, Hup RJ, et al. Niche presence and changes of the uterine scar in the first year after caesarean section: A prospective longitudinal study. European Journal of Obstetrics & Gynecology and Reproductive Biology. 2026;321:115051. doi:10.1016/j.ejogrb.2026.115051.

12. A proportion of pregnant women with pre existing type 2 diabetes are treated with metformin prior to conception, and metformin is also commonly prescribed for the management of gestational diabetes mellitus (GDM). It is well established that cesarean section delivery and CSD is more frequent among women with diabetes, largely attributed to impaired wound healing. Both GDM and pre existing diabetes have been identified as significant patient related risk factors for postoperative wound complications. In this context, it remains unclear why metformin does not appear to confer a protective benefit in this population. Could the authors comment on this apparent lack of benefit? Additionally, have patient level data been analyzed to specifically assess this association?

Response: We thank the reviewer for raising this important point. We agree that diabetes is an important determinant of wound repair and a potential confounder. In our clinical study, patients with diabetes and abnormal blood glucose were excluded, and therefore our study was not designed to evaluate whether metformin provides a protective effect against CSD in diabetic or GDM populations. In addition, baseline metabolic characteristics, including BMI, were comparable between the analyzed groups, reducing the potential influence of major metabolic differences within our study population (**Fig. 7d**). We have clarified these eligibility criteria in **methods** (**Page24 Line539-543**) and added this discussion in the revised manuscript (**Page17 Line375-377**).

13. Metformin is a pleiotropic drug with multiple cellular and metabolic targets. In the context of CSD, the specific mechanism/s by which metformin mediates the observed beneficial effects remain unclear. The authors should comment on the possible mechanism of action of metformin in the context of CSD.

Response: We thank the reviewer for this comment regarding the pleiotropic effects of metformin. In this study, metformin was selected based on its established clinical application, favorable safety profile, and emerging role as a senomorphic agent capable of alleviating chronic inflammation and aging-associated pathological microenvironments. In the revised manuscript, we further strengthened the mechanistic interpretation of metformin in CSD. Our previous transcriptomic analysis of metformin-treated CSD tissues revealed suppression of pathways associated with PGE2 signaling, cellular senescence, inflammatory responses, and fibrotic remodeling. To further validate these observations, we have now added additional evidence, including reduced PGE2 levels by ELISA, decreased collagen deposition by Masson staining, and reduced IL-1$\beta^{+}\gamma$H2AX^{+} senescent cells by mIF in metformin-treated CSD and para-CSD tissues (**Fig. 7e-g**). Together, these findings support the interpretation that metformin exerts beneficial effects in CSD, at least in part, by attenuating the PGE2-associated inflammatory senescence program and subsequent fibrotic remodeling.

At the same time, we acknowledge that metformin has multiple cellular and metabolic targets, and therefore cannot exclude the involvement of other mechanisms, including AMPK signaling, mitochondrial homeostasis, oxidative stress, and other inflammatory pathways, which may collectively contribute to tissue repair [4-6]. Nevertheless, our findings support the PGE2-associated inflammatory senescence pathway as an important mechanism underlying the beneficial effects of metformin in CSD. We have incorporated these potential mechanisms into the revised Discussion while emphasizing that their relative contribution remains to be determined (**Page17 Line371-373**).

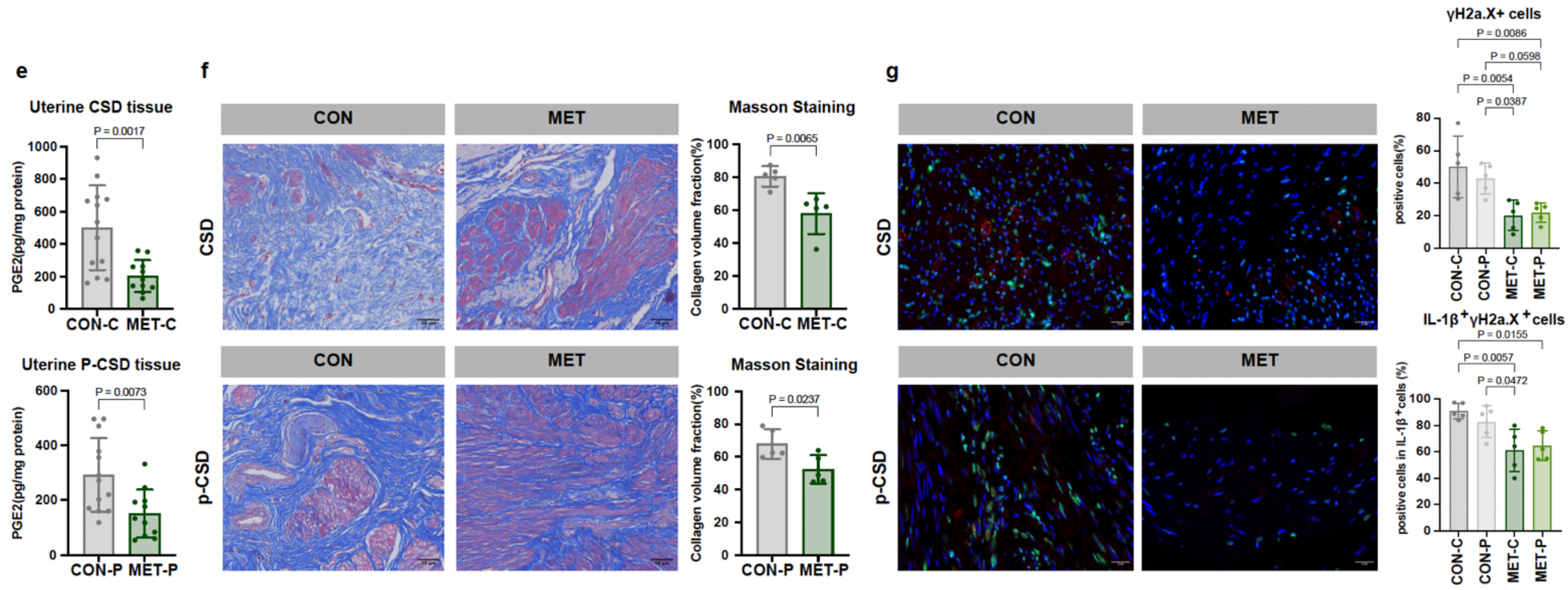

Fig. 7e, ELISA quantification of PGE2 levels in CSD tissues (upper) and para-CSD tissues (lower) from the control and metformin-treated groups (n = 11–13). **f,** Representative Masson staining and quantification of collagen volume fraction in CSD tissues (upper) and para-CSD tissues (lower) from the CON and MET groups (n = 5). Scale bars, 50μm. **g,** Representative mIF staining and quantification of $IL\text{-}1\beta^{+}\gamma H2a.X^{+}$ senescent cells in CSD tissues (upper) and para-CSD tissues (lower) from the CON and MET groups (n = 5). Scale bars, 25μm.

Reference:

[4] Foretz, M., Guigas, B. & Viollet, B. Metformin: update on mechanisms of action and repurposing potential. Nat Rev Endocrinol 19, 460–476 (2023). https://doi.org/10.1038/s41574-023-00833-4

[5] Herzig S, Shaw RJ. AMPK: guardian of metabolism and mitochondrial homeostasis. Nat Rev Mol Cell Biol. 2018 Feb;19(2):121-135. doi: 10.1038/nrm.2017.95.

[6] Foretz, M., Guigas, B. & Viollet, B. Understanding the glucoregulatory mechanisms of metformin in type 2 diabetes mellitus. Nat Rev Endocrinol 15, 569–589 (2019). https://doi.org/10.1038/s41574-019-0242-2

14. While agents such as celecoxib and CAY10526 demonstrate efficacy in the mouse model, both are broad inhibitors of prostaglandin synthesis. As such, their effects may not be specific to pathways directly implicated in CSD pathogenesis. Potential off-target actions and systemic consequences, particularly in a clinical setting, warrant further discussion. The authors should address the limitations of broadly suppressing prostaglandin signaling and discuss the potential safety concerns and translational challenges associated with these agents in humans.

Response: We sincerely appreciate the reviewer's insightful suggestion. In the revised manuscript, we have expanded the **Discussion** to address the potential off-target effects and clinical challenges of these interventions. And we emphasize that timing, dose, tissue specificity, and reproductive safety require further study before clinical translation (**Page19 Line425-429**).

15. The clinical trial is an interim analysis with a small sample size (30 patients). Long-term outcomes, fertility data, and adverse event monitoring are necessary before definitive conclusions. Details on randomization, blinding, and controls should be mentioned to assess bias.

Response: We thank the reviewer for this important comment. We have clarified that this study represents an interim analysis of the registered trial. The detailed clinical study protocol has also been provided as a supplementary file for transparency. We have revised the Discussion sections to emphasize that these findings are preliminary and based on a six-month interim analysis of 30 participants. The limitations related to sample size, single-center design, open-label nature, and the need for longer-term follow-up of fertility outcomes, pregnancy outcomes, and reproductive safety have been added to the revised **Discussion** (**Page17 Line373-378**).

Respectfully，

Xipeng Wang
Department of Obstetrics and Gynecology, Xinhua Hospital Affiliated to Shanghai Jiao Tong University School of Medicine, 1665 Kongjiang Road, Shanghai 200092, China.
E-mail: wangxipeng@xinhuamed.com.cn

Ziyi Li, PhD
National Key Laboratory of Immunity and Inflammation, Suzhou Institute of Systems Medicine, Chinese Academy of Medical Sciences & Peking Union Medical College, Suzhou 215123, Jiangsu, China.
E-mail: lzy@ism.pumc.edu.cn

19th Aug 2026

Dear Prof. Wang,

Thank you again for submitting your revised manuscript. We have now received reports from the two referees who were asked to evaluate the revised study.

As you will see from the reports below, Reviewer #1 continues to raise important concerns regarding the clinical trial component of the study. In line with Reviewer #1's suggestion, we ask that you either remove the entire clinical intervention data from the Results section, together with the corresponding Methods, or include the relevant results in the supplementary material with the limitations explicitly discussed and the conclusions significantly toned down to avoid any potential overinterpretation of these findings.

On a more editorial level:

1. If you decide to include the clinical studies in an Appendix, the figures and tables should be bundled together with their legends in a single PDF file entitled Appendix, beginning with a Table of Contents for all the listed items. Each legend should appear directly below the corresponding figure or table. Appendix figures and tables should be cited in the main text as "Appendix Figure S1," "Appendix Figure S2," "Appendix Table S1," etc.

2. Please remove the "Authors' contribution" section from the manuscript text.

3. Please remove the figures from the manuscript file.

4. "Competing interests" should be renamed to "Disclosure and competing interests statement".

5. Please use our Reagents and Tools Table template (.docx), which you can find in our author guidelines: https://link.springer.com/journal/44320/submission-guidelines#structuredmethods.

When submitting your revised manuscript, please do not include the Reagents and Tools Table in the Methods section of the manuscript but upload it as a separate file choosing the file type "Reagent Table".

6. The references need to be formatted according to the EMBO Molecular Medicine reference style. Please list up to 10 co-authors of a paper before adding et al. in the reference list. Citations should be listed in alphabetical order.

7. In Methods, for human studies, include a statement that the experiments conformed to the principles set out in the WMA Declaration of Helsinki and the Department of Health and Human Services Belmont Report.

8. Please incorporate the funding information into the "Acknowledgements" section and remove the separate "Funding" heading.

9. Please provide "The paper explained" in the manuscript file : EMBO Molecular Medicine articles are accompanied by a summary of the articles to emphasize the major findings in the paper and their medical implications for the non-specialist reader.

- the medical issue you are addressing,

- the results obtained and

- their clinical impact.

Please include separate subsections entitled Problem, Results, and Impact, in line with the journal format. Please refer to published papers for examples.

10. Please remove "The Highlight" and the "List of Supplementary Materials" from the manuscript text.

11. Remove the file named "Supplementary figures".

12. Please rename Supplementary Table 1, containing the metabolomics data, as "Dataset EV1," and Supplementary Table 2, containing the patients' sampling metadata, as "Dataset EV2." Please update all corresponding callouts in the manuscript accordingly.

Please also include the legend for each dataset within the respective file, on a separate sheet labelled "Legend."

13. During our routine image check, we noted potential image reuse within Figure 3C, specifically between the IL-1β and CCL2

control panels(round view). If the same sample was used for both panels, please state this explicitly in the figure legend to avoid any potential confusion for readers.

14. Data Availability section:

- The scRNA-seq data from normal human uterine tissues should also be made publicly available prior to acceptance of the manuscript. Please remove the statement, "The scRNA-seq data of normal human uterine tissues is available from the corresponding author upon reasonable request," and instead provide the database name, accession number(s), and a specific, resolvable URL for the dataset.
- For the bulk RNA-seq data, please ensure that the datasets will be made publicly available upon acceptance of the manuscript.

15. Please provide a 'Synopsis' to further enhance discoverability. Synopses are displayed on the journal webpage and are freely accessible to all readers. They include a short stand first (maximum of 300 characters, including space) as well as 2-5 one-sentences bullet points that summarizes the paper. Please write the bullet points to summarize the key NEW findings. They should be designed to be complementary to the abstract - i.e. not repeat the same text. We encourage inclusion of key acronyms and quantitative information (maximum of 30 words / bullet point). Please use the passive voice. Please attach these in a separate file or send them by email, we will incorporate them accordingly.

Please also provide a visual abstract as a separate PNG file with dimensions of exactly 550 px (width) × 300 px (height) to highlight the paper on our homepage.

16. Please address the following issues in the figure legends, as identified by our Data Editor:
- Please note that the exact p values are not provided in the legends of figures 1K, M; 2B, D, E; 3B, D, E; 4B, C; 5C, D, I, M, N; 6E, EV2 A, B, D, G; EV3 K, EV4 B, E, F, G-K; EV5 A, B; EV6, EV7 B, C, F, EV8C, D; EV10A
- Please indicate the statistical test used for data analysis in the legends of figures 1B, C; 5E, G; EV1 E, EV6 A, D
- Please note that the box plots need to be defined in terms of minima, maxima, centre, bounds of box and whiskers, and percentile in the legends of figures 5G, EV6F
- Please note that information related to n is missing in the legends of figures EV1 D, F
- Please note that for heatmap present in figure 1E a numbered scale bar is not provided. This needs to be rectified.
- Please note that the scale bar needs to be defined for figure 5N.

17. Please update the order and the headings of the manuscript sections to: Abstract / The Paper Explained / Introduction /Results / Discussion / Methods / Data Availability / Acknowledgements / Disclosure and Competing Interests Statement / References / Figure Legends / Expanded View Figure Legends

I look forward to reading a new revised version of your manuscript as soon as possible.

Sincerely,
Jingyi

Jingyi Hou
Senior Editor
EMBO Molecular Medicine

*** Instructions to submit your revised manuscript ***

*** PLEASE NOTE *** As part of the EMBO Publications transparent editorial process initiative (see our Editorial at https://www.embopress.org/doi/pdf/10.1002/emmm.201000094), EMBO Molecular Medicine will publish online a Review Process File to accompany accepted manuscripts.

In the event of acceptance, this file will be published in conjunction with your paper and will include the anonymous referee reports, your point-by-point response and all pertinent correspondence relating to the manuscript. If you do NOT want this file to be published, please inform the editorial office at contact@embomolmed.org.

*****

***** Reviewer's comments *****

Referee #1 (Remarks for Author):

I have carefully reviewed the authors' point by point responses and the revised manuscript, and I sincerely commend their extraordinary efforts in adding primary human macrophage validation, multiplex immunofluorescence co staining, and expanded metabolomics, which have greatly strengthened the mechanistic core. However, two critical flaws remain in the clinical translation: (1) the open label, single arm trial lacks a placebo/surgery only control, making it impossible to attribute improvements to metformin versus the surgical procedure itself; and (2) the mechanistic pathway was validated with COX 2 inhibitors, whereas the clinical drug is metformin-whose link to PGE2 suppression is only correlative and not causally established. Given these irremediable design limitations, I strongly advise removing the entire clinical intervention dataset (Fig. 7 and related text) from the main manuscript. This would sharpen the narrative around the robust preclinical mechanism (mouse models with COX 2 inhibitors/senolytics) and avoid overinterpretation; the uncontrolled clinical observation could be briefly mentioned in the Discussion as a hypothesis generating note. With this strategic excision, I believe the paper will be fully acceptable for publication.

Referee #2 (Remarks for Author):

The authors have satisfactorily addressed all comments, questions, and revision requests raised during the review process. I am satisfied with the responses and the revisions made to the manuscript. In my opinion, the manuscript is now suitable for publication and may be accepted.

Review Response Letter

Response to Reviewer 1's Comments

Referee #1 (Remarks for Author):

I have carefully reviewed the authors' point by point responses and the revised manuscript, and I sincerely commend their extraordinary efforts in adding primary human macrophage validation, multiplex immunofluorescence co staining, and expanded metabolomics, which have greatly strengthened the mechanistic core. However, two critical flaws remain in the clinical translation: (1) the open label, single arm trial lacks a placebo/surgery only control, making it impossible to attribute improvements to metformin versus the surgical procedure itself; and (2) the mechanistic pathway was validated with COX 2 inhibitors, whereas the clinical drug is metformin-whose link to PGE2 suppression is only correlative and not causally established. Given these irremediable design limitations, I strongly advise removing the entire clinical intervention dataset (Fig. 7 and related text) from the main manuscript. This would sharpen the narrative around the robust preclinical mechanism (mouse models with COX 2 inhibitors/senolytics) and avoid overinterpretation; the uncontrolled clinical observation could be briefly mentioned in the Discussion as a hypothesis generating note. With this strategic excision, I believe the paper will be fully acceptable for publication.

Response: We sincerely thank you for the careful evaluation of our revised manuscript and for the constructive recommendation. In accordance with your suggestion, we have removed the entire clinical intervention dataset, including Figure 7, Figure EV10 and all related Methods, Results, and Discussion text, from the revised manuscript.

Response to Reviewer 2's Comments

Referee #2 (Remarks for Author):

The authors have satisfactorily addressed all comments, questions, and revision requests raised during the review process. I am satisfied with the responses and the revisions made to the manuscript. In my opinion, the manuscript is now suitable for publication and may be accepted.

Response: We sincerely thank you for the positive assessment of our revised manuscript and for recognizing our efforts in addressing the previous comments. We greatly appreciate the reviewer’s time and thoughtful evaluation.

Respectfully，

Xipeng Wang
Department of Obstetrics and Gynecology, Xinhua Hospital Affiliated to Shanghai Jiao Tong University School of Medicine, 1665 Kongjiang Road, Shanghai 200092, China.
E-mail: wangxipeng@xinhuamed.com.cn

Ziyi Li, PhD
National Key Laboratory of Immunity and Inflammation, Suzhou Institute of Systems Medicine, Chinese Academy of Medical Sciences & Peking Union Medical College, Suzhou 215123, Jiangsu, China.
E-mail: lzy@ism.pumc.edu.cn

2nd Sep 2026

Dear Prof. Wang,

We are pleased to inform you that your manuscript is accepted for publication and is now being sent to our publisher to be included in the next available issue of EMBO Molecular Medicine.

Your manuscript will be processed for publication by EMBO Press. It will be copy edited and you will receive page proofs prior to publication. Please note that you will be contacted by Springer Nature Author Services to complete licensing and payment information.

You may qualify for financial assistance for your publication charges - either via a Springer Nature fully open access agreement or an EMBO initiative. Check your eligibility: https://link.springer.com/journal/44321/how-to-publish-with-us

Should you be planning a Press Release on your article, please notify the production team via this form in order to coordinate publication and release dates: https://forms.cloud.microsoft/e/eH4A8JVP6g

If you have any questions, please do not hesitate to contact the Editorial Office. Thank you for your contribution to EMBO Molecular Medicine.

Yours sincerely,
Jingyi

Jingyi Hou
Senior Editor
EMBO Molecular Medicine

>>> Please note that it is EMBO Molecular Medicine policy for the transcript of the editorial process (containing referee reports and your response letter) to be published as an online supplement to each paper. If you do NOT want this, you will need to inform the Editorial Office via email immediately. More information is available here: https://link.springer.com/partners/embo-press/editorial-policies#Peer%20review

EMBO Press Author Checklist

Corresponding Author Name: Xipeng Wang, Ziyi Li	
Journal Submitted to: EMBO Molecular Medicine	
Manuscript Number: EMM-2026-23701	

USEFUL LINKS FOR COMPLETING THIS FORM
The EMBO Journal - Author Guidelines
EMBO Reports - Author Guidelines
Molecular Systems Biology - Author Guidelines
EMBO Molecular Medicine - Author Guidelines

Reporting Checklist for Life Science Articles (updated January
This checklist is adapted from Materials Design Analysis Reporting (MDAR) Checklist for Authors. MDAR establishes a minimum set of requirements in transparent reporting in the life sciences (see Statement of Task: 10.31222/osf.io/9sm4x). Please follow the journal's guidelines in preparing your
Please note that a copy of this checklist will be published alongside your article.

Abridged guidelines for figures
1. Data
The data shown in figures should satisfy the following conditions:
- the data were obtained and processed according to the field's best practice and are presented to reflect the results of the experiments in an accurate and unbiased manner.
- ideally, figure panels should include only measurements that are directly comparable to each other and obtained with the same assay.
- plots include clearly labeled error bars for independent experiments and sample sizes. Unless justified, error bars should not be shown for technical
- if n<5, the individual data points from each experiment should be plotted. Any statistical test employed should be justified.
- Source Data should be included to report the data underlying figures according to the guidelines set out in the authorship guidelines on Data

2. Captions
Each figure caption should contain the following information, for each panel where they are relevant:
- a specification of the experimental system investigated (eg cell line, species name).
- the assay(s) and method(s) used to carry out the reported observations and measurements.
- an explicit mention of the biological and chemical entity(ies) that are being measured.
- an explicit mention of the biological and chemical entity(ies) that are altered/varied/perturbed in a controlled manner.
- the exact sample size (n) for each experimental group/condition, given as a number, not a range;
- a description of the sample collection allowing the reader to understand whether the samples represent technical or biological replicates (including how many animals, litters, cultures, etc.).
- a statement of how many times the experiment shown was independently replicated in the laboratory.
- definitions of statistical methods and measures:
  - common tests, such as t-test (please specify whether paired vs. unpaired), simple $\chi 2$ tests, Wilcoxon and Mann-Whitney tests, can be unambiguously identified by name only, but more complex techniques should be described in the methods section;
  - are tests one-sided or two-sided?
  - are there adjustments for multiple comparisons?
  - exact statistical test results, e.g., P values = x but not P values < x;
  - definition of 'center values' as median or average;
  - definition of error bars as s.d. or s.e.m.

Please complete ALL of the questions below.
Select "Not Applicable" only when the requested information is not relevant for your study.

Materials

Newly Created Materials	Information included in the manuscript?	In which section is the information available? (Reagents and Tools Table, Materials and Methods, Figures, Data Availability Section)
New materials and reagents need to be available; do any restrictions apply?	Not Applicable	

Antibodies	Information included in the manuscript?	In which section is the information available? (Reagents and Tools Table, Materials and Methods, Figures, Data Availability Section)
For **antibodies** provide the following information:<br>- Commercial antibodies: RRID (if possible) or supplier name, catalogue number and or/clone number<br>- Non-commercial: RRID or citation	Yes	Supplemental table 2

DNA and RNA sequences	Information included in the manuscript?	In which section is the information available? (Reagents and Tools Table, Materials and Methods, Figures, Data Availability Section)
**Short novel DNA or RNA including primers, probes**: provide the sequences.	Yes	Supplemental table 2

Cell materials	Information included in the manuscript?	In which section is the information available? (Reagents and Tools Table, Materials and Methods, Figures, Data Availability Section)
**Cell lines:** Provide species information, strain. Provide accession number in repository **OR** supplier name, catalog number, clone number, and/**OR** RRID.	Yes	Material and Methods
**Primary cultures:** Provide species, strain, sex of origin, genetic modification status.	Yes	Material and Methods
Report if the cell lines were recently **authenticated** (e.g., by STR profiling) and tested for mycoplasma contamination.	Yes	Material and Methods

Experimental animals	Information included in the manuscript?	In which section is the information available? (Reagents and Tools Table, Materials and Methods, Figures, Data Availability Section)
**Laboratory animals or Model organisms:** Provide species, strain, sex, age, genetic modification status. Provide accession number in repository **OR** supplier name, catalog number, clone number, **OR** RRID.	Yes	Material and Methods
**Animal observed in or captured from the field:** Provide species, sex, and age where possible.	Not Applicable	
Please detail **housing and husbandry conditions**.	Yes	Material and Methods

Plants and microbes	Information included in the manuscript?	In which section is the information available? (Reagents and Tools Table, Materials and Methods, Figures, Data Availability Section)
**Plants:** provide species and strain, ecotype and cultivar where relevant, unique accession number if available, and source (including location for collected wild specimens).	Not Applicable	
**Microbes:** provide species and strain, unique accession number if available, and source.	Not Applicable	

Human research participants	Information included in the manuscript?	In which section is the information available? (Reagents and Tools Table, Materials and Methods, Figures, Data Availability Section)
If collected and within the bounds of privacy constraints report on age, sex and gender or ethnicity for all study participants.	Yes	Material and Methods; Supplemental table 3

Core facilities	Information included in the manuscript?	In which section is the information available? (Reagents and Tools Table, Materials and Methods, Figures, Data Availability Section)
If your work benefited from core facilities, was their service mentioned in the acknowledgments section?	Yes	Acknowledgments

Design

Study protocol	Information included in the manuscript?	In which section is the information available? (Reagents and Tools Table, Materials and Methods, Figures, Data Availability Section)
If study protocol has been **pre-registered, provide DOI in the manuscript**. For clinical trials, provide the trial registration number **OR** cite DOI.	Not Applicable	Supplementary files: study protocol
Report the **clinical trial registration number** (at ClinicalTrials.gov or equivalent), where applicable.	Yes	Material and Methods

Laboratory protocol	Information included in the manuscript?	In which section is the information available? (Reagents and Tools Table, Materials and Methods, Figures, Data Availability Section)
Provide DOI OR other citation details if **external detailed step-by-step protocols** are available.	Yes	Material and Methods

Experimental study design and statistics	Information included in the manuscript?	In which section is the information available? (Reagents and Tools Table, Materials and Methods, Figures, Data Availability Section)
Include a statement about **sample size** estimate even if no statistical methods were used.	Yes	Supplementary files: study protocol
Were any steps taken to minimize the effects of subjective bias when allocating animals/samples to treatment (e.g. **randomization procedure**)? If yes, have they been described?	Yes	Supplementary files: study protocol
Include a statement about **blinding** even if no blinding was done.	Yes	Supplementary files: study protocol
Describe **inclusion/exclusion criteria** if samples or animals were excluded from the analysis. Were the criteria pre-established?<br><br>If sample or data points were omitted from analysis, report if this was due to attrition or intentional exclusion and provide justification.	Yes	Supplementary files: study protocol;Material and Methods
For every figure, are **statistical tests** justified as appropriate? Do the data meet the assumptions of the tests (e.g., normal distribution)? Describe any methods used to assess it. Is there an estimate of variation within each group of data? Is the variance similar between the groups that are being statistically compared?	Yes	Material and Methods

Sample definition and in-laboratory replication	Information included in the manuscript?	In which section is the information available? (Reagents and Tools Table, Materials and Methods, Figures, Data Availability Section)
In the figure legends: state number of times the experiment was **replicated** in laboratory.	Yes	Figure legends
In the figure legends: define whether data describe **technical or biological replicates**.	Yes	Figure legends

Ethics

Ethics	Information included in the manuscript?	In which section is the information available? (Reagents and Tools Table, Materials and Methods, Figures, Data Availability Section)
Studies involving **human participants**: State details of **authority granting ethics approval** (IRB or equivalent committee(s), provide reference number for approval.	Yes	Material and Methods
Studies involving **human participants**: Include a statement confirming that **informed consent** was obtained from all subjects and that the experiments conformed to the principles set out in the WMA Declaration of Helsinki and the Department of Health and Human Services Belmont Report.	Yes	Material and Methods
Studies involving **human participants: For publication of patient photos**, include a statement confirming that consent to publish was obtained.	Not Applicable	
Studies involving experimental **animals**: State details of **authority granting ethics approval** (IRB or equivalent committee(s), provide reference number for approval. Include a statement of compliance with ethical regulations.	Yes	Material and Methods
Studies involving **specimen and field samples:** State if relevant **permits** obtained, provide details of authority approving study; if none were required, explain why.	Not Applicable	

Dual Use Research of Concern (DURC)	Information included in the manuscript?	In which section is the information available? (Reagents and Tools Table, Materials and Methods, Figures, Data Availability Section)
Could your study fall under dual use research restrictions? Please check biosecurity documents and list of **select agents and toxins** (CDC): https://www.selectagents.gov/sat/list.htm	Not Applicable	
If you used a select agent, is the security level of the lab appropriate and reported in the manuscript?	Not Applicable	
If a study is subject to dual use research of concern regulations, is the name of the **authority granting approval and reference number** for the regulatory approval provided in the manuscript?	Not Applicable	

Reporting

The MDAR framework recommends adoption of discipline-specific guidelines, established and endorsed through community initiatives. Journals have their own policy about requiring specific guidelines and recommendations to complement MDAR.

Adherence to community standards	Information included in the manuscript?	In which section is the information available? (Reagents and Tools Table, Materials and Methods, Figures, Data Availability Section)
State if relevant guidelines or checklists (e.g., **ICMJE, MIBBI, ARRIVE, PRISMA**) have been followed or provided.	Not Applicable	
For **tumor marker prognostic studies,** we recommend that you follow the **REMARK** reporting guidelines (see link list at top right). See author guidelines, under 'Reporting Guidelines'. Please confirm you have followed these guidelines.	Not Applicable	
For **phase II and III randomized controlled trials**, please refer to the **CONSORT** flow diagram (see link list at top right) and submit the CONSORT checklist (see link list at top right) with your submission. See author guidelines, under 'Reporting Guidelines'. Please confirm you have submitted this list.	Not Applicable	

Data Availability

Data availability	Information included in the manuscript?	In which section is the information available? (Reagents and Tools Table, Materials and Methods, Figures, Data Availability Section)
Have **primary datasets** been deposited according to the journal's guidelines (see 'Data Deposition' section) and the respective accession numbers provided in the Data Availability Section?	Yes	Data Availability
Were **human clinical and genomic datasets** deposited in a public access-controlled repository in accordance to ethical obligations to the patients and to the applicable consent agreement?	Yes	Data Availability
Are **computational models** that are central and integral to a study available without restrictions in a machine-readable form? Were the relevant accession numbers or links provided?	Not Applicable	
If publicly available data were reused, provide the respective **data citations in the reference list**.	Yes	Material and Methods
